# The Cytotoxic Activity of Dammarane-Type Triterpenoids Isolated from the Stem Bark of *Aglaia cucullata* (Meliaceae)

**DOI:** 10.3390/molecules28134946

**Published:** 2023-06-23

**Authors:** Purnama, Kindi Farabi, Dudi Runadi, Hadi Kuncoro, Desi Harneti, Nurlelasari, Tri Mayanti, Mohamad Nurul Azmi, Sofa Fajriah, Unang Supratman

**Affiliations:** 1 Department of Chemistry, Faculty of Mathematics and Natural Sciences, Universitas Padjadjaran, Sumedang 45363, West Java, Indonesia; prnmkiunpad123@gmail.com (P.); kindi.farabi@unpad.ac.id (K.F.); dudi.runadi@unpad.ac.id (D.R.); desi.harneti@unpad.ac.id (D.H.); nurlelasari@unpad.ac.id (N.); t.mayanti@unpad.ac.id (T.M.); 2 Central Laboratory, Universitas Padjadjaran, Sumedang 45363, West Java, Indonesia; 3 Faculty of Pharmacy, Universitas Mulawarman, Samarinda 75123, East Kalimantan, Indonesia; hadikuncoro@farmasi.unmul.ac.id; 4 School of Chemical Sciences, Universiti Sains Malaysia, Minden 11800, Penang, Malaysia; mnazmi@usm.my; 5 Research Center for Pharmaceutical Ingredients and Traditional Medicine, National Research and Innovation Agency (BRIN), Complex Cibinong Science Center–BRIN, Cibinong 16911, Jawa Barat, Indonesia; sofafajriah@gmail.com

**Keywords:** *Aglaia cucullata*, cytotoxic activity, MCF-7 cell line, B16-F10 cell line, CV-1 normal cell line

## Abstract

The *Aglaia* genus, a member of the Meliaceae family, is generally recognized to include a number of secondary metabolite compounds with diverse structures and biological activities, including triterpenoids. Among the members of this genus, *Aglaia cucullata* has been reported to have unique properties and thrives exclusively in mangrove ecosystems. This plant is also known to contain various metabolites, such as flavaglines, bisamides, and diterpenoids, but there are limited reports on the isolation of triterpenoid compounds from its stem bark. Therefore, this research attempted to isolate and elucidate seven triterpenoids belonging to dammarane-type (**1**–**7**) from the stem bark of *Aglaia cucullata.* The isolated compounds included 20*S*,24*S*-epoxy-3α,25-dihydroxy-dammarane (**1**), dammaradienone (**2**), 20*S*-hydroxy-dammar-24-en-3-on (**3**), eichlerianic acid (**4**), (20*S*,24*RS*)-23,24-epoxy-24-methoxy-25,26,27-tris-nor dammar-3-one (**5**), 3α-acetyl-cabraleahydroxy lactone (**6**), and 3α-acetyl-20*S*,24*S*-epoxy-3α,25-dihydroxydammarane (**7**). Employing spectroscopic techniques, the chemical structures of the triterpenoids were identified using FTIR, NMR, and HRESITOF-MS. The cytotoxic activity of compounds **1**–**7** was tested with the PrestoBlue cell viability reagent against MCF-7 breast cancer, B16-F10 melanoma, and CV-1 normal kidney fibroblast cell lines. The results displayed that compound **5** had the highest level of bioactivity compared to the others. Furthermore, the IC_50_ values obtained were more than 100 μM, indicating the low potential of natural dammarane-type triterpenoids as anticancer agents. These findings provided opportunities for further studies aiming to increase their cytotoxic activities through semi-synthetic methods.

## 1. Introduction

The Meliaceae family comprises flowering trees or shrubs that are commonly found in tropical and subtropical regions of Asia, Africa, and America [1]. Among the members of this family, the *Aglaia genus* has been reported to have the highest number of species, with more than 150 [2]. Furthermore, the *Aglaia* plant is primarily distributed in tropical and subtropical areas of Asia, Northern Australia, and the Pacific, with Indonesia being home to more than 65 species [2,3]. This plant has, over time, been implemented by Indonesians as traditional medication for wounds, fever, and skin disease [4]. Based on previous reports, the therapeutic effects exhibited can be attributed to their constituent secondary metabolites, such as diterpenoids [5,6], triterpenoids [7,8], sesquiterpenoids [9,10], limonoids [11,12], steroids [13], flavaglines [14], bisamides [15], and lignans [16,17]. Several studies showed that these metabolites had potential biological activity, including cytotoxic flavaglines [18,19], insecticidal rocaglamides [20], anti-inflammatory diterpenoids and triterpenoids [6], antifungal flavaglines [21], and molluscicide activity of triterpenoids [22].

Triterpenoids are a class of terpenoid compounds composed of six isoprene units and are biochemically synthesized through the mevalonate pathways in the endoplasmic reticulum and cytosol of plant cells [23,24]. Furthermore, approximately 20,000 triterpenoids have been isolated with 200 different skeletons [23], making them one of the most diverse chemical structures in terpenoid groups. These compounds and their derivatives have demonstrated remarkable biological activities, such as antiviral [25], antifungal [26], antibacterial [27], antioxidant [28], anti-inflammation [29], antidiabetic [30], and cytotoxic effects [31,32].

The *Aglaia* genus is known for its ability to produce different dammarane-type triterpenoids, with approximately 31 new compounds having been isolated [2,31,32]. Additionally, Zhang et al. [33] identified six novel compounds that demonstrated remarkable cytotoxic activity against various cancer cells. In a previous study, triterpenoids belonging to the dammarane type from *A. smithii* and *A. eximia* were found to exhibit cytotoxicity against P388 murine leukemia cells [7,34]. Furthermore, those obtained from *A. elaeagnoidea* had effects on cervical cancer (HeLa) and human prostate cancer (DU145) cells [35], and others from *A. elliptica* [31,32] showed cytotoxic effects against breast cancer (MCF-7) and melanoma (B16–F10) cells.

*Aglaia cucullata*, commonly known as Pacific maple, is a plant that thrives in mangrove areas and is the only member found in the coastal forest from South Asia [36,37,38,39]. In Indonesia, this plant is spread across various islands, such as Sumatera, Kalimantan, Sulawesi, Maluku, and Papua [37]. It has also been traditionally used for boat materials, house structures, and firewood [38]. Furthermore, certain communities utilize this plant to treat diarrhea, anti-inflammation, dysentery, rheumatism, skin infections, and heart problems [40,41]. Previous studies on its chemical composition revealed the presence of cytotoxic flavaglines, which acted against several human cancer lines, such as oral KB, breast cancer BC, and lung NCI-H187 [40]. The presence of bisamides [42], kaurene and labdane diterpenoids, and cycloartane triterpenoid with TRAIL resistance-overcoming activity has also been reported [38].

This research aims to isolate triterpenoid compounds from the stem bark of *A. cucullata* using extraction, separation, and purification methods such as maceration, partitioned, and column chromatography and identifying its chemical structure using various spectroscopic methods to give seven known dammarane triterpenoids (**1**–**7**). Furthermore, isolated compounds were examined for cytotoxic potency against MCF-7 breast cancer, B16-F10 melanoma, and CV-1 normal kidney fibroblast cell lines. A brief structure–activity relationship explanation of compounds **1**–**7** against these cell lines was also provided.

Based on the results, the isolation and structural identification of seven dammarane triterpenoids provided new information concerning the chemical constituent of *A. cucullata*. The examination of cytotoxicity against two cancer cell lines and one normal cell line provided insight into the activity of dammarane-type triterpenoids and their possibilities as anticancer agents.

## 2. Results and Discussions

### 2.1. Structural Identification of the Isolated Compounds

**Figure 1:**
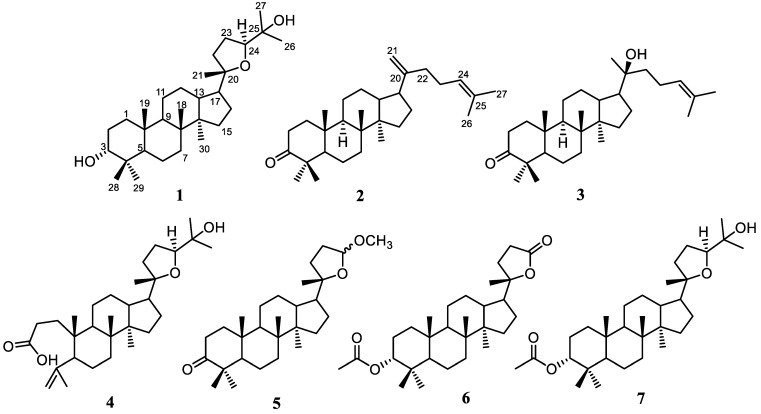
Structure of compounds **1**–**7**.

A total of 3.5 kg of dried crushed stembark of *A. cucullata* was macerated using EtOH and evaporated when the pressure was lower to produce 525 g of thick brown EtOH extract. The extract was dissolved in water and segmented based on differences in polarity, leading to the production of 64 g of *n*-hexane, 35 g of EtOAc, and 13 g of *n*-BuOH extracts. The *n*-hexane sample was then subjected to the Liebermann–Burchard test, which showed intense positive results, indicating the presence of triterpenoid compounds. Therefore, the process of chemical separation and purification was carried out on *n*-hexane extract. Vacuum liquid chromatography (VLC) was carried out along with silica gel 60-column chromatography, and the reverse phase ODS yielded seven triterpenoids belonging to dammarane-type **1**–**7** (Figure 1). All isolated compounds obtained in pure form were proofed by the TLC profile of each compound (Figure S59). However, after a detailed analysis of NMR followed by a comparison with the literature, the purity of compounds **1**–**7** is reliable, and no mixture is observed. The structure identification of the isolated dammarane triterpenoids was discussed based on spectroscopic data.

**Figure 2:**
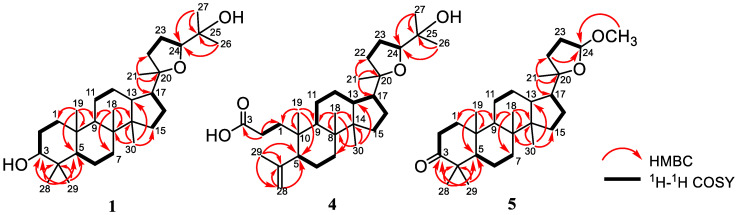
Selected HMBC and ^1^H–^1^H COSY correlations for **1**, **4**, and **5**.

Compound **1**, 20*S*,24*S*-epoxy-3α,25-dihydroxy-dammarane (**1**), was obtained as a white amorphous powder. The molecular composition of compound **1** according to HR-ESI-TOFMS was C_30_H_52_O_3_ (*m/z* 461.3993 [M+H]^+^ calculated for C_30_H_53_O_3_^+^, *m/z* 461.3995). Further analysis was carried out using NMR data, and the results were presented in Table 1 and Table 2. Furthermore, the FTIR spectrum showed the appearance of hydroxyl (3457 cm^−1^), *gem*-dimethyl (1380 cm^−1^), and ether groups (1055 cm^−1^). The ^13^C NMR (Table 1), along with the DEPT 135° and ^1^H-NMR spectra (Table 2), demonstrated that the NMR data of **1** had high similarity with the 20*S*,24*S*-epoxy-3α,25-dihydroxy-dammarane isolated from *A. elaeagnoidea* [35]. A 2D NMR spectrum of this compound (Figure 2) was also obtained to determine the exact structure of compound **1**. According to the evaluation, the structure was identified as a dammarane-type triterpenoid, particularly 20*S*,24*S*-epoxy-3α,25-dihydroxy-dammarane, which was isolated for the first time from *A. cucullata*.

Dammaradienon (**2**) was observed as a white amorphous, and its composition according to HR-ESI-TOFMS was identified as C_30_H_48_O (*m/z* 425.3781 [M+H]^+^ calculated for C_30_H_49_O^+^, *m/z* 425.3783) along with NMR data (Table 1 and Table 2). The FTIR spectrum indicated the appearance of olefinic (3081 cm^−1^ for stretching of the C-H *sp^2^* group and 1641 cm^−1^ for the C=C group), *gem*-dimethyl (1375 cm^−1^) and carbonyl (1705 cm^−1^) groups. The ^13^C NMR (Table 1), as well as the DEPT 135° and ^1^H-NMR spectra (Table 2), demonstrated that the NMR data of compound **2** were identical with the dammaradienone isolated from *Chisocheton pendoliflorus* [43]. Therefore, compound **2** was elucidated as a dammarane-type triterpenoid, namely dammaradienone, which was isolated from *A. cucullata* for the first time.

Compound **3**, 20*S*-hydroxy-dammar-24-en-3-on, was obtained as a white amorphous, and its molecular composition according to HR-ESI-TOFMS and NMR data was identified as C_30_H_50_O_2_ (*m/z* 443.3817 [M+H]^+^ calculated for C_30_H_51_O_2_^+^, *m/z* 443.3811), as presented in Table 1 and Table 2. The FTIR spectrum showed the appearance of a hydroxyl (3448 cm^−1^), a *gem*-dimethyl (1378 cm^−1^), and a carbonyl (1704 cm^−1^) group. The ^13^C NMR (Table 1), DEPT 135°, and ^1^H-NMR spectra (Table 2) showed that the NMR data of compound **3** had high similarity with 20*S*-hydroxy-dammar-24-en-3-on isolated from *A. elliptica* [44]. Consequently, the structure of **3** was identified as a dammarane triterpenoid, namely 20*S*-hydroxy-dammar-24-en-3-on, which was isolated from *A. cucullata* for the first time.

Eichlerianic acid (**4**) was observed as a white amorphous, and its composition according to HR-ESI-TOFMS and NMR data was C_30_H_50_O_4_ (*m/z* 475.3793 [M+H]^+^ calculated for C_30_H_51_O_4_^+^, *m/z* 475.3782) (Table 1 and Table 2). The FTIR spectrum showed the appearance of hydroxyl (3421 cm^−1^), *gem*-dimethyl (1376 cm^−1^), carbonyl (1704 cm^−1^), and ether (1078 cm^−1^) groups. The ^13^C NMR (Table 1), DEPT 135°, and ^1^H-NMR spectra (Table 2) presented that the NMR data of compound **4** were identical with eichlerianic acid isolated from *A. foveolata* [45]. Two-dimensional NMR spectra of this compound (Figure 2) were also obtained to determine its exact structure. Therefore, the structure was identified as a dammarane-type triterpenoid, namely eichlerianic acid, which was isolated from *A. cucullata* for the first time.

Compound **5**, (20*S*,24*RS*)-23,24-epoxy-24-methoxy-25,26,27-tris-nor dammar-3-one was observed as a white amorphous, and its composition according to HR-ESI-TOFMS and NMR data was C_28_H_46_O_3_ (*m/z* 453.3349 [M+Na]^+^ calculated for C_28_H_46_O_3_Na^+^, *m/z* 453.3345) (Table 1 and Table 3). The FTIR spectrum presented the appearance of *gem*-dimethyl (1377 cm^−1^), carbonyl (1703 cm^−1^), and ether (1083 cm^−1^) groups. The ^13^C NMR (Table 1) and DEPT 135° spectra gave the resonance of 28 carbon signals, which were classified into seven methyls (including one methoxy at δ_C_ 54.5), ten methylenes, five methines (consisting of one acetal at δ_C_ 104.7), and six quaternary carbons (including one oxygenated quaternary carbon at δ_C_ 88.1 and one ketone at δ_C_ 218.4). The functionalities of one of the six levels of unsaturation and the leftover five levels of unsaturation were suitable with a tetracyclic dammarane-like triterpenoid core with an additional epoxy ring on the side chain. The ^1^H-NMR spectrum (Table 3) showed seven tertiary methyls (δ_H_ 0.87, 0.92, 0.99, 1.03, 1.07, 1.12, and 3.30; each 3H) and one acetal proton at δ_H_ 4.91 (1H, br.s). The position of each functional group was determined by HMBC and ^1^H-^1^H COSY spectra. Furthermore, ketone was formed in C-3, as shown by the HMBC correlation of H-28 and H-29 to C-3, C-4, and C-5. HMBC correlations of H-1′ to C-24 were used to determine the exact position of the methoxy group at C-24. The ^1^H-^1^H COSY cross-peaks between H-22/H-23/H-24 provided information about the location of the acetal group at C-24, as well as the formation of an epoxide ring at C-20/C-24. An NMR data ratio of compound **5** with (20*S*,24*RS*)-23,24-epoxy-24-methoxy-25,26,27-tris-nordammar-3-one obtained from the oxidation product of dipterocarpol through chemical synthesis [46,47] showed that the two compounds were identical. Furthermore, this was the first report on the isolation and detailed assignment of NMR data of this compound from a natural source, which was often previously obtained from synthetic products. According to these findings, the structure of **5** was identified as a dammarane triterpenoid, namely (20*S*,24*RS*)-23,24-epoxy-24-methoxy-25,26,27-tris-nor dammar-3-one.

Compound **6**, 3α-acetyl-cabraleahydroxy lactone, was obtained as a white amorphous, and its molecular composition according to HR-ESI-TOFMS as well as NMR data was C_29_H_46_O_4_ (*m/z* 481.3284 [M+Na]^+^ calculated for C_29_H_46_O_4_Na^+^, *m/z* 481.3294), as presented in Table 1 and Table 2. The FTIR spectrum presented the appearance of a *gem*-dimethyl (1387 cm^−1^) and a carbonyl group (1715 cm^−1^). The ^13^C NMR (Table 1), DEPT 135°, and ^1^H-NMR spectra (Table 3) demonstrated that the NMR data of compound **6** had high similarity with cabraleahydroxy lactone isolated from *A. elaeagnoidea* [35]. The sole distinction was the addition of an acetyl group at compound **6**, which was identified based on the addition of two carbon atoms. Therefore, its structure was identified as a dammarane-type triterpenoid, namely 3α-acetyl-cabraleahydroxy lactone, which was isolated for the first time from *A. cucullata*.

3α-acetyl-20*S*,24*S*-epoxy-3α,25-dihydroxydammarane (**7**) was observed as a white amorphous, and its molecular composition according to HR-ESI-TOFMS and NMR data was C_32_H_54_O_4_ (*m/z* 525.3915 [M+Na]^+^ calculated for C_32_H_54_O_4_Na^+^, *m/z* 525.3914) (Table 1 and Table 3). The FTIR spectrum presented the appearance of hydroxyl (3403 cm^−1^), *gem*-dimethyl (1377 cm^−1^), and carbonyl (1701 cm^−1^) groups. The ^13^C NMR (Table 1), DEPT 135°, and ^1^H-NMR spectra (Table 3) demonstrated that the NMR data of compound **7** had high similarity with 3α-acetyl-20*S*,24*S*-epoxy-3α,25-dihydroxydammarane obtained from *A. elliptica* [13]. Therefore, its structure was identified as a dammarane-type triterpenoid, namely 3α-acetyl-20*S*,24*S*-epoxy-3α,25-dihydroxydammarane, which was fisolated from *A. cucullata* for the first time.

Compounds **1**–**7** were classified as dammarane-type triterpenoids. Although those were known compounds, compounds **1**–**7** were isolated from *A. cucullata* for the first time. This indicated that the species, along with other members of the *Aglaia* genus, could be used as a source of triterpenoid compounds. Dammarane-type triterpenoids were known for their biological activity, including cytotoxicity. Therefore, this study was consistent with previous reports where this plant was identified as a source of bioactive compounds [38,40,42].

### 2.2. Cytotoxic Activity Compounds ***1**–**7***

A normal cell (CV-1 kidney fibroblast cells) and two cancer cell lines (MCF-7 breast cancer and B16-F10 melanoma cells) were used to test the cytotoxic activities of compounds **1**–**7**. Furthermore, cisplatin (positive control) was used during the assessment, as presented in Table 4. In this experiment, two wavelengths (570 and 600 nm) were used because the measured cells were live cells so, to obtain the value/number of live cells, measurements were carried out at two wavelengths, namely before the reaction (blue) and after the reaction (pink) to produce a corrected absorbance value, which was interpreted as the number live cells after treatment [48]. The results arising from the experiments, along with IC_50_ calculation graphs and cell morphology at each sample concentration, are presented in Figures S38–S58.

In MCF-7 cells, compounds **2** and **5** showed cytotoxicity with an IC_50_ close to cisplatin due to its chemical structure. In compound **3**, the presence of the olefinic group at C-20 increased the cytotoxic activity compared with compound **4** with the hydroxyl group at C-20. A similar observation also occurred in compound **2**, where the additional olefinic group at C-20/C-21 suggested an increase in cytotoxic effect. Based on previous studies, the degradation of three carbon atoms to form an acetal group in the side chain, which was attached to a five-membered ring in compound **5**, was believed to increase cytotoxicity against cells [31].

Compounds **1**, **3**, and **5** showed similar cytotoxicity against B16-F10 melanoma cells compared to the positive control cisplatin. Compared to MCF-7, the appearance of the hydroxyl group at C-20 in compound **3** increased the activity against B16–F10 compared to compound **2** with the olefinic group at C-20. The results also demonstrated that compound **5** had better cytotoxicity against both cancer cells compared to the others. Furthermore, a significant difference was the existence of an acetal group in the side chain, suggesting that this group contributed to its bioactivity.

Among all isolated compounds, **4** showed the weakest cytotoxicity to both cancer cells, suggesting that its constituent ring A served as a negative factor. The compounds obtained in this study were also tested against CV-1 normal cell lines to determine their safety toward normal cells. Furthermore, **3**, **5**, and **7** exhibited high IC_50_ of >300 μg/mL (>300 μM), indicating that they were relatively safe. Based on morphological observation, there was no cell death at all concentration levels. 

The cytotoxicity assessment revealed that **5** was the most active compound in terms of cytotoxicity as opposed to MCF-7 and B16-F10, as well as the safest of the CV-1 normal cell lines. Furthermore, the IC_50_ range obtained for the natural dammarane-type triterpenoids was more than 100 μM, indicating that they were still far from being considered potential compounds for cytotoxic activity. A literature review by Cao et al. [49] stated that several herbal plants with high potential in treating various diseases, including cancer, contained dammarane-type triterpenoids. According to our results, dammarane-type triterpenoids alone give low cytotoxicity, suggesting that these compounds showed the synergistic effect with other active components in plant extracts to give a more significant effect. The semi-synthesis of dammarane-type triterpenoids also offered a pathway to obtain more active compounds through extensive modifications of their structure.

A review by Ruan et al. [50] explained that in addition to cytotoxic activity, dammarane triterpenoids also provided a broad spectrum of activity. Glycosylated dammarane triterpenoids showed potential anti-inflammation activity, whereas aglycon dammarane triterpenoids with double bonds in its side chain from *Panax ginseng* displayed immunomodulatory activity. Sapogenin-type dammarane triterpenoids performed remarkable antineoplastic activity. It was shown that glycosylated and additional double bonds in the side chain significantly increased biological activity. These phenomena give insightful thought that our compounds can be used after small functional group modifications to give desired activity.

## 3. Materials and Methods

### 3.1. General Experimental Procedures

High-resolution mass spectra (HRESI-TOFMS) were acquired using a Waters Xevo Q-TOF direct probe/MS system utilizing ESI+ mode with a microchannel plate MCP detector (Milford, MA, USA), and optical rotations were determined using an ATAGO AP-300 automated polarimeter (Saitama, Japan). Additionally, the One Perkin Elmer infrared-100 (Shelton, CT, USA) was used to measure the IR spectra. On a JEOL ECZ-500 spectrometer (Tokyo, Japan), the NMR data were collected for ^1^H (500 MHz) and ^13^C (125 MHz) using TMS as an internal standard. Then, chromatographic separations were carried out using octadecyl silane (Fuji Sylisia Chemical LTD., Chromatorex C18 DM1020 M, 200–400 mesh) and silica gel G60 (Merck, Darmstadt, Germany, 70–230 and 230–400 mesh). Precoating the TLC plates with GF254 (Merck, 0.25 mm) was followed by detection, which was carried out by spraying 10% H_2_SO4 in ethanol and then heating.

### 3.2. Plant Material

The stem bark of *A. cucullata* was derived from the Manggar River in Balikpapan, East Kalimantan, Indonesia. The sample was assessed by the Herbarium Wanariset staff, Balikapapan, in December 2020, and the specimen was deposited at the herbarium (collection No. FF7.20).

### 3.3. Extraction and Isolation

*A. cucullata*’s crushed dried stem bark (3.5 kg) was extracted with ethanol (5 × 3 L) over the course of 5 days at room temperature. A total of 523 g of the extract were collected after the solvent was evaporated, and it were separated into various polarities to produce 64 g *n*-hexane, 35 g ethyl acetate, and 13 g *n*-butanol. With the help of vacuum liquid chromatography, 64 g of the *n*-hexane extract was separated into eight fractions (A–H) using a gradient elution of *n*-hexane ethyl acetate methanol (10:0:0–0:0:10, stepwise 10%; v: 500 mL). A gradient elution of *n*-hexane ethyl acetate (10:0–0:10, stepwise 5%; v: 300 mL) was used to separate 21 g of C using the chromatographic procedure to create six fractions (B1-B6) on silica gel (200 g of G60 silica gel). Then, a gradient elution of *n*-hexane methylene chloride (10:0–1:1 stepwise 2.5%; r: 2 cm; h: 17 cm; v: 250 mL) was used to separate a total of 2.7g B1 into nine fractions (B1a-B1i) using silica gel column chromatography (70–230 mesh, 80 g). B1e (146 mg) was separated with silica gel column chromatography (230–400 mesh, 10 g) using *n*-hexane ethyl acetate, 50:1, to yield B1e1-B1e3. B1e2 (94 mg) was separated with ODS column chromatography (100–200 mesh, 2.5 g) using methanol/water in a ratio of 20:1 to produce compound **1** (10 mg). The process was continued by separating 183 mg of B1g with silica gel column chromatography (230–400 mesh, 15 g) using *n*-hexane/ethyl acetate in a ratio of 50:1 to yield B1g1-B1g2. Compound **2** (93 mg) was isolated by subjecting around 157 mg of B1g1 to ODS column chromatography (100–200 mesh, 7 g) with methanol/water in a 20:1 ratio. Eleven fractions (B2a–B2k) were obtained from 1.5 g of B2 using silica gel column chromatography (70–230 mesh, 50 g) and a gradient elution of n-hexane ethyl acetate (10:0–1:1 stepwise 2.5%; r: 2 cm; h: 17 cm; v: 200 mL). B2c1–B2c3 were produced from 248 mg of B2c using silica gel column chromatography (230–400 mesh, 22 g) in a 20:1 *n*-hexane/acetone ratio. Compound **3** (75 mg) was then produced by separating a total of 200 mg of B2c3 using an ODS column chromatography (100–200 mesh, 5 g) with methanol/water in a 20:1 ratio. Three fractions (B2d1–B2d3) were obtained after silica gel column chromatography (230–400 mesh, 25 g) was used to separate a total of 240 mg of fraction B2d. Compound **4** (92 mg) was produced by ODS column chromatography (100–200 mesh, 13 g) with a methanol/water ratio of 4:1 from 170 mg of B2d3. After that, 103 mg of B2e was separated using silica gel column chromatography (230–400 mesh, 13 g), using *n*-hexane/acetone in a 20:1 ratio to produce four fractions (B2e1–B2e4). Compound **5** (5 mg) was generated by employing ODS column chromatography (100–200 mesh, 1 g) with a methanol/water ratio of 10:1 to separate a total of 21 mg of B2e1. Additionally, seven fractions (B2h1–B2h7) were produced from 165 mg of B2h using ODS column chromatography (100–200 mesh, 12 g) in a methanol/water ratio of 10:1. Compound **6** (5 mg) was isolated by separating around 40 mg of B2h1 using ODS column chromatography (100–200 mesh, 4 g, methanol/water, 9:1). Fraction B2h3 from the previous process was then used to produce compound **7** (53 mg).

### 3.4. Determination of Cytotoxic Activity

The PrestoBlue assay was utilized to conduct the cytotoxic bioassay. Additionally, Presto Blue reagent from Thermo Fisher Scientific, Uppsala, Sweden, was used to measure cell viability, which allowed for rapid evaluation of various resazurin-based cells. The rate of proliferation of the samples was then determined quantitatively through live-cell reduction capabilities. According to the findings, healthy cells kept a reduced habitat in their cytoplasm. By reducing resorufin (purple) with absorbance or fluorescence outputs, resazurin reduction (blue) acted as a viability indicator. The conversion correlated with the number of cells that were metabolically active. MCF-7, B16-F10, and CV-1 cell lines were cultured until they reached 70% confluence, and then they were removed, counted with a hemocytometer, and diluted with complete culture RPMI media. A total of 170,000 cells per well in 96-well plates with the samples were used. After an overnight growth period, the samples were treated with compounds **1**–**7** at escalating concentrations (3.91, 7.81, 15.63, 31.25, 62.50, 125, 250, and 5000 μg/mL) in PBS, with 2% DMSO as the co-solvent. Additionally, cisplatin served as the positive control, and all samples were incubated for 24 h at 37° C with 5% CO_2_. Following incubation, the medium was immediately changed to 10 μL of the PrestoBlue reagent in 90 μL of the RPMI medium. As resorufin began to form, the color of the plates changed from blue to purple after being incubated for another one to two hours. Using a microplate reader to measure absorbance at 570 nm and 600 nm, the concentration required to inhibit growth by 50% was calculated as the IC_50_ value. The concentration was calculated using a plot of cytotoxicity against sample concentrations, and the result revealed 50% cytotoxicity (IC_50_). The results of each test and analysis were averaged after being run twice.

## 4. Conclusions

In this study, seven dammarane-type triterpenoids (**1**–**7**) were successively isolated for the first time from the stembark of *A. cucullata* using various extraction and chromatography methods. Furthermore, their chemical structures were unambiguously determined using various spectroscopic methods. All isolated compounds were tested utilizing PrestoBlue reagent toward MCF-7 breast cancer, B16-F10 melanoma, and CV-1 normal kidney fibroblast cell lines. Based on the results, compound **5** was found to be the most active compared to the others.

## Figures and Tables

**Table 1:** ^13^C NMR data (500 MHz for ^1^H and 125 MHz in CDCl_3_) for **1**–**7**.

Position	1	2	3	4	5	6	7
1	33.7	40	39.9	34.3	39.9	34.3	34.4
2	25.4	34.1	34.1	28.2	32.8	25.1	23
3	76.4	218.2	218	179.3	218.4	78.4	78.5
4	37.3	47.4	47.4	147.5	47.5	37.2	36.8
5	49.6	55.4	55.4	50.8	55.4	50.4	50.6
6	18.3	19.7	19.7	24.6	19.7	18.1	18.2
7	34.8	34.8	34.6	33.9	34.6	35.1	34.7
8	40.7	40.4	40.3	40	40.3	50.3	40.6
9	50.7	50.3	50	41.2	50.1	50.7	50.8
10	37.7	36.9	36.8	39.1	36.9	36.8	37.2
11	21.7	21.9	22	22.3	26.7	26.8	21.7
12	27.1	25	27.5	26.9	22	21.3	27.1
13	42.8	45.4	42.4	42.9	43.6	43.2	42.9
14	50.2	49.4	50.3	50.4	50.1	40.6	50.2
15	31.5	31.4	31.2	31.5	31.3	31.1	31.6
16	25.9	28.9	24.8	25.8	25.5	22.9	25.9
17	49.8	47.8	49.8	49.7	51.2	49.4	49.9
18	16.2	15.8	15.2	16.3	15.3	15.6	15.6
19	16.6	16.1	16	20.2	16.3	16	16.1
20	86.7	152.6	75.4	86.6	88.1	90.3	86.7
21	27.3	107.6	25.5	27.2	23.5	25.5	27.3
22	35.3	34.2	40.5	34.7	35.1	31.3	35.2
23	26.4	27.1	22.6	26.4	34.2	29.3	26.4
24	86.3	124.4	124.7	86.4	104.7	176.9	86.4
25	70.3	131.5	131.6	70.3	-	-	70.3
26	27.9	25.7	25.7	27.9	-	-	27.9
27	24.1	17.7	17.7	23.2	-	-	24.1
28	28.4	26.8	26.7	113.5	26.8	27.9	27.8
29	22.2	21	21	24	21.1	21.8	21.8
30	15.6	15.4	16.4	15.3	16.1	16.5	16.7
1′					54.5	170.9	171
2′						21.5	21.5

**Table 2:** ^1^H NMR Data (500 MHz for ^1^H in CDCl_3_) for **1**–**4**.

Position	1	2	3	4
1	1.42 (2H, m)	1.42, 1.94 (each 1H, m)	1.86 (2H, m)	1.97, 1.53 (each 1H, m)
2	1.55 (2H, m)	2.42, 2.48 (each 1H, m)	2.37, 2.42 (each 1H, m)	2.39, 2.18 (each 1H, m)
3	3.38 (1H, t, 3)	-	-	-
4	-	-	-	-
5	1.24 (1H, m)	1.37 (1H, m)	1.32 (1H, m)	-
6	1.39 (2H, m)	1.35, 1.50 (each 1H, m)	1.49, 1.40 (each 1H, m)	1.55 (2H, m)
7	1.63 (2H, m)	1.31, 1.63 (each 1H, m)	1.50, 1.26 (each 1H, m)	1.35 (2H, m)
8	-	-	-	-
9	1.44 (1H, m)	1.40 (1H, m)	1.36 (1H, m)	1.45 (1H, m)
10	-	-	-	-
11	1.53 (2H, m)	1.40 (2H, m)	1.45, 1.25 (each 1H, m)	1.41 (2H, m)
12	1.75 (2H, m)	1.55, 1.84 (each 1H, m)	1.79, 1.23 (each 1H, m)	1.89 (2H, m)
13	1.62 (1H, m)	1.67 (1H, m)	1.60 (1H, m)	1.62 (1H, m)
14	-	-	-	-
15	1.04 (2H, m)	1.10, 1.57 (each 1H, m)	1.40, 1.03 (each 1H, m)	1.43 (2H, m)
16	1.51 (2H, m)	1.89 (2H, m)	1.69, 1.44 (each 1H, m)	1.78 (2H, m)
17	1.44 (1H, m)	2.18 (1H, m)	1.68 (1H, m)	1.81 (1H, m)
18	0.84 (3H, s)	0.86 (3H, s)	0.93 (3H, s)	0.89 (3H, s)
19	0.87 (3H, s)	0.92 (3H, s)	0.88 (3H, s)	0.86 (3H, s)
20	-	-	-	-
21	1.13 (3H, s)	4.69, 4.72 (each 1H, br.s)	1.08 (3H, s)	1.15 (3H, s)
22	1.22 (2H, m)	1.95 (2H, m)	1.42 (2H, m)	1.54 (2H, m)
23	1.85 (2H, m)	2.10 (2H, q, 7.5)	1.99 (2H, m)	1.86 (2H, m)
24	3.62 (1H, dd, 4.8, 10.2)	5.11 (1H, dd, 7.0, 1.5)	5.05 (1H, t, 5.0)	3.64 (1H, dd, 9.5, 5.5)
25	-	-	-	-
26	1.17 (3H, s)	1.60 (3H, s)	1.62 (3H, s)	1.20 (3H, s)
27	1.09 (3H, s)	1.67 (3H, s)	1.56 (3H, s)	1.12 (3H, s)
28	0.92 (3H, s)	1.02 (3H, s)	1.01 (3H, s)	4.66, 4.85 (each 1H, br.s)
29	0.82 (3H, s)	1.06 (3H, s)	0.97 (3H, s)	1.73 (3H, s)
30	0.95 (3H, s)	0.99 (3H, s)	0.82 (3H, s)	1.02 (3H, s)

**Table 3:** ^1^H NMR data (500 MHz for ^1^H in CDCl_3_) for **5**–**7**.

Position	5	6	7
1	1.92, 1.45 (each 1H, m)	1.31, 1.36 (each 1H, m)	1.40, 1.45 (each 1H, m)
2	1.87, 2.05 (each 1H, m)	1.57, 1.92 (each 1H, m)	1.61 (2H, m)
3	-	4.60 (1H, br.s)	4.61 (1H, t, 3.5)
4	-	-	-
5	1.36 (1H, m)	1.26 (1H, m)	1.43 (1H, m)
6	1.44, 1.55 (each 1H, m)	1.41 (1H, m)	1.37 (2H, m)
7	1.89, 1.54 (each 1H, m)	1.21, 1.52 (each 1H, m)	1.65, 1.85 (each 1H, m)
8	-	-	-
9	1.39 (1H, m)	1.42 (1H, m)	1.21 (1H, m)
10	-	-	-
11	1.79 (2H, m)	1.75, 1.19 (each 1H, m)	1.54 (2H, m)
12	1.23, 1.47 (each 1H, m)	1.13, 1.50 (each 1H, m)	1.78 (2H, m)
13	1.58 (1H, m)	1.55 (1H, m)	1.58 (1H, m)
14	-	-	-
15	1.46 (2H, m)	1.11, 1.48 (each 1H, m)	1.06, 1.42 (each 1H, m)
16	1.31 (each 1H, m)	1.39, 1.82 (each 1H, m)	1.85 (2H, m)
17	1.85 (1H, m)	1.98 (1H, m)	1.88 (1H, m)
18	0.99 (3H, s)	0.95 (3H, s)	0.96 (3H, s)
19	0.92 (3H, s)	0.84 (3H, s)	0.85 (3H, s)
20	-	-	-
21	1.12 (3H, s)	1.36 (3H, s)	1.14 (3H, s)
22	1.88, 1.82 (each 1H, m)	1.94 2.10 (each 1H, m)	1.24 (2H, m)
23	2.40, 2.50 (each 1H, m)	2.53 2.63 (each 1H, m)	1.75 (2H, m)
24	4.91 (1H, br.s)	-	3.63 (1H, dd, 4.7, 10.0)
25	-	-	-
26	-	-	1.18 (3H, s)
27	-	-	1.10 (3H, s)
28	1.07 (3H, s)	0.91 (3H, s)	0.82 (3H, s)
29	1.03 (3H, s)	0.82 (3H, s)	0.87 (3H, s)
30	0.87 (3H, s)	0.86 (3H, s)	0.91 (3H, s)
1′	3.30 (3H, s)	-	-
2′		2.07 (3H, s)	2.08 (3H, s)

**Table 4:** Cytotoxic activities against MCF-7, B16-F10, and CV-1 cell lines for **1**–**7**.

Compounds	IC_50_ for MCF-7 (μg/mL)	IC_50_ for MCF-7(μM)	IC_50_ for B16-F10 (μg/mL)	IC_50_ for B16-F10 (μM)	IC_50_ for CV-1 (μg/mL)	IC_50_ for CV-1 (μM)
20*S*,24*S*-epoxy-3α,25-dihydroxy-dammarane (**1**)	65.54	142.25	44.84	97.33	80.08	173.82
dammaradienone (**2**)	56.15	132.21	54.77	128.96	82.85	195,07
20*S*-hydroxy-dammar-24-en-3-on (**3**)	142.00	>300	49.08	110.86	>300	>300
eichlerianic acid (**4**)	>300	>300	275.40	>300	136.60	287.76
(20*S*,24*R,S*)-23,24-epoxy-24-methoxy-25,26,27-tris-nordammar-3-one (**5**)	57.34	133.13	45.36	105.32	>300	>300
3α-acetyl-cabraleahydroxy lactone (**6**)	125.89	274.45	125.89	274.45	130.70	285.00
3α-acetyl-20*S*,24*S*-epoxy-3α,25-dihydroxydammarane (**7**)	97.91	194.73	79.43	157.97	>300	>300
Cisplatin (positive control)	53.00	176.02	43.00	142.80	43.00	142.80

## Data Availability

All the data in this study are presented in the manuscript and Supplementary Materials.

## Supplementary Materials

The following supporting information can be downloaded at https://www.mdpi.com/article/10.3390/molecules28134946/s1, Figure S1. HRTOFMS Spectrum of **1**, Figure S2. FTIR Spectrum of **1**, Figure S3. 1H-NMR Spectrum of **1** (500 MHz in CDCl_3_), Figure S4. 13C-NMR and DEPT-135° Spectra of **1** (125 MHz in CDCl_3_), Figure S5. HMQC Spectrum of **1**, Figure S6. HMBC Spectrum of **1**, Figure S7. ^1^H-^1^H-COSY Spectrum of **1**, Figure S8. HRTOFMS Spectrum of **2**, Figure S9. FTIR Spectrum of **2**, Figure S10. 1H-NMR Spectrum of **2** (500 MHz in CDCl_3_), Figure S11. ^13^C-NMR and DEPT-135° Spectra of **2** (125 MHz in CDCl_3_), Figure S12. HRTOFMS Spectrum of **3**, Figure S13. FTIR Spectrum of **3**, Figure S14. ^1^H-NMR Spectrum of **3** (500 MHz in CDCl_3_), Figure S15. 13C-NMR and DEPT-135° Spectra of **3** (125 MHz in CDCl_3_), Figure S16. HRTOFMS Spectrum of **4**, Figure S17. FTIR Spectrum of **4**, Figure S18. ^1^H-NMR Spectrum of **4** (500 MHz in CDCl_3_), Figure S19. 13C-NMR and DEPT-135° Spectra of **4** (125 MHz in CDCl_3_), Figure S20. HMQC Spectrum of **4**, Figure S21. HMBC Spectrum of **4**, Figure S22. ^1^H-^1^H-COSY Spectrum of **4**, Figure S23. HRTOFMS Spectrum of **5**, Figure S24. FTIR Spectrum of **5**, Figure S25. ^1^H-NMR Spectrum of **5** (500 MHz in CDCl_3_), Figure S26. ^13^C-NMR and DEPT-135° Spectra of **5** (125 MHz in CDCl_3_), Figure S27. HMQC Spectrum of **5**, Figure S28. HMBC Spectrum of **5**, Figure S29. ^1^H-^1^H-COSY Spectrum of **5**, Figure S30. HRTOFMS Spectrum of **6**, Figure S31. FTIR Spectrum of **6**, Figure S32. ^1^H-NMR Spectrum of **6** (500 MHz in CDCl_3_), Figure S33. ^13^C-NMR and DEPT-135° Spectra of **6** (125 MHz in CDCl3), Figure S34. HRTOFMS Spectrum of **7**, Figure S35. FTIR Spectrum of **7**, Figure S36. 1H-NMR Spectrum of **7** (500 MHz in CDCl_3_), Figure S37. ^13^C-NMR and DEPT-135° Spectra of **7** (125 MHz in CDCl_3_), Figure S38. Results of cytotoxic activity of **1** against the MCF-7 cell line, Figure S39. Results of cytotoxic activity of **2** against the MCF-7 cell line, Figure S40. Results of cytotoxic activity of **3** against the MCF-7 cell line, Figure S41. Results of cytotoxic activity of **4** against the MCF-7 cell line, Figure S42. Results of cytotoxic activity of **5** against the MCF-7 cell line, Figure S43. Results of cytotoxic activity of **6** against the MCF-7 cell line, Figure S44. Results of cytotoxic activity of **7** against the MCF-7 cell line, Figure S45. Results of cytotoxic activity of **1** against the B16-F10 cell line, Figure S46. Results of cytotoxic activity of **2** against the B16-F10 cell line, Figure S47. Results of cytotoxic activity of **3** against the B16-F10 cell line, Figure S48. Results of cytotoxic activity of **4** against the B16-F10 cell line, Figure S49. Results of cytotoxic activity of **5** against the B16-F10 cell line, Figure S50. Results of cytotoxic activity of **6** against the B16-F10 cell line, Figure S51. Results of cytotoxic activity of **7** against the B16-F10 cell line, Figure S52. Results of cytotoxic activity of **1** against the CV-1 cell line, Figure S53. Results of cytotoxic activity of **2** against the CV-1 cell line, Figure S54. Results of cytotoxic activity of **3** against the CV-1 cell line, Figure S55. Results of cytotoxic activity of **4** against the CV-1 cell line, Figure S56. Results of cytotoxic activity of **5** against the CV-1 cell line, Figure S57. Results of cytotoxic activity of **6** against the CV-1 cell line, Figure S58. Results of cytotoxic activity of **7** against the CV-1 cell line. Figure S59. TLC profile of compounds **1**–**7**.

## Sample Availability

Samples of compounds **1**–**7** are available from the authors.
