# Supplementary material for: The Cytotoxic Activity of Dammarane-Type Triterpenoids Isolated from the Stem Bark of Aglaia cucullata (Meliaceae)
Source: Molecules. 2023 Jun 23;28(13):4946. doi: 10.3390/molecules28134946 (PMC10343279; doi:10.3390/molecules28134946)

Supplementary Material

Cytotoxic Activity of Dammarane-Type Triterpenoids from the Stem Bark of *Aglaia cucullata* (Meliaceae)

Purnama [1], Kindi Farabi [1,3], Dudi Runadi [1], Hadi Kuncoro [2], Desi Harneti [1], Nurlelasari [1], Tri Mayanti [1], Mohamad Nurul Azmi [4], Sofa Fajriah [5], Unang Supratman [1,3],*

[1]Department of Chemistry, Faculty of Mathematics and Natural Sciences, Universitas Padjadjaran, Jatinangor, 45363, Indonesia

[2]Faculty of Pharmacy, Universitas Mulawarman, Samarinda, 75123, East Kalimantan, Indonesia

[3]Central Laboratory, Universitas Padjadjaran, Jatinangor, 45363, Indonesia.

[4]School of Chemical Sciences, Universiti Sains Malaysia, 11800 Minden, Penang, Malaysia

[5]Research Center for Pharmaceutical Ingredients and Traditional Medicine, National Research and Innovation Agency (BRIN), Complex Cibinong Science Center – BRIN., Cibinong 16911, Bogor, Jawa Barat, Indonesia

* Correspondence: unang.supratman@unpad.ac.id; Tel.: +62 22 7794391

Contents

Figure S1. HRTOFMS Spectrum of **1**.

Figure S2. FTIR Spectrum of **1**.

Figure S3. ^{1}H-NMR Spectrum of **1** (500 MHz in $CDCl_3$).

Figure S4. ^{13}C-NMR and DEPT-135° Spectrum of **1** (125 MHz in $CDCl_3$).

Figure S5. HMQC Spectrum of **1**.

Figure S6. HMBC Spectrum of **1**.

Figure S7. ^{1}H-^{1}H-COSY Spectrum of **1**.

Figure S8. HRTOFMS Spectrum of **2**.

Figure S9. FTIR Spectrum of **2**.

Figure S10. ^{1}H-NMR Spectrum of **2** (500 MHz in $CDCl_3$).

Figure S11. ^{13}C-NMR and DEPT-135° Spectrum of **2** (125 MHz in $CDCl_3$).

Figure S12. HRTOFMS Spectrum of **3**.

Figure S13. FTIR Spectrum of **3**.

Figure S14. ^{1}H-NMR Spectrum of **3** (500 MHz in $CDCl_3$).

Figure S15. ^{13}C-NMR and DEPT-135° Spectrum of **3** (125 MHz in $CDCl_3$).

Figure S16. HRTOFMS Spectrum of **4**.

Figure S17. FTIR Spectrum of **4**.

Figure S18. ^{1}H-NMR Spectrum of **4** (500 MHz in $CDCl_3$).

Figure S19. ^{13}C-NMR and DEPT-135° Spectrum of **4** (125 MHz in $CDCl_3$).

Figure S20. HMQC Spectrum of **4**.

Figure S21. HMBC Spectrum of **4**.

Figure S22. ^{1}H-^{1}H-COSY Spectrum of **4**.

Figure S23. HRTOFMS Spectrum of **5**.

Figure S24. FTIR Spectrum of **5**.

Figure S25. ^{1}H-NMR Spectrum of **5** (500 MHz in $CDCl_3$).

Figure S26. ^{13}C-NMR and DEPT-135° Spectrum of **5** (125 MHz in $CDCl_3$).

Figure S27. HMQC Spectrum of **5**.

Figure S28. HMBC Spectrum of **5**.

Figure S29. ^{1}H-^{1}H-COSY Spectrum of **5**.

Figure S30. HRTOFMS Spectrum of **6**.

Figure S31. FTIR Spectrum of **6**.

Figure S32. ^{1}H-NMR Spectrum of **6** (500 MHz in $CDCl_3$).

Figure S33. ^{13}C-NMR and DEPT-135° Spectrum of **6** (125 MHz in $CDCl_3$).

Figure S34. HRTOFMS Spectrum of **7**.

Figure S35. FTIR Spectrum of **7**.

Figure S36. ^{1}H-NMR Spectrum of **7** (500 MHz in $CDCl_3$).

Figure S37. ^{13}C-NMR and DEPT-135° Spectrum of **7** (125 MHz in $CDCl_3$).

Figure S38. Results of cytotoxic activity of **1** against MCF-7 cell line.

Figure S39. Results of cytotoxic activity of **2** against MCF-7 cell line.

Figure S40. Results of cytotoxic activity of **3** against MCF-7 cell line.

Figure S41. Results of cytotoxic activity of **4** against MCF-7 cell line.

Figure S42. Results of cytotoxic activity of **5** against MCF-7 cell line.

Figure S43. Results of cytotoxic activity of **6** against MCF-7 cell line.

Figure S44. Results of cytotoxic activity of **7** against MCF-7 cell line.

Figure S45. Results of cytotoxic activity of **1** against B16-F10 cell line.

Figure S46. Results of cytotoxic activity of **2** against B16-F10 cell line.

Figure S47. Results of cytotoxic activity of **3** against B16-F10 cell line.

Figure S48. Results of cytotoxic activity of **4** against B16-F10 cell line.

Figure S49. Results of cytotoxic activity of **5** against B16-F10 cell line.

Figure S50. Results of cytotoxic activity of **6** against B16-F10 cell line.

Figure S51. Results of cytotoxic activity of **7** against B16-F10 cell line.

Figure S52. Results of cytotoxic activity of **1** against CV-1 cell line.

Figure S53. Results of cytotoxic activity of **2** against CV-1 cell line.

Figure S54. Results of cytotoxic activity of **3** against CV-1 cell line.

Figure S55. Results of cytotoxic activity of **4** against CV-1 cell line.

Figure S56. Results of cytotoxic activity of **5** against CV-1 cell line.

Figure S57. Results of cytotoxic activity of **6** against CV-1 cell line.

Figure S58. Results of cytotoxic activity of **7** against CV-1 cell line.

Figure S59. TLC profile of compounds **1-7**.

Figure S1. HRTOFMS Spectrum of **1**.

Single Mass Analysis
Tolerance = 5.0 mDa / DBE: min = -1.5, max = 50.0
Element prediction: Off
Number of isotope peaks used for i-FIT = 3

Monoisotopic Mass, Even Electron Ions
109 formula(e) evaluated with 1 results within limits (all results (up to 1000) for each mass)
Elements Used:
C: 0-500 H: 0-1000 O: 0-200
DAMMARANE KINDI 4 (0.085) Cm (2:5)
TOF MS ES+

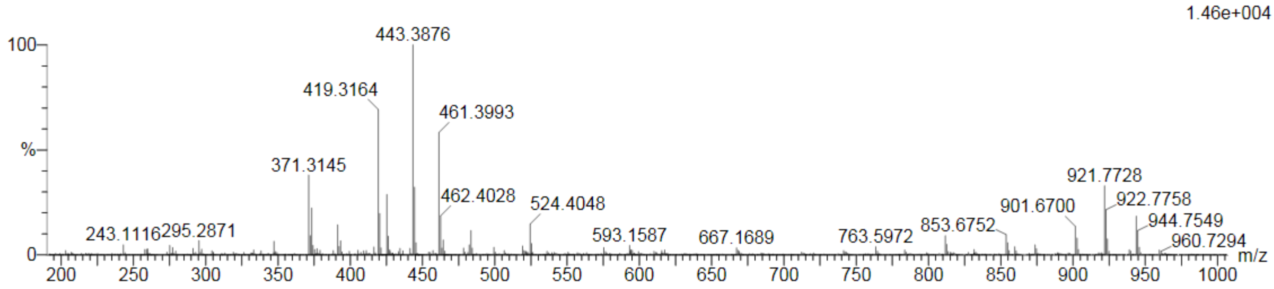

Minimum:			-1.5	
Maximum:	5.0	10.0	50.0	

Mass	Calc. Mass	mDa	PPM	DBE	i-FIT	i-FIT (Norm)	Formula
461.3993	461.3995	-0.2	-0.4	4.5	93.2	0.0	C30 H53 O3

Figure S2. FTIR Spectrum of **1**.

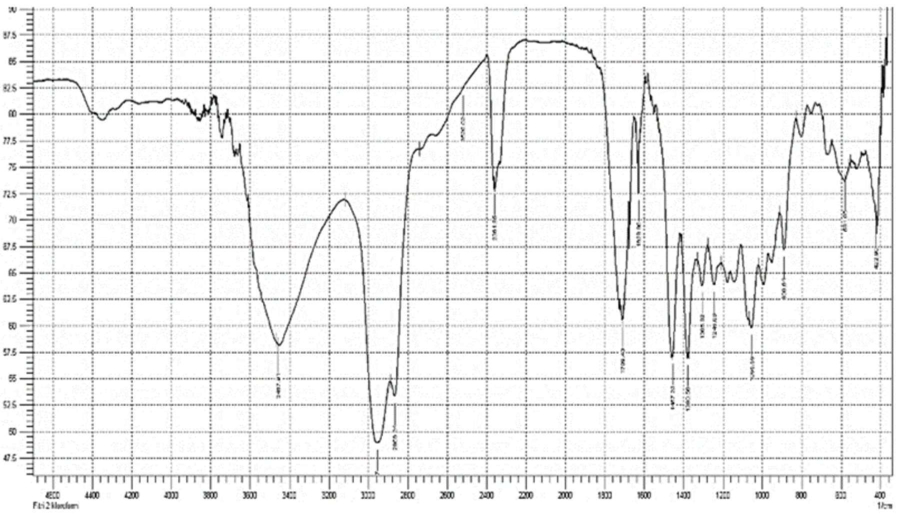

Figure S3. ^{1}H-NMR Spectrum of **1** (500 MHz in $CDCl_3$).

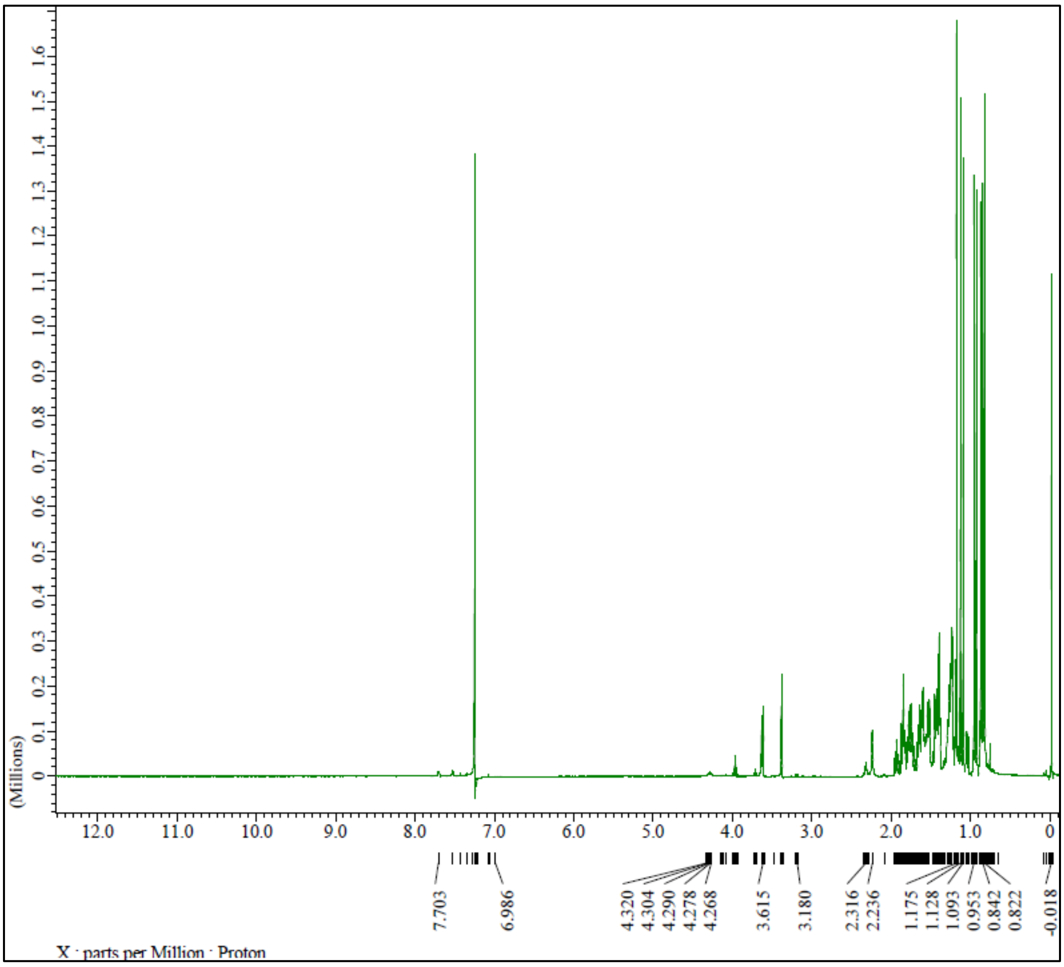

Figure S4. ^{13}C-NMR and DEPT-135° Spectrum of **1** (125 MHz in $CDCl_3$).

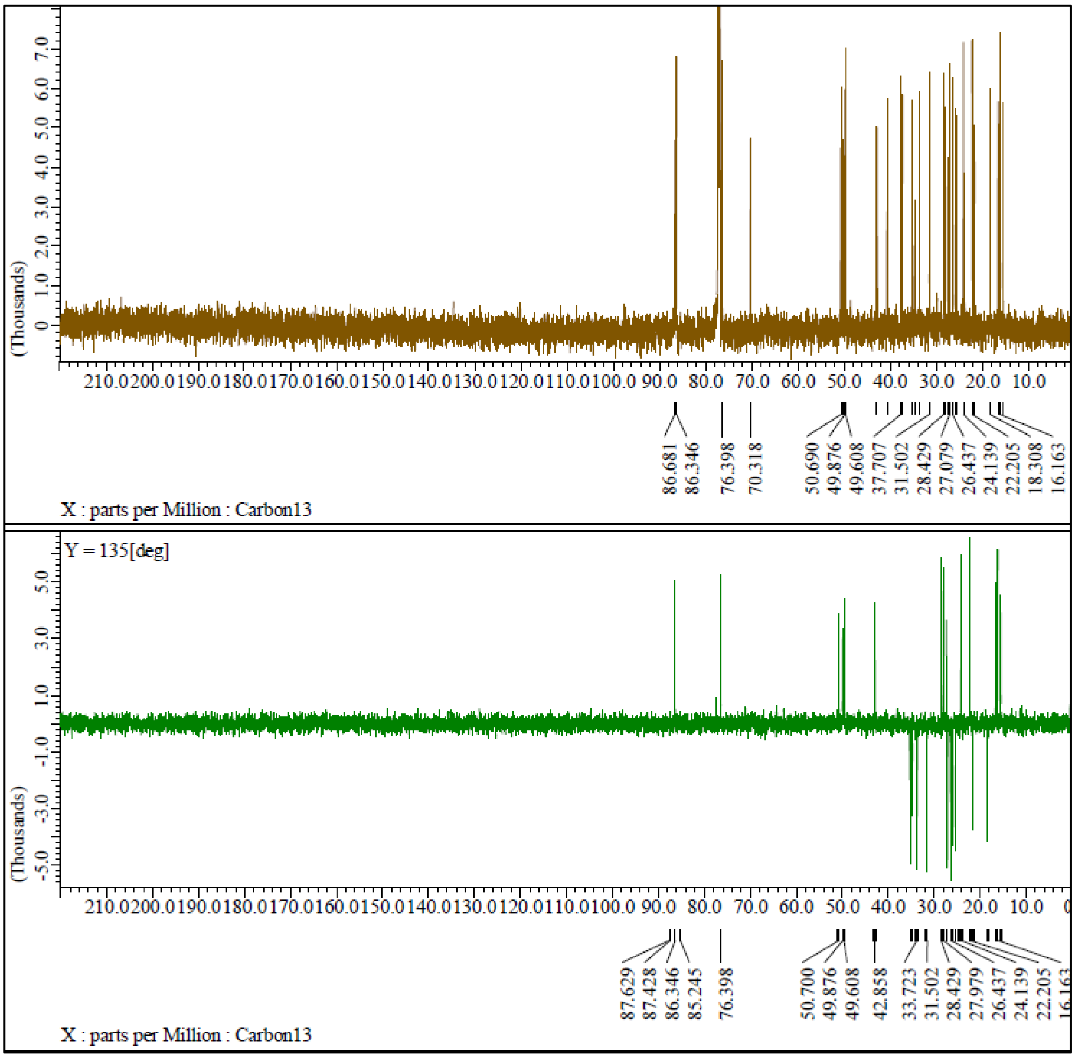

Figure S5. HMQC Spectrum of **1**.

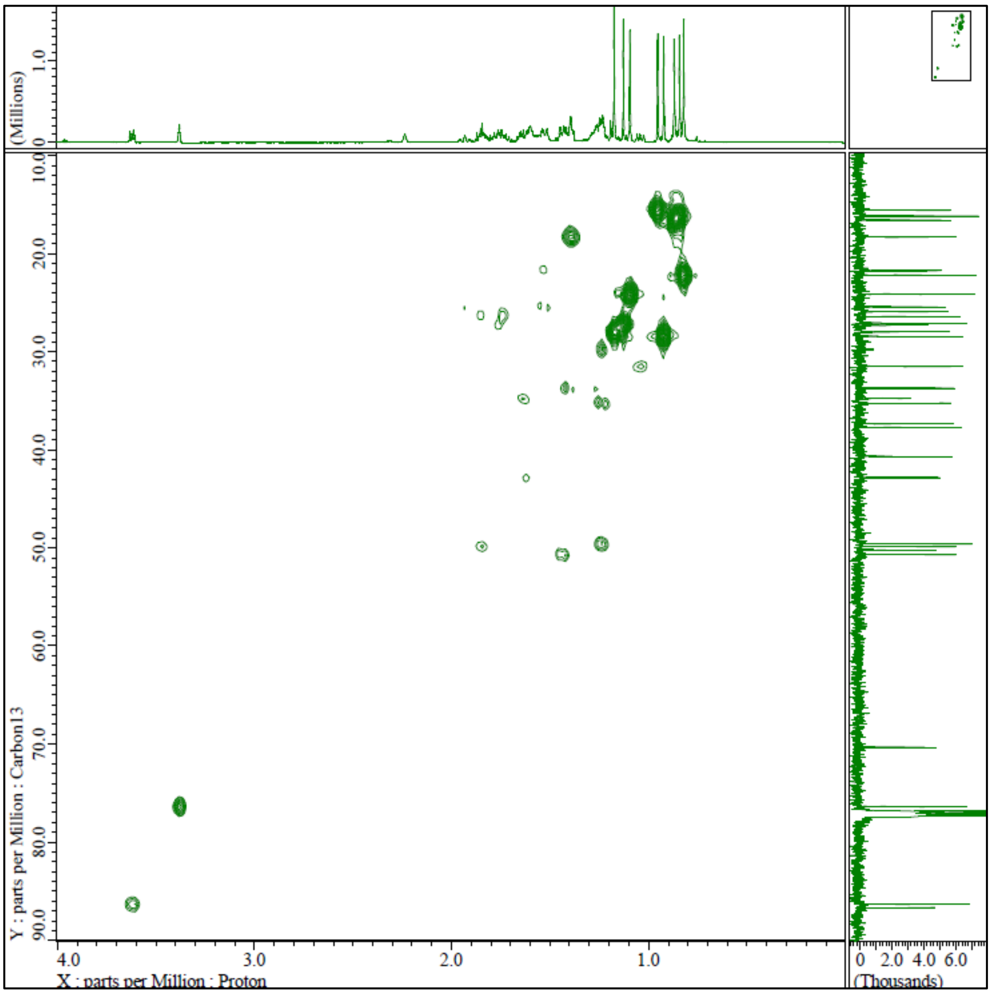

Figure S6. HMBC Spectrum of **1**.

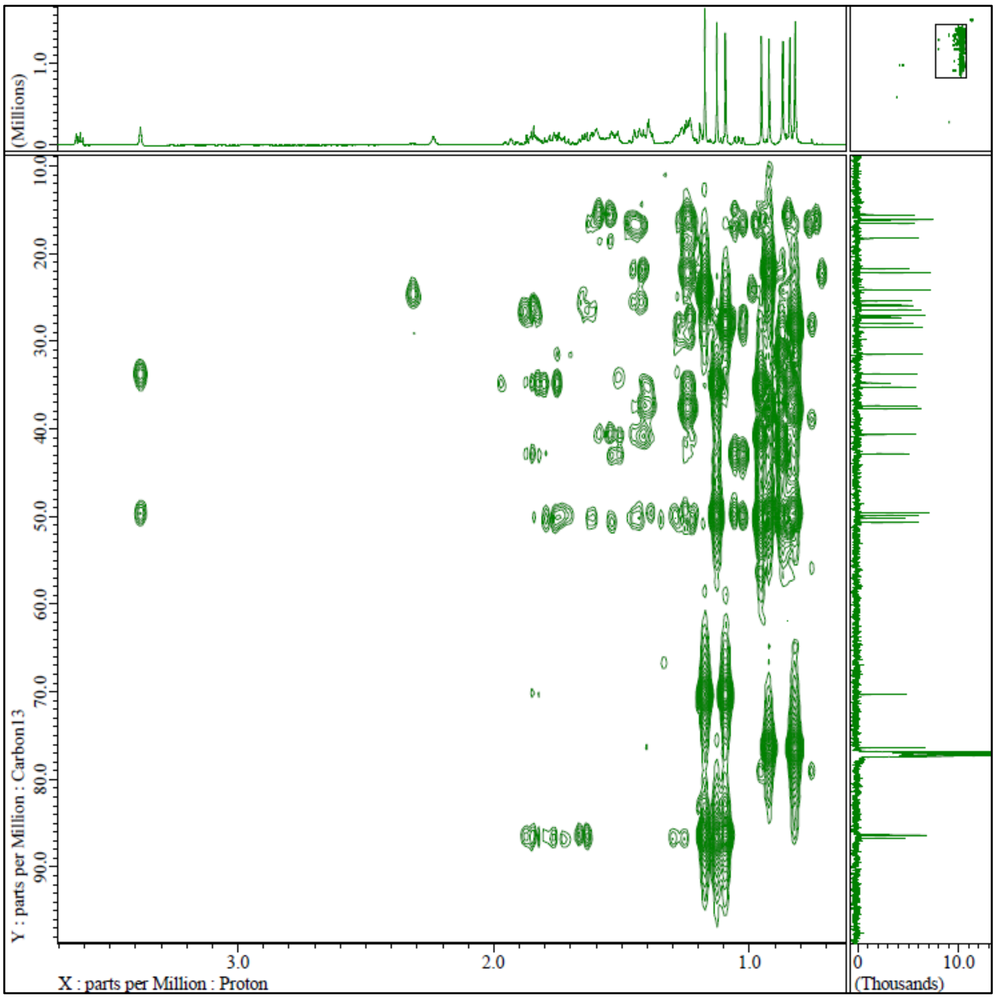

Figure S7. ^{1}H-^{1}H-COSY Spectrum of **1**.

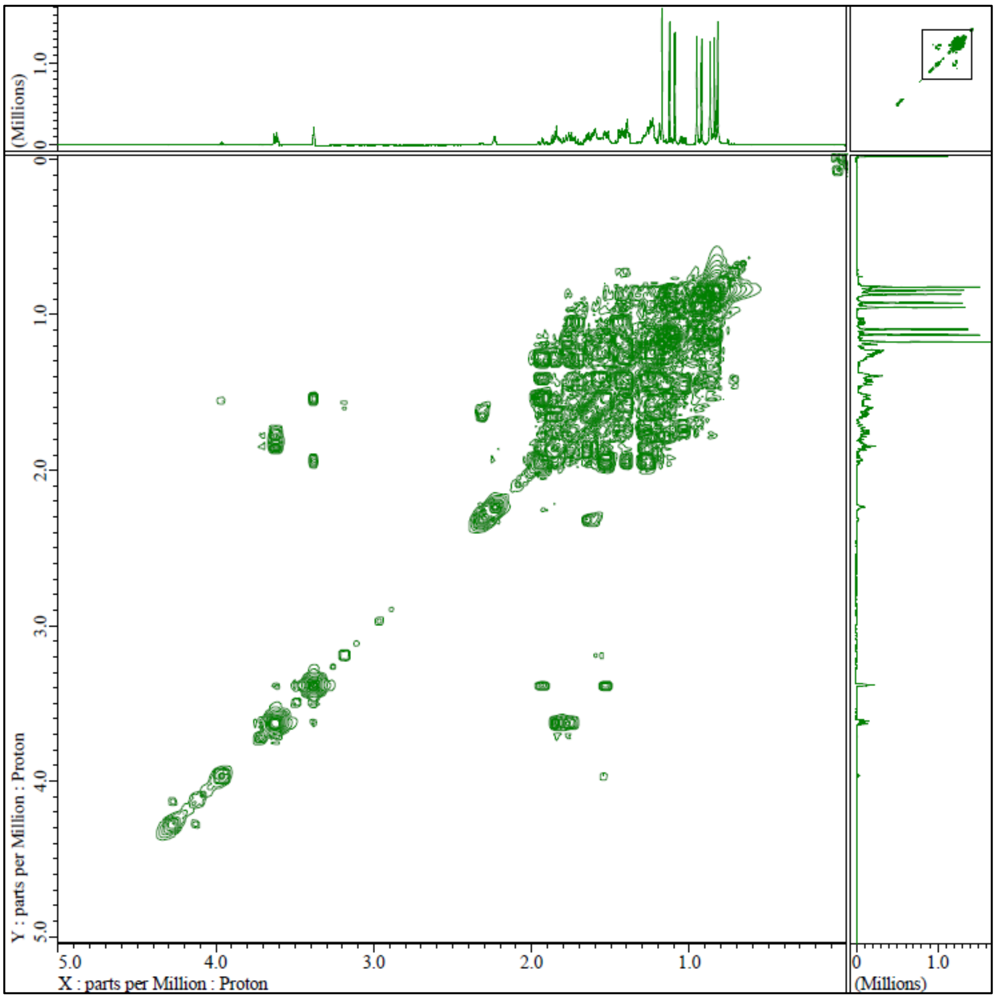

Figure S8. HRTOFMS Spectrum of **2**.

Single Mass Analysis
Tolerance = 50.0 mDa / DBE: min = -1.5, max = 50.0
Element prediction: Off
Number of isotope peaks used for i-FIT = 3

Monoisotopic Mass, Even Electron Ions
95 formula(e) evaluated with 6 results within limits (up to 50 closest results for each mass)
Elements Used:
C: 0-500 H: 0-1000 O: 0-200
KIN C 4 21 (0.376) Cm (19:21)
TOF MS ES+

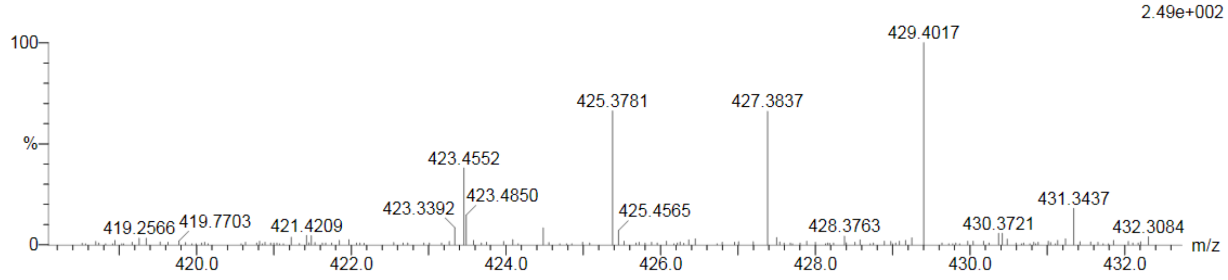

Minimum:				-1.5			
Maximum:		50.0	10.0	50.0			
Mass	Calc. Mass	mDa	PPM	DBE	i-FIT	i-FIT (Norm)	Formula
425.3781	425.3783	-0.2	-0.5	6.5	81.9	1.2	C30 H49 O

Figure S9. FTIR Spectrum of **2**.

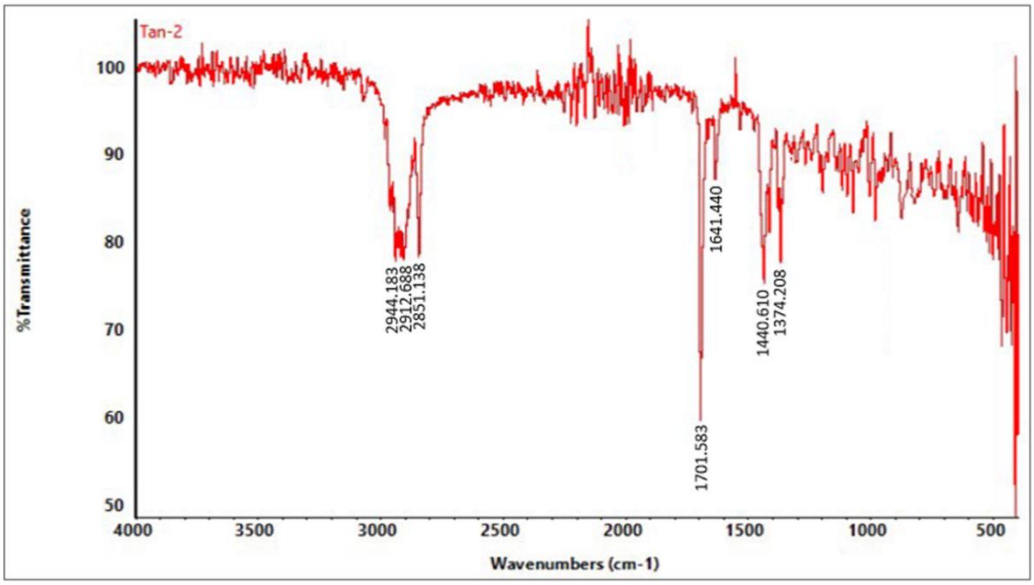

Figure S10. ^{1}H-NMR Spectrum of **2** (500 MHz in $CDCl_3$).

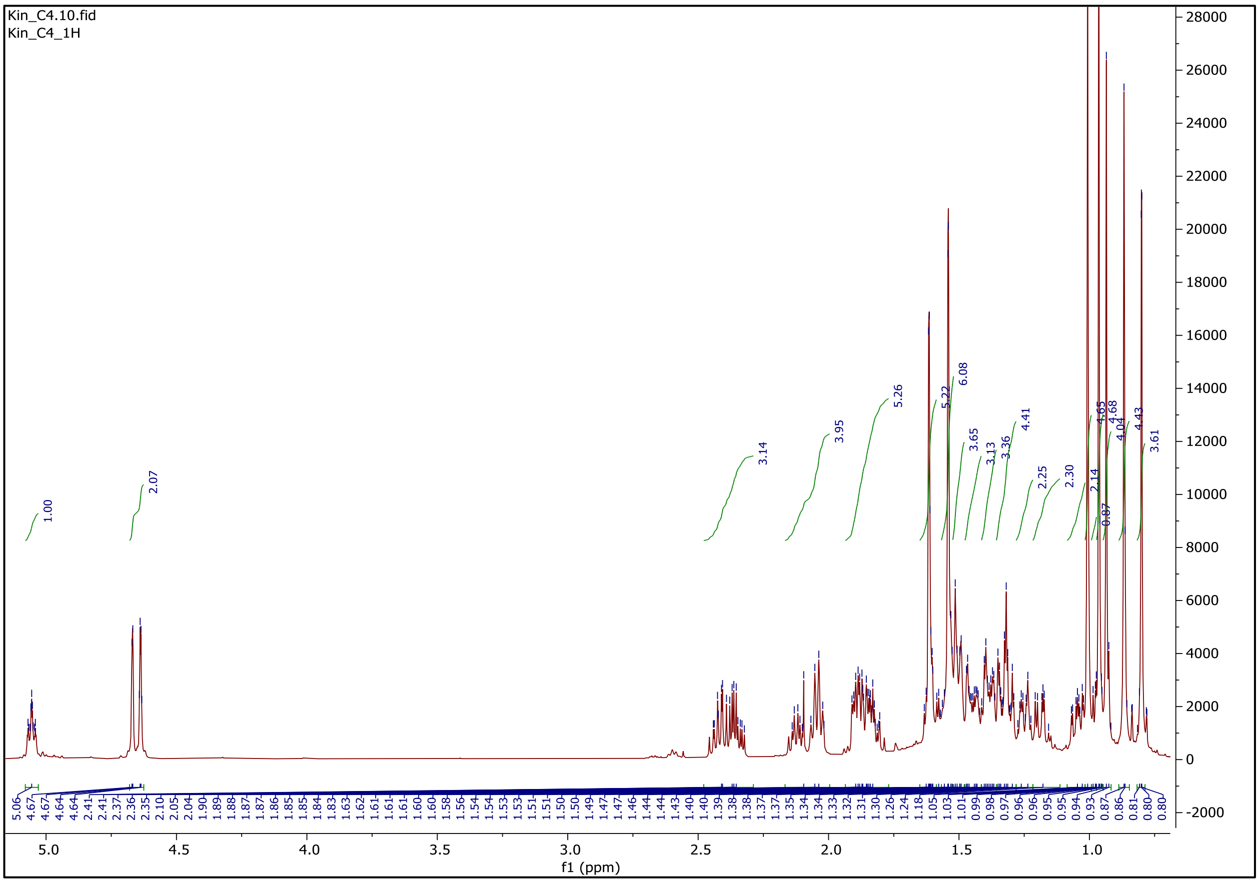

Figure S11. ^{13}C-NMR and DEPT-135° Spectrum of **2** (125 MHz in $CDCl_3$).

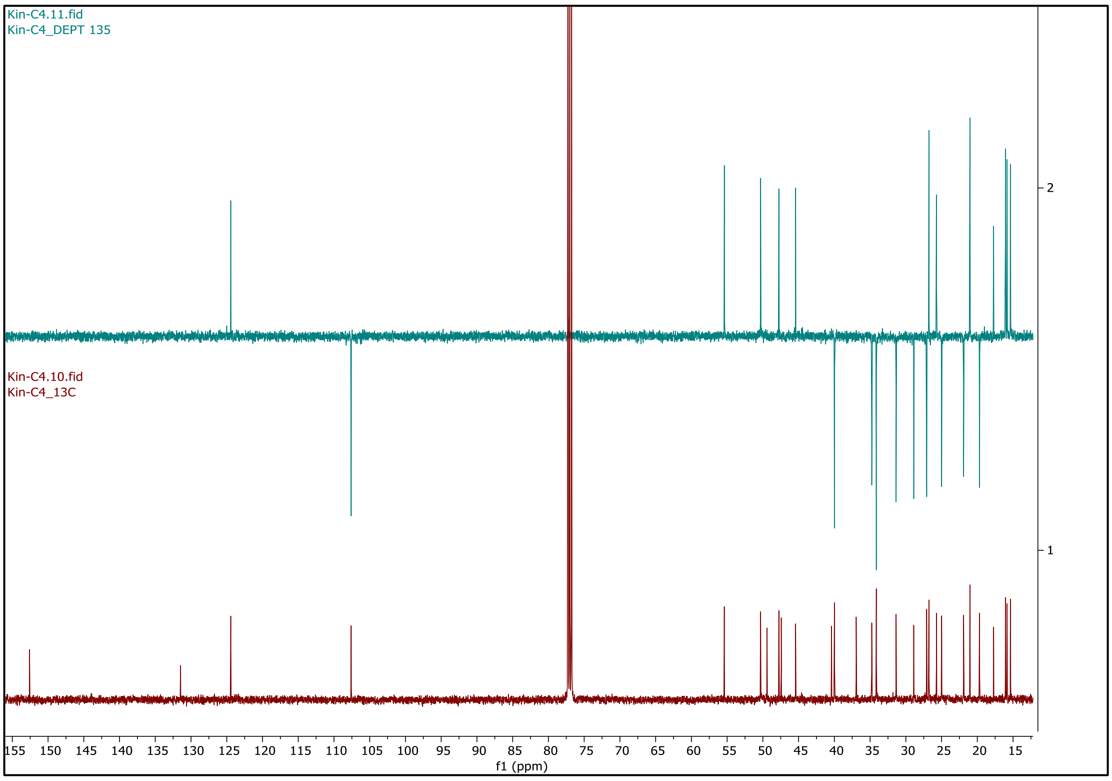

Figure S12. HRTOFMS Spectrum of **3**.

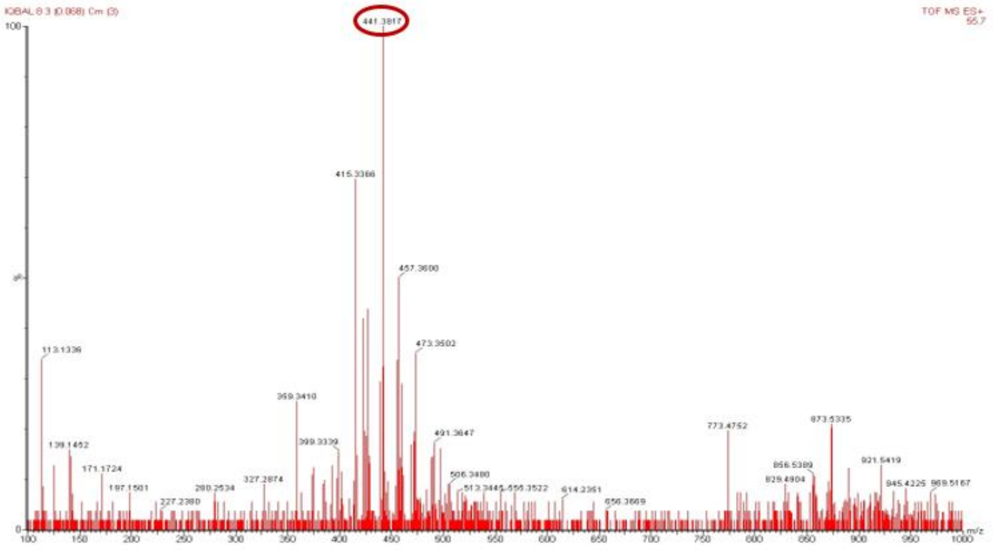

Mass	Calc. Mass	mDa	PPM	DBE	i-FIT	i-FIT (Norm)	Formula
441.3817	441.3811	-1.5	-3.4	6.5	13.7	0.0	C30 H51 O2

Figure S13. FTIR Spectrum of **3**.

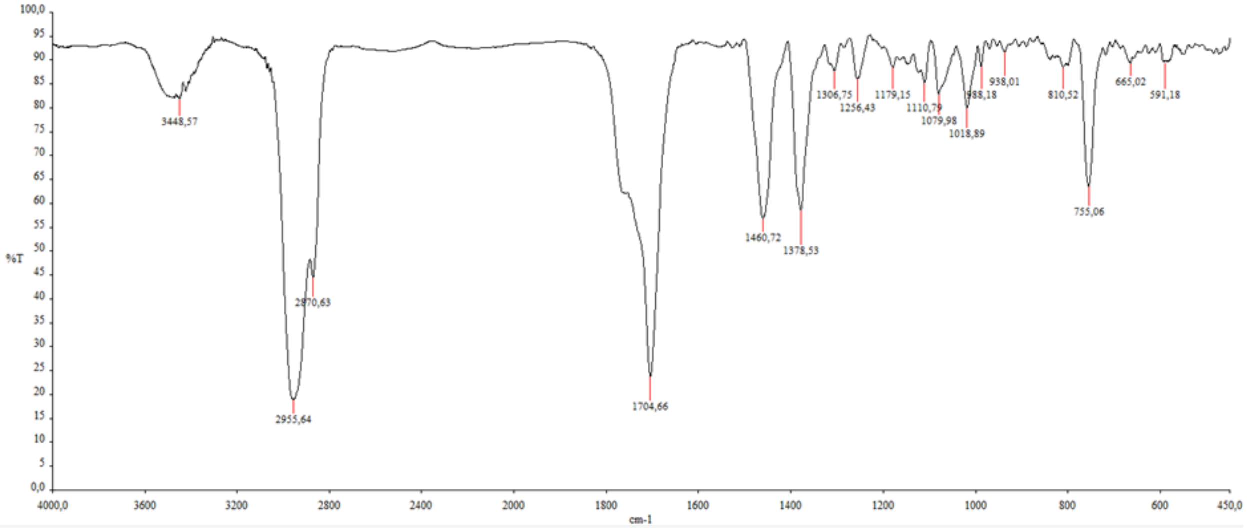

Figure S14. ^{1}H-NMR Spectrum of **3** (500 MHz in $CDCl_3$).

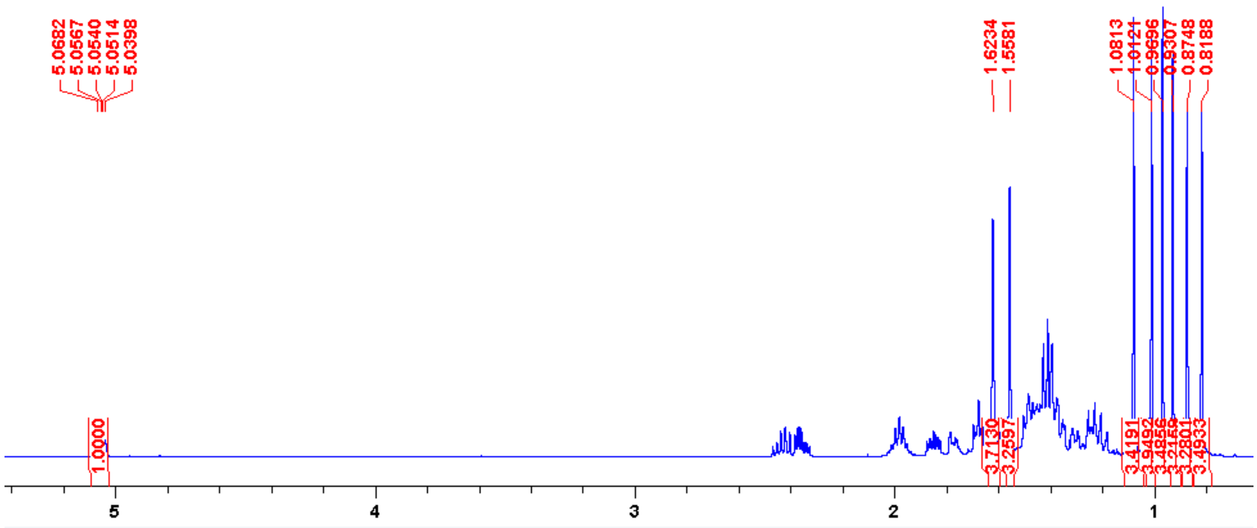

Figure S15. ^{13}C-NMR and DEPT-135° Spectrum of **3** (125 MHz in $CDCl_3$).

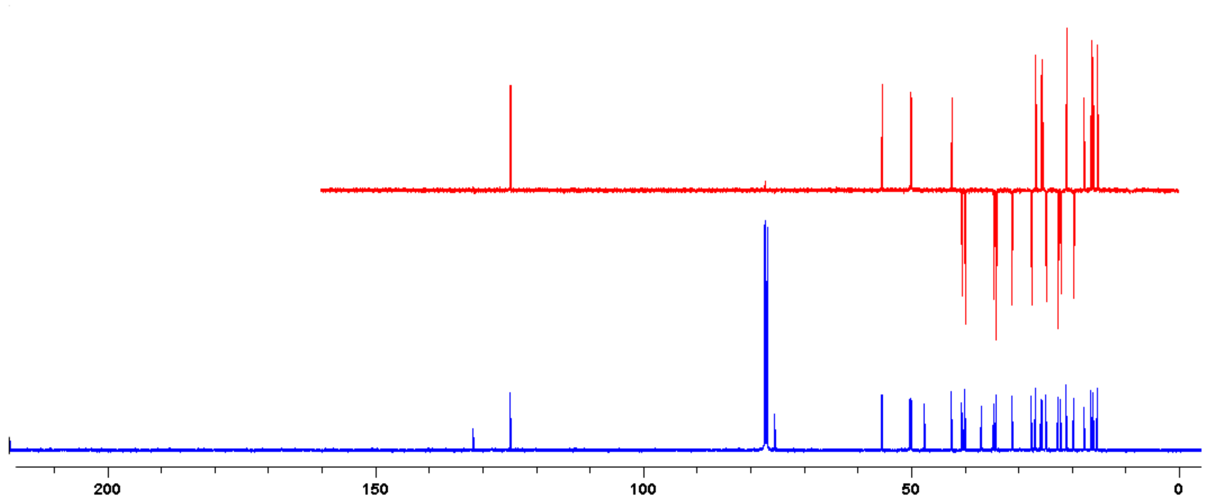

Figure S16. HRTOFMS Spectrum of **4**.

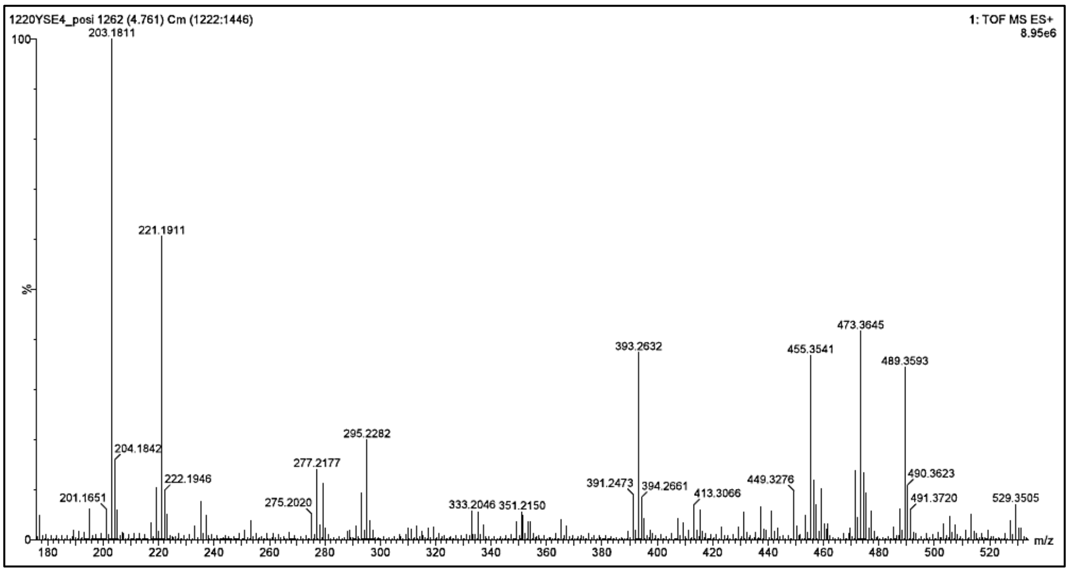

Figure S17. FTIR Spectrum of **4**.

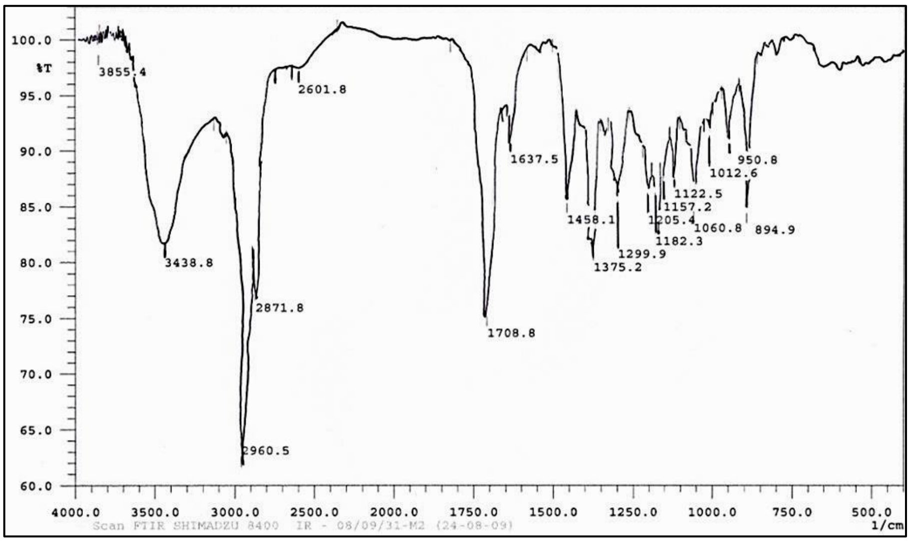

Figure S18. ^{1}H-NMR Spectrum of **4** (500 MHz in $CDCl_3$).

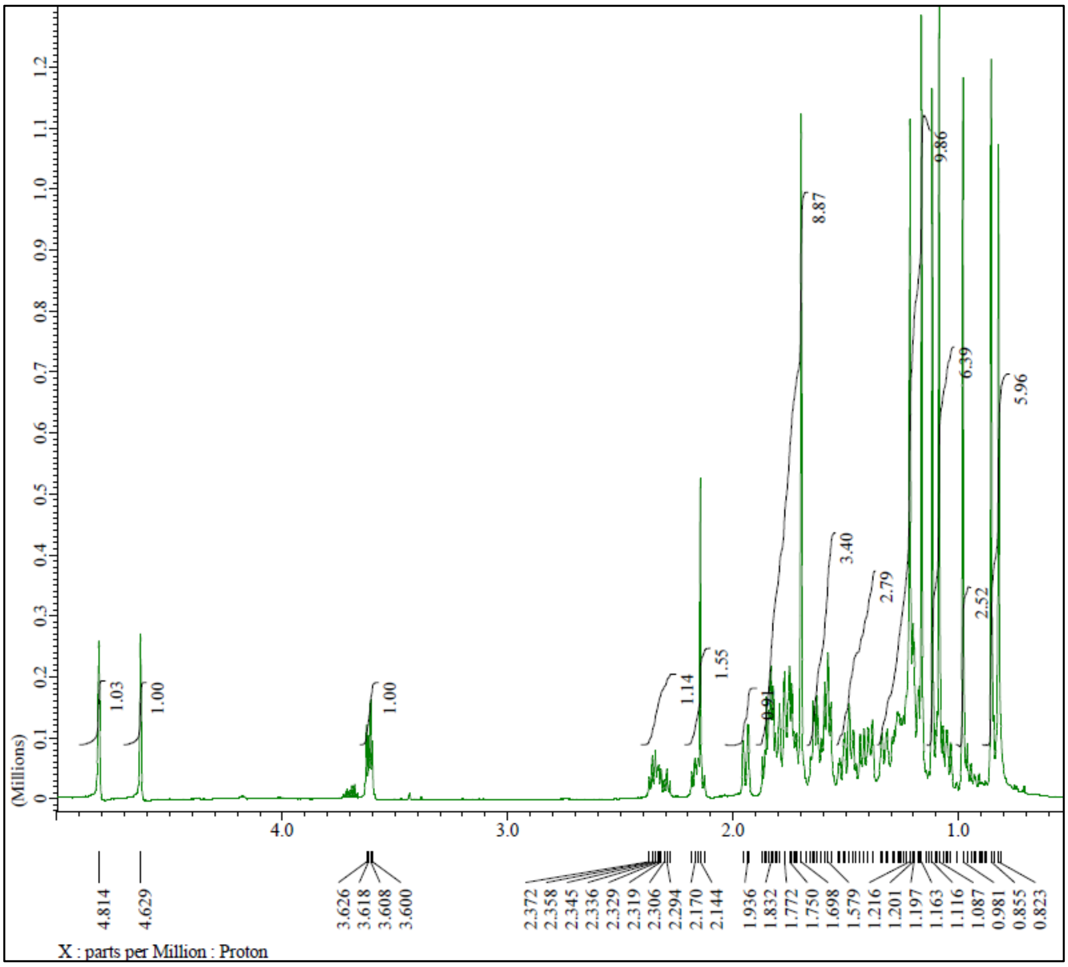

Figure S19. ^{13}C-NMR and DEPT-135° Spectrum of **4** (125 MHz in $CDCl_3$).

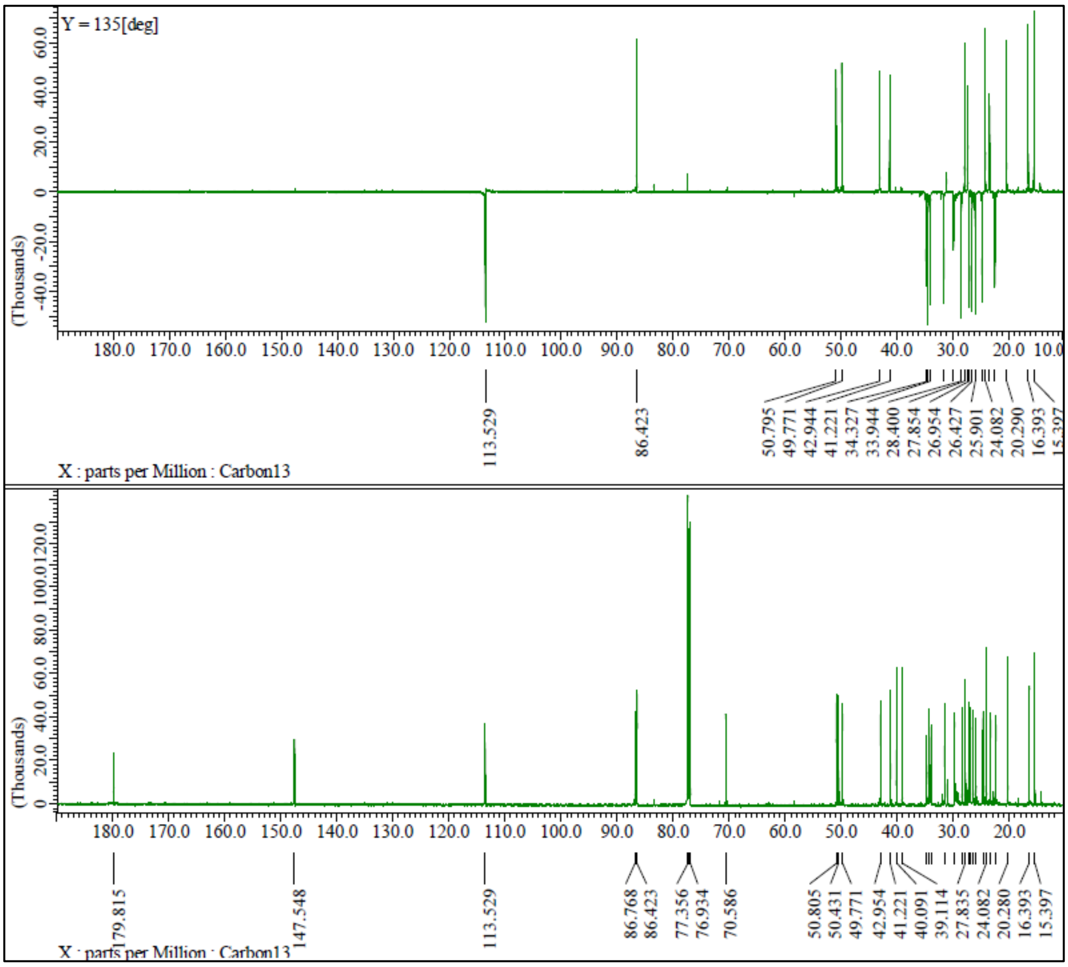

Figure S20. HMQC Spectrum of **4**.

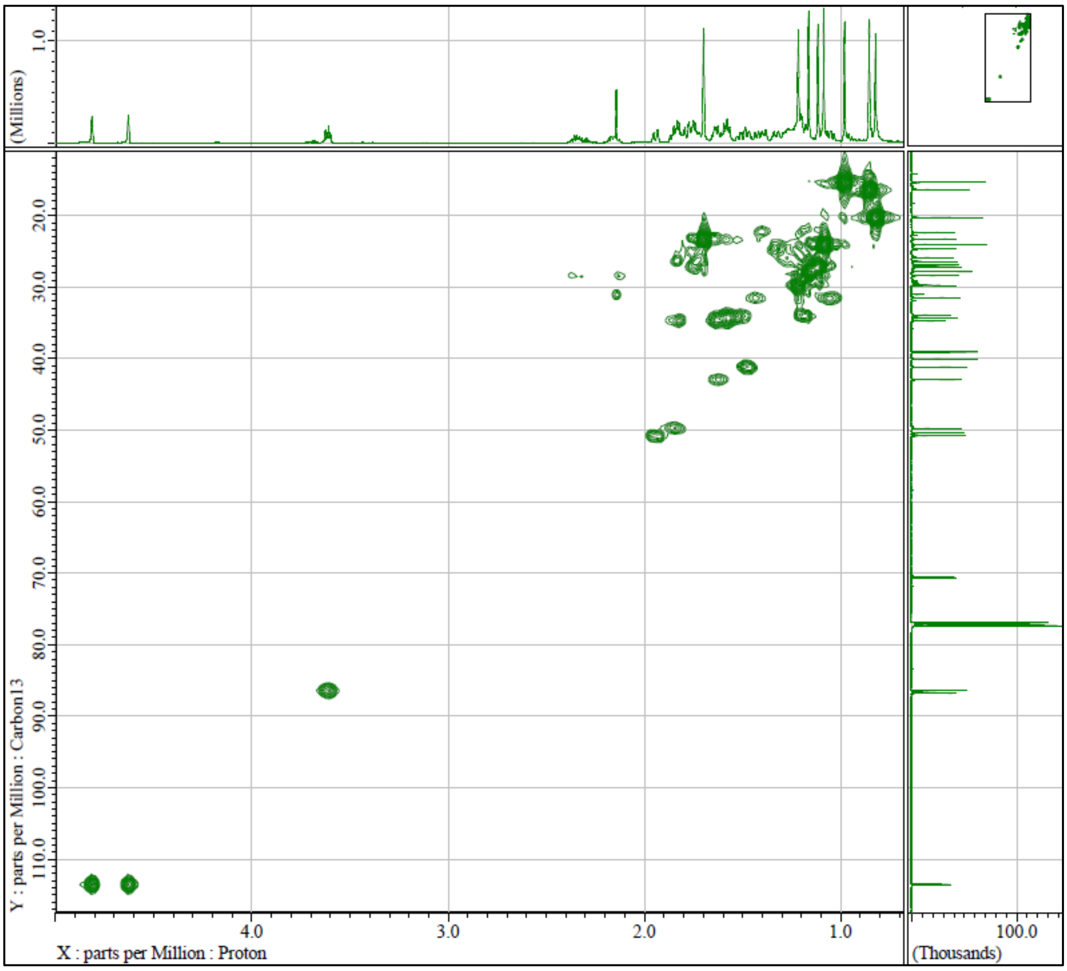

Figure S21. HMBC Spectrum of **4**.

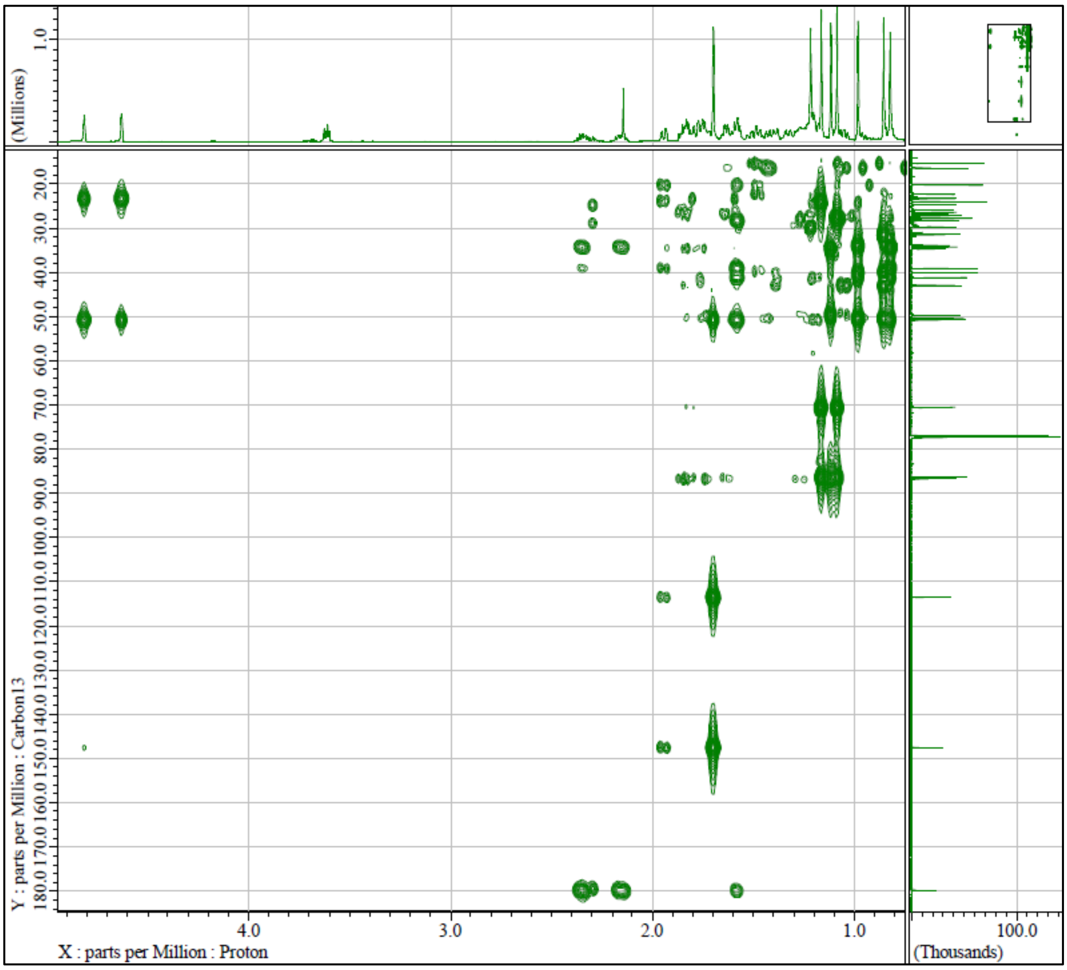

Figure S22. ^{1}H-^{1}H-COSY Spectrum of **4**.

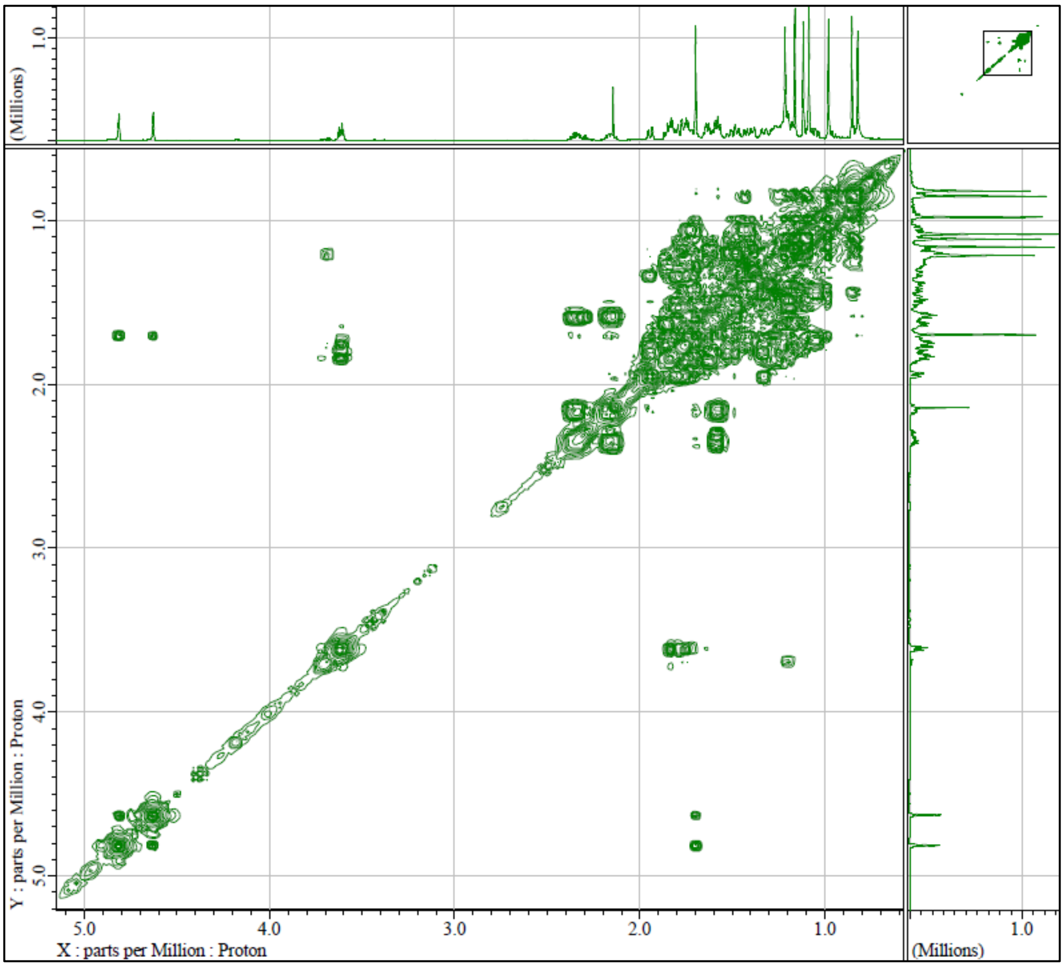

Figure S23. HRTOFMS Spectrum of **5**.

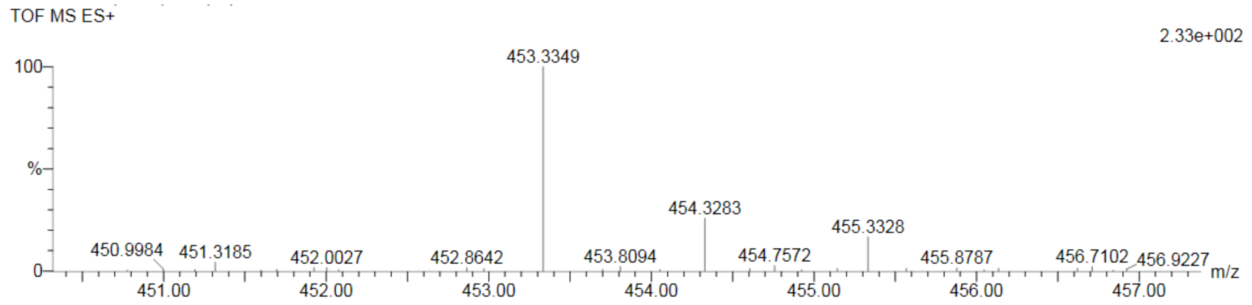

Minimum:				-1.5			
Maximum:		50.0	10.0	50.0			
Mass	Calc. Mass	mDa	PPM	DBE	i-FIT	i-FIT (Norm)	Formula
453.3349	453.3345	0.4	0.9	5.5	33.8	2.5	C28 H46 O3 Na

Figure S24. FTIR Spectrum of **5**.

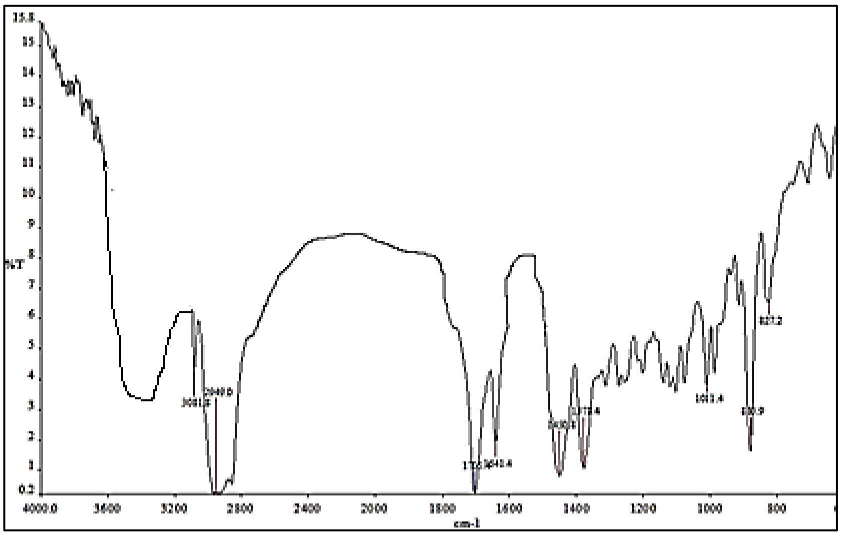

Figure S25. ^{1}H-NMR Spectrum of **5** (500 MHz in $CDCl_3$).

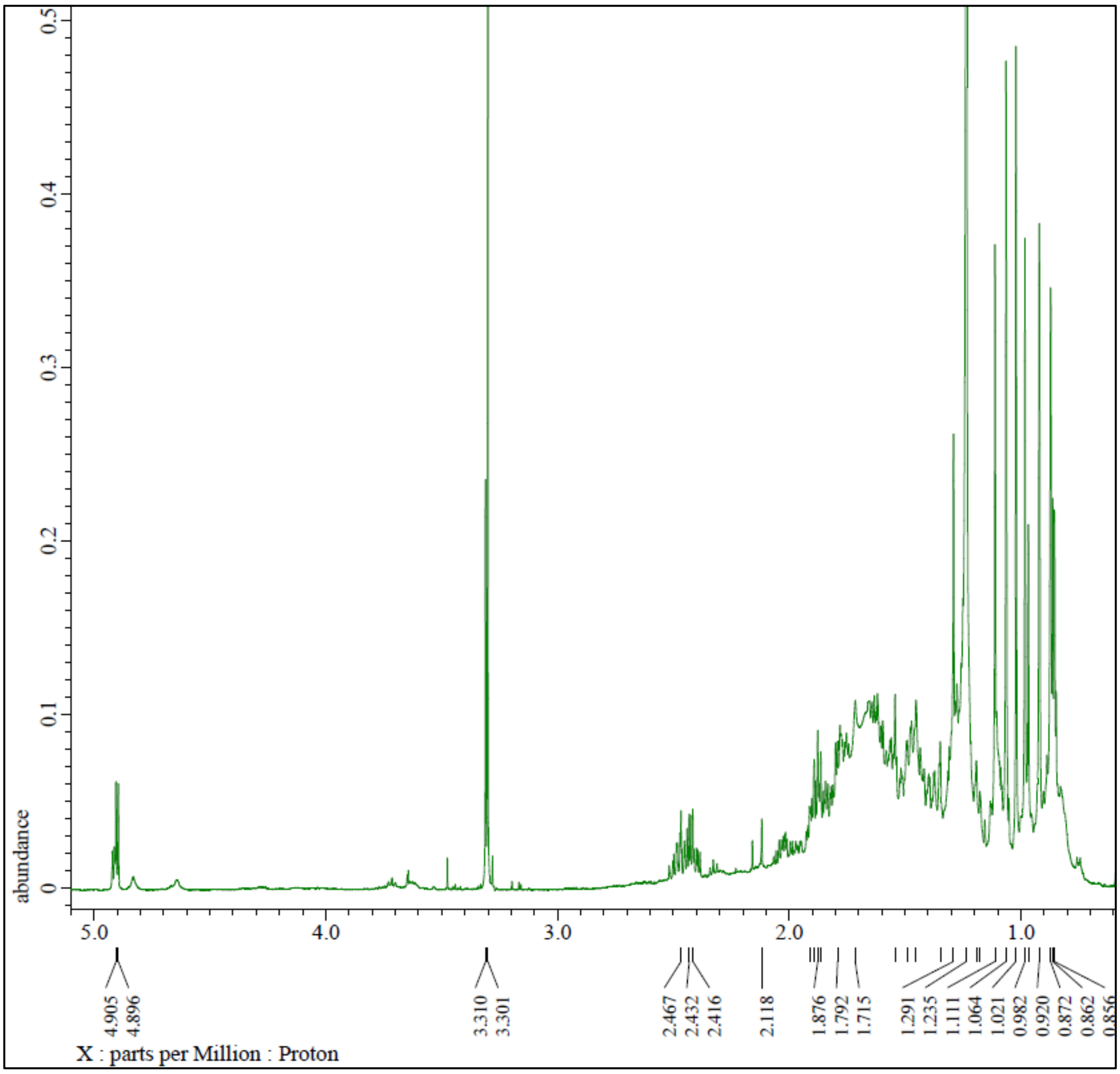

Figure S26. ^{13}C-NMR and DEPT-135° Spectrum of **5** (125 MHz in $CDCl_3$).

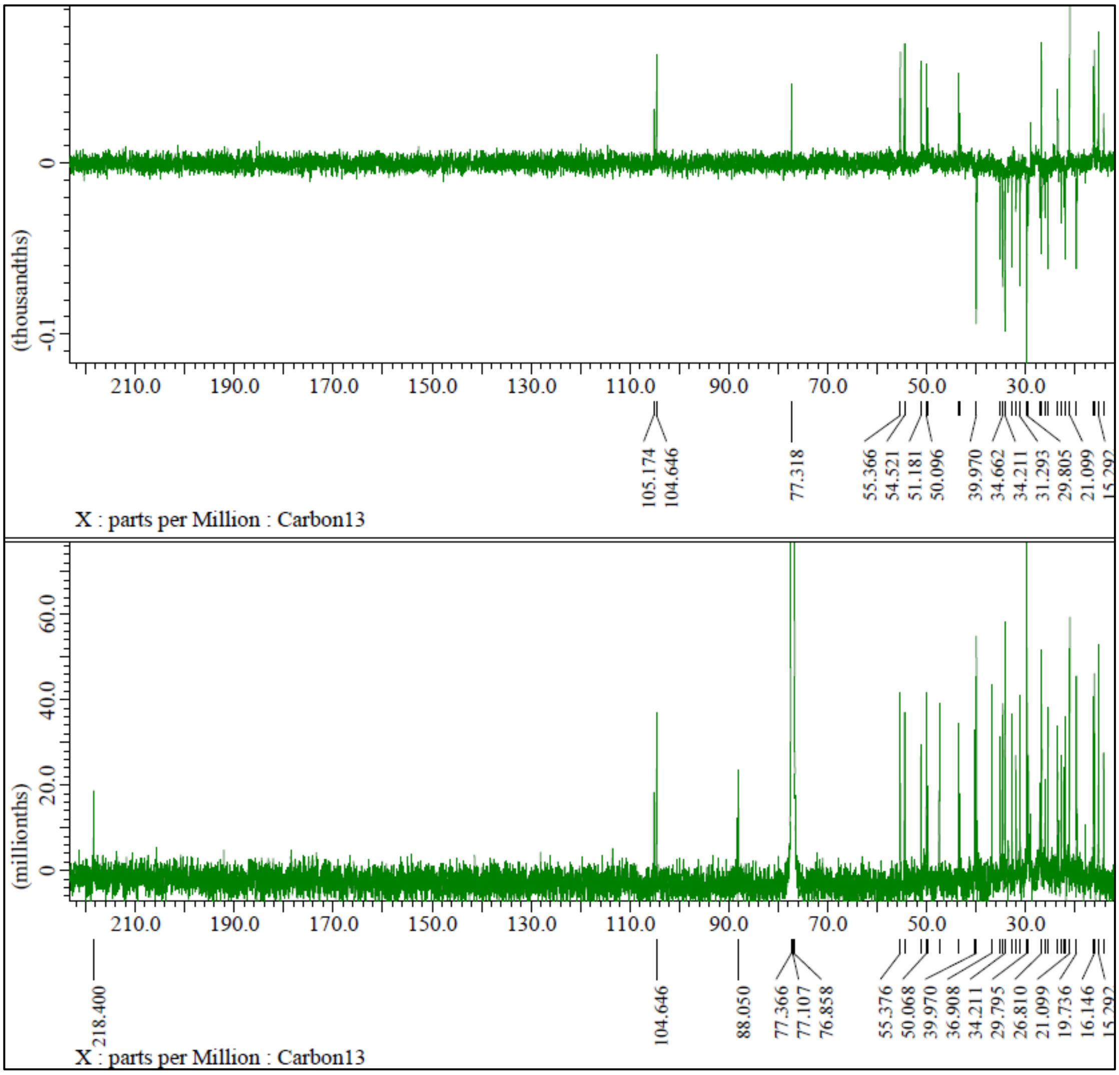

Figure S27. HMQC Spectrum of **5**.

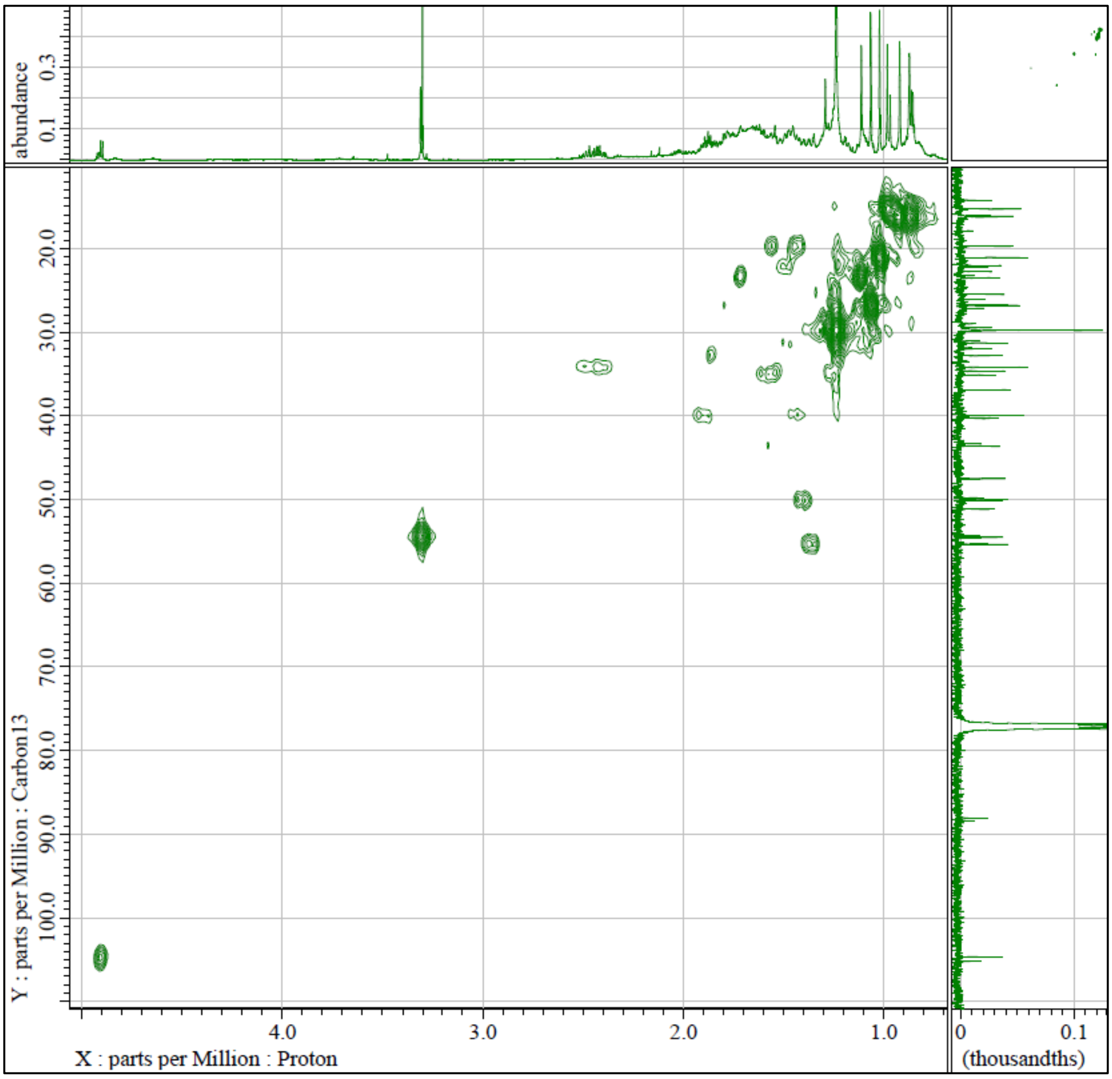

Figure S28. HMBC Spectrum of **5**.

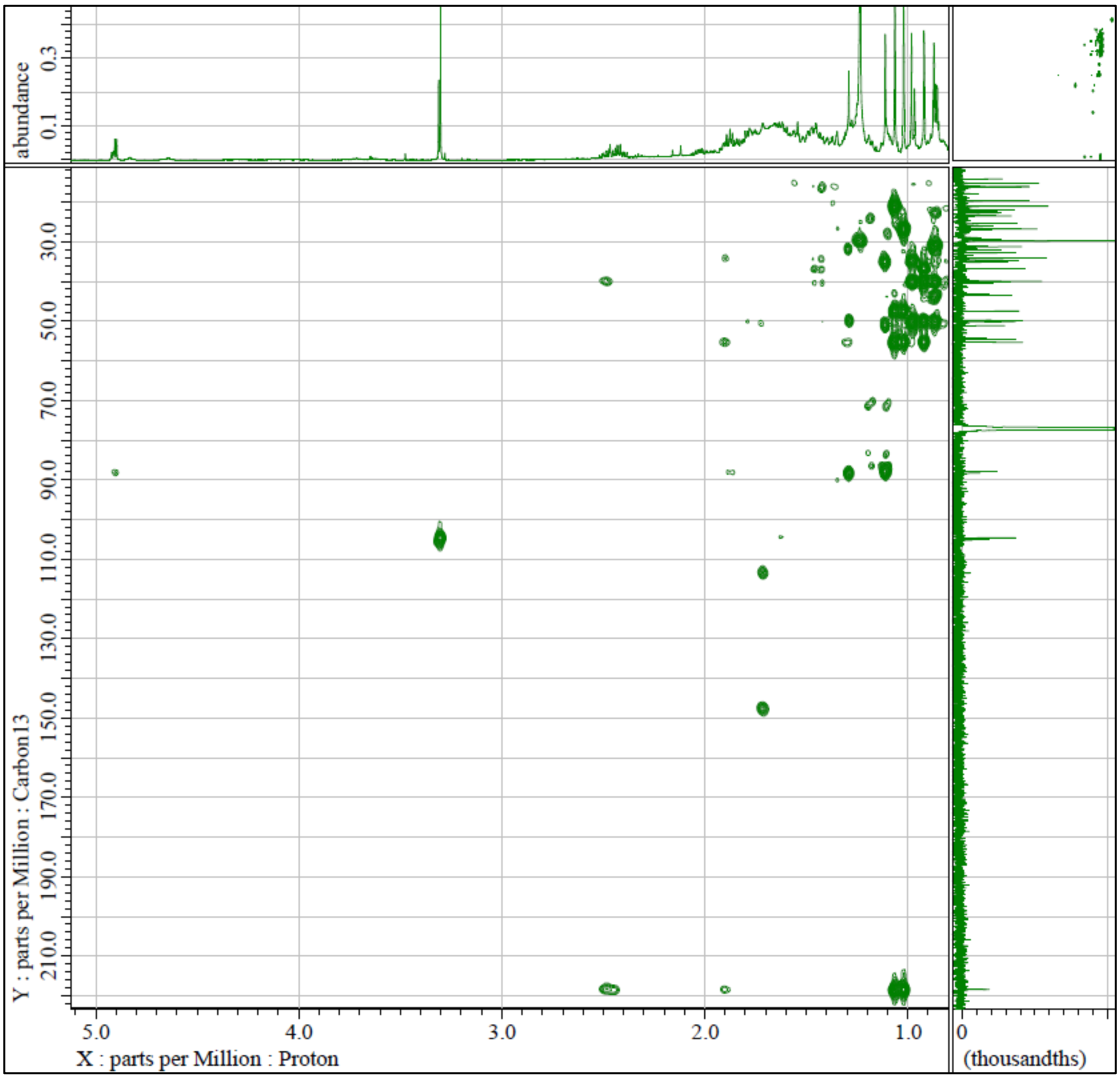

Figure S29. ^{1}H-^{1}H-COSY Spectrum of **5**.

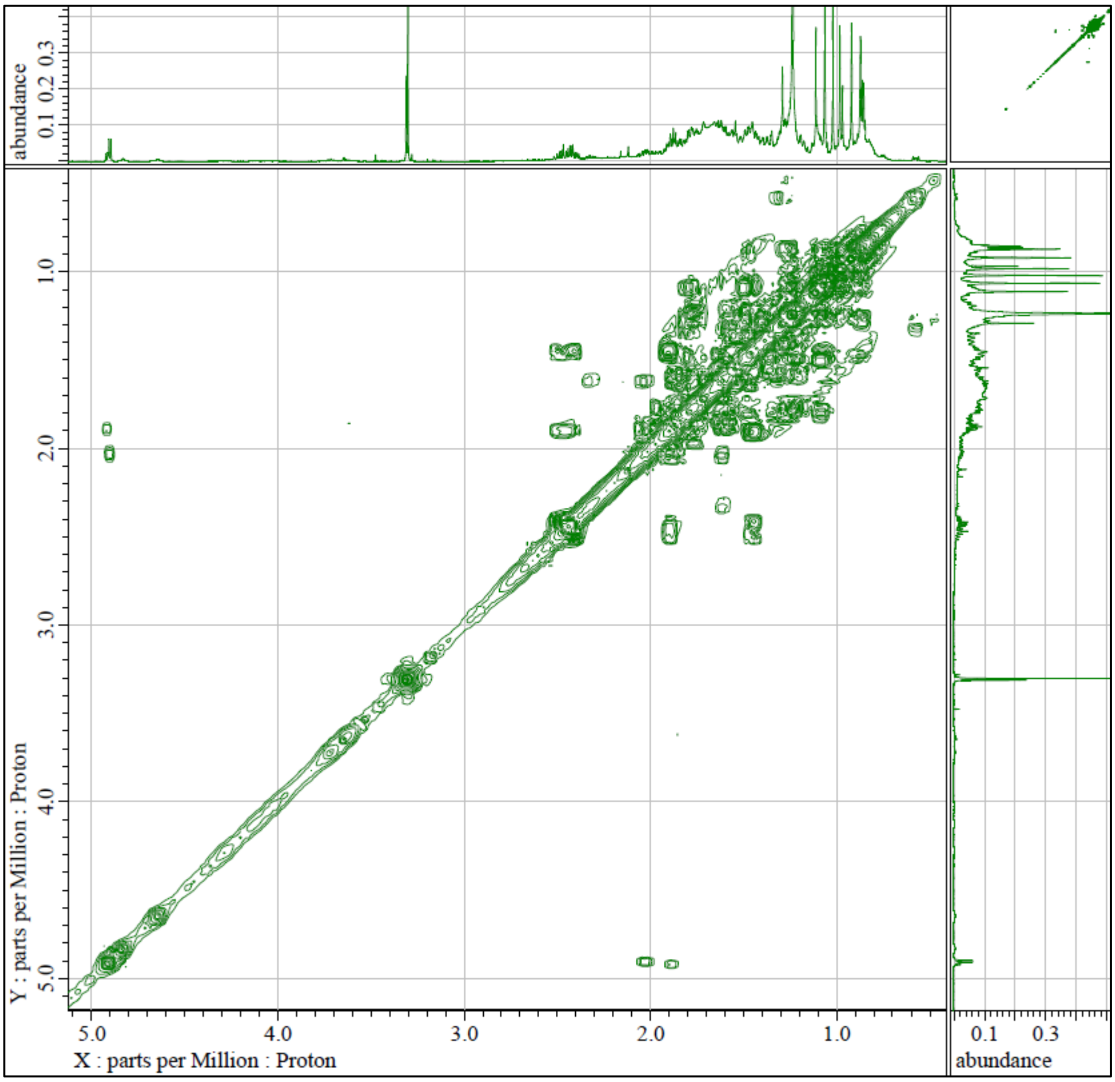

Figure S30. HRTOFMS Spectrum of **6**.

Single Mass Analysis
Tolerance = 50.0 mDa / DBE: min = -1.5, max = 50.0
Element prediction: Off
Number of isotope peaks used for i-FIT = 3

Monoisotopic Mass, Even Electron Ions
222 formula(e) evaluated with 21 results within limits (up to 50 closest results for each mass)
Elements Used:
C: 0-500 H: 0-1000 O: 0-200 Na: 0-1
KIN C 15 22 (0.393) Cm (22)
TOF MS ES+

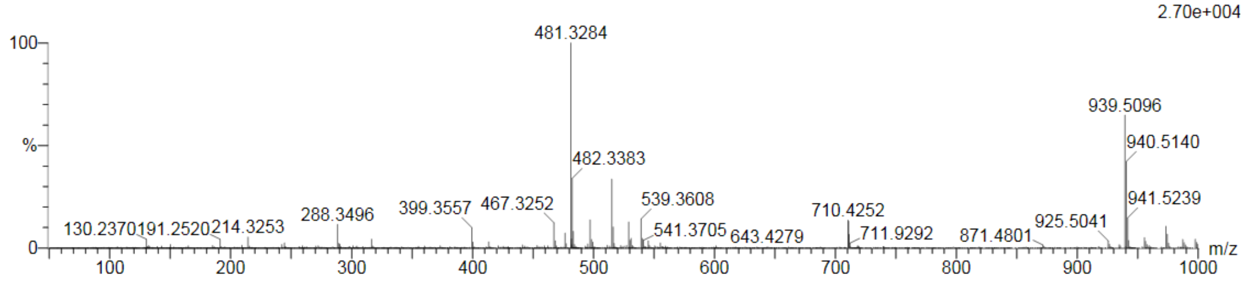

Minimum:				-1.5			
Maximum:		50.0	10.0	50.0			
Mass	Calc. Mass	mDa	PPM	DBE	i-FIT	i-FIT (Norm)	Formula
481.3284	481.3294	-1.0	-2.1	6.5	99.1	2.9	C29 H46 O4 Na

Figure S31. FTIR Spectrum of **6**.

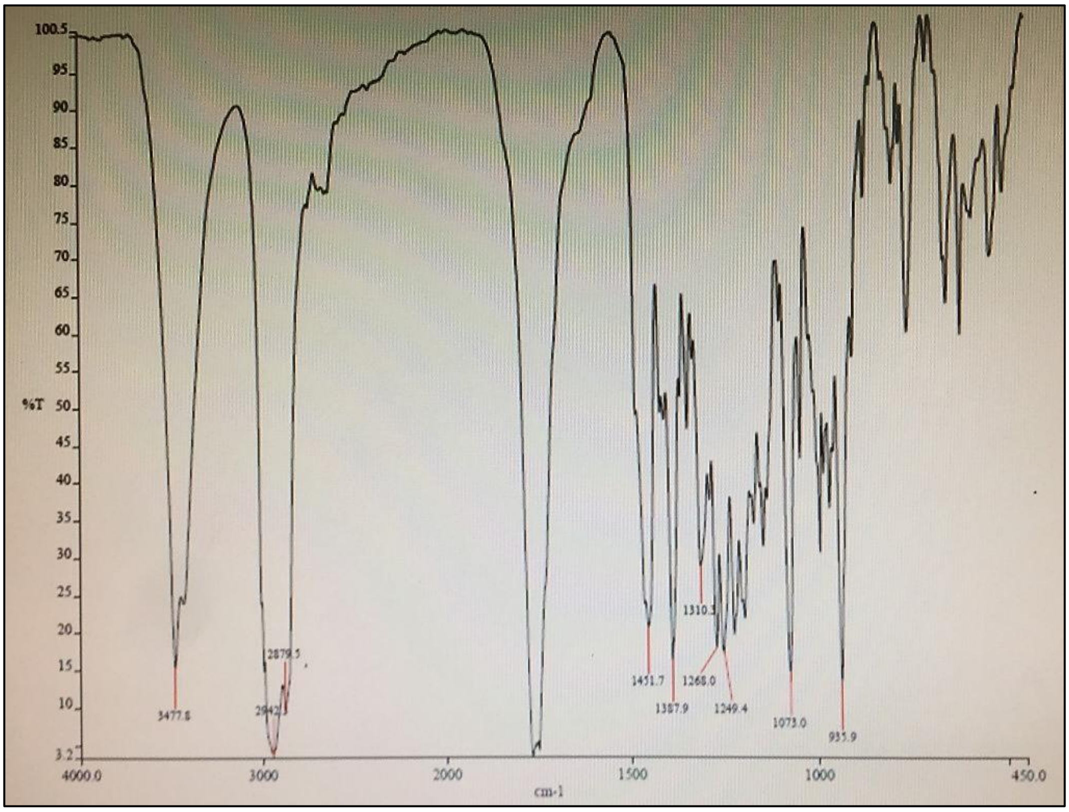

Figure S32. ^{1}H-NMR Spectrum of **6** (500 MHz in $CDCl_3$).

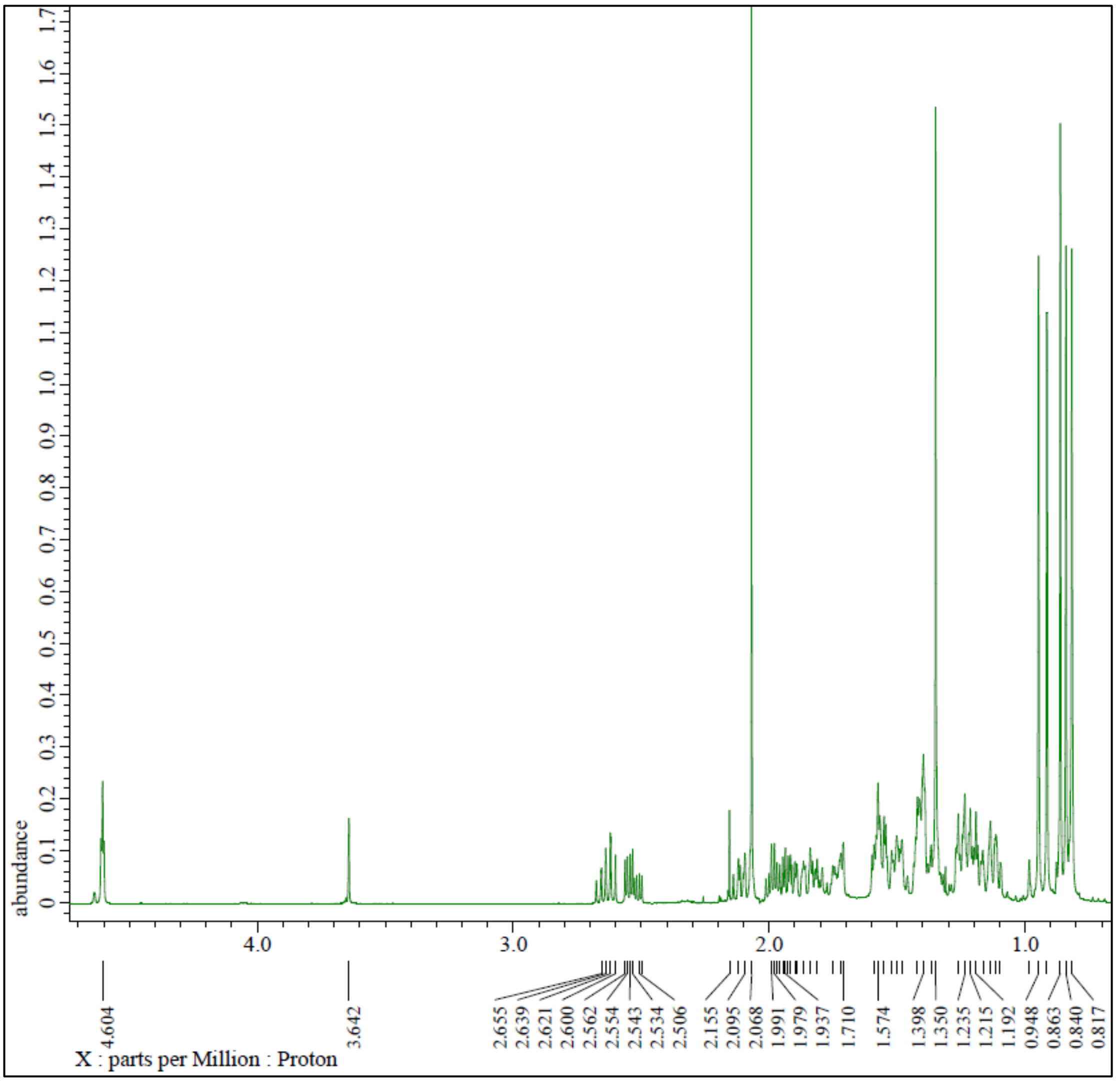

Figure S33. ^{13}C-NMR and DEPT-135° Spectrum of **6** (125 MHz in $CDCl_3$).

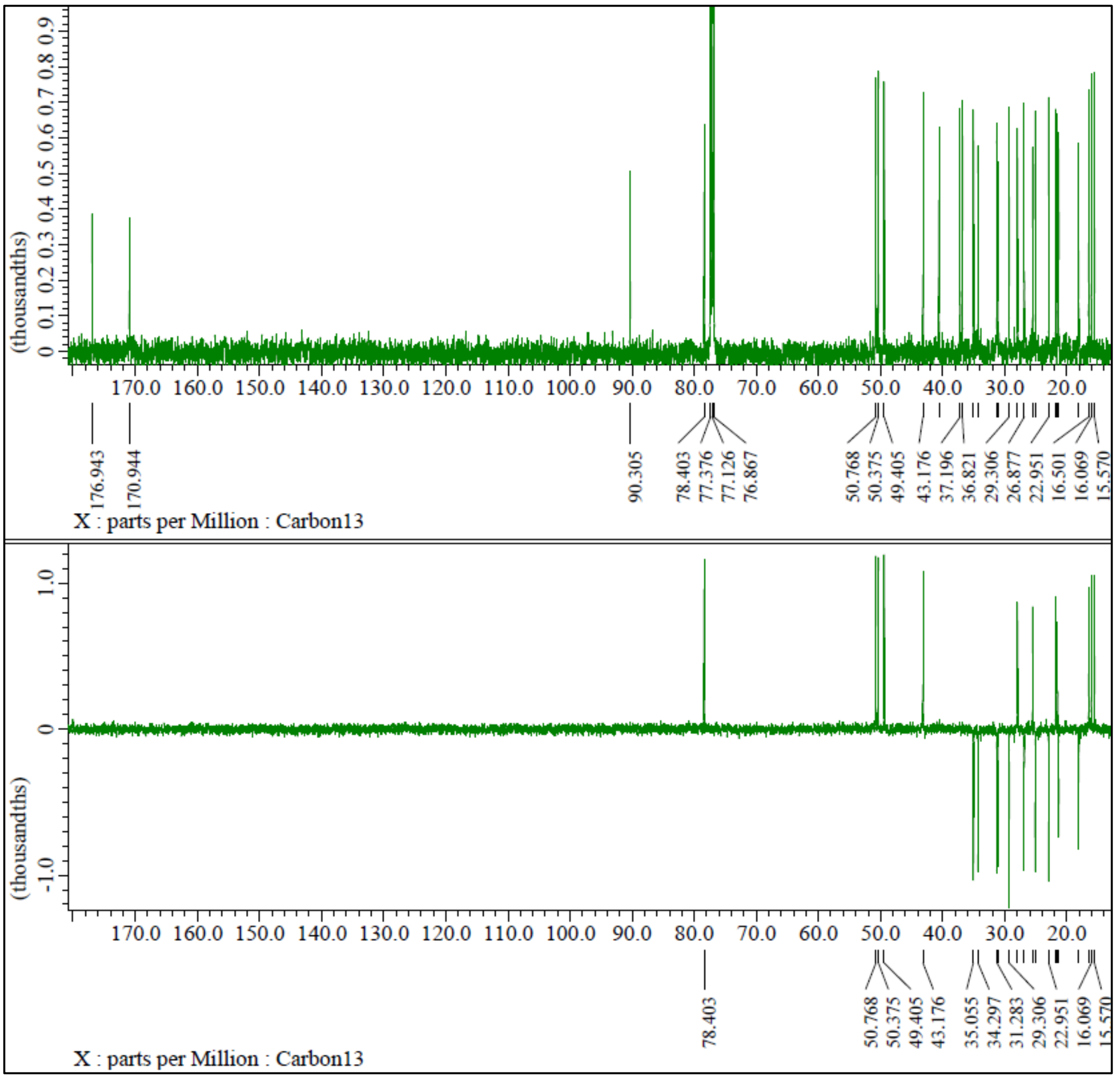

Figure S34. HRTOFMS Spectrum of **7**.

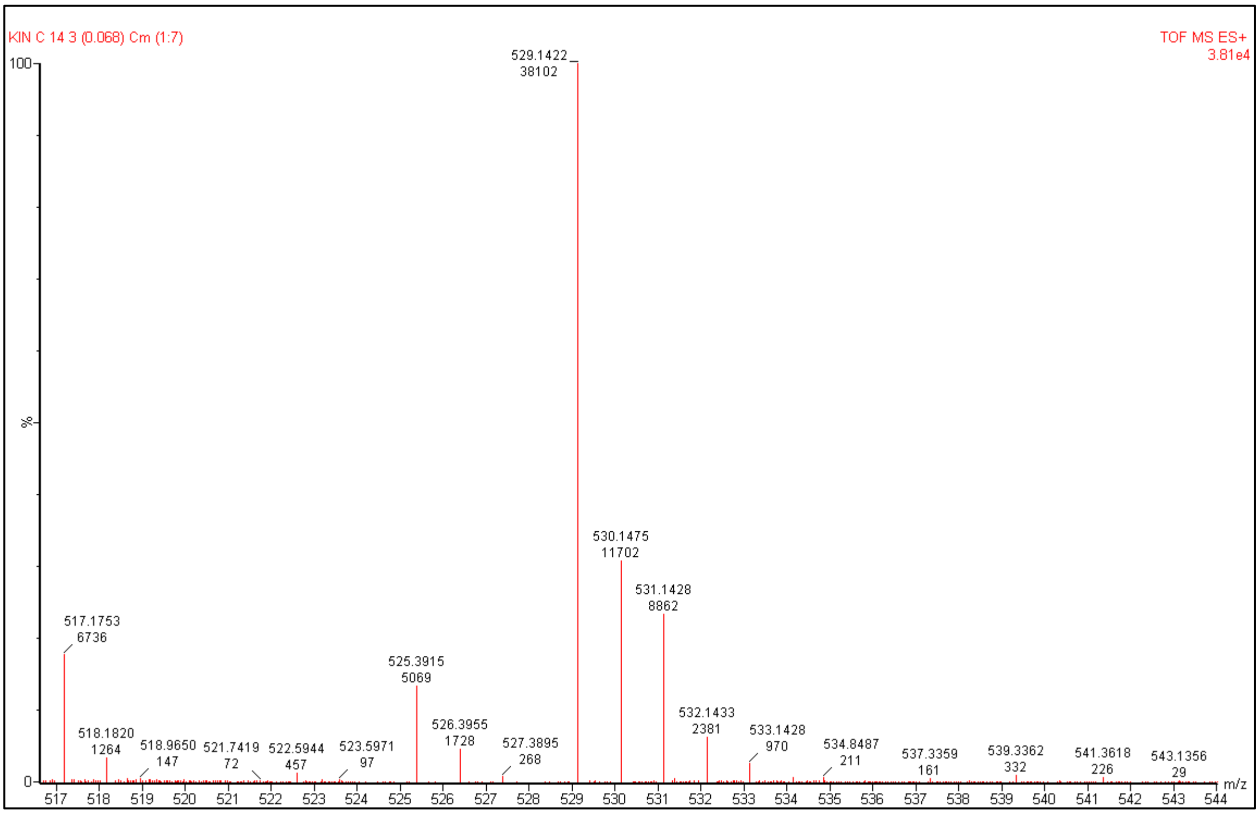

Figure S35. FTIR Spectrum of **7**.

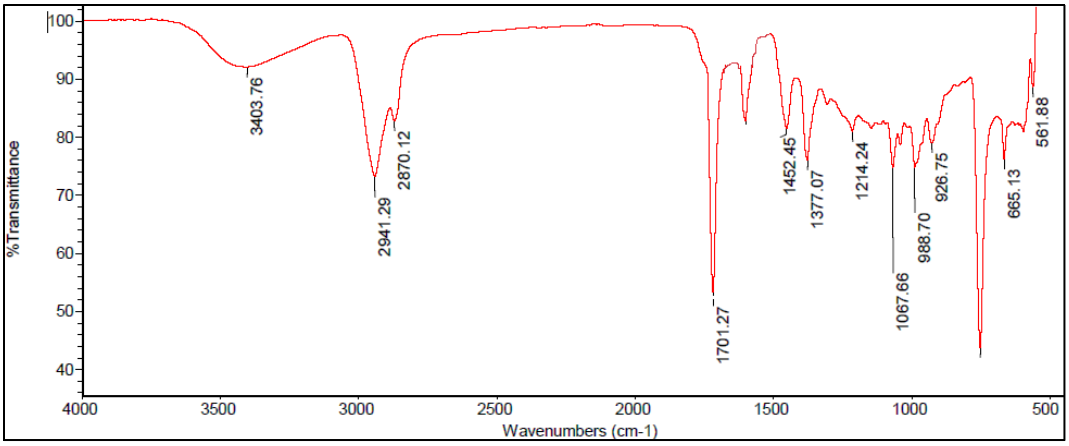

Figure S36. ^{1}H-NMR Spectrum of **7** (500 MHz in $CDCl_3$).

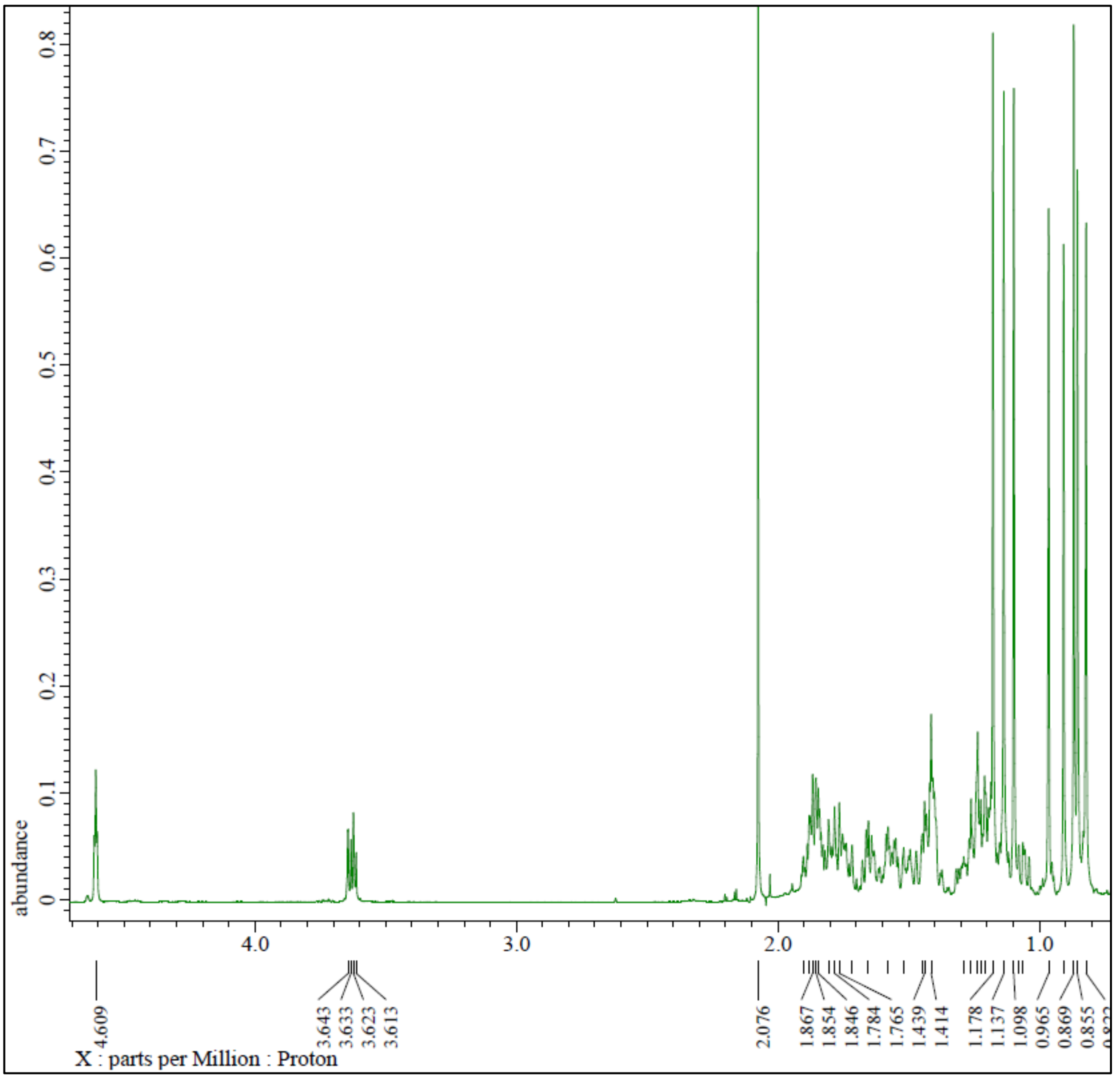

Figure S37. ^{13}C-NMR and DEPT-135° Spectrum of **7** (125 MHz in $CDCl_3$).

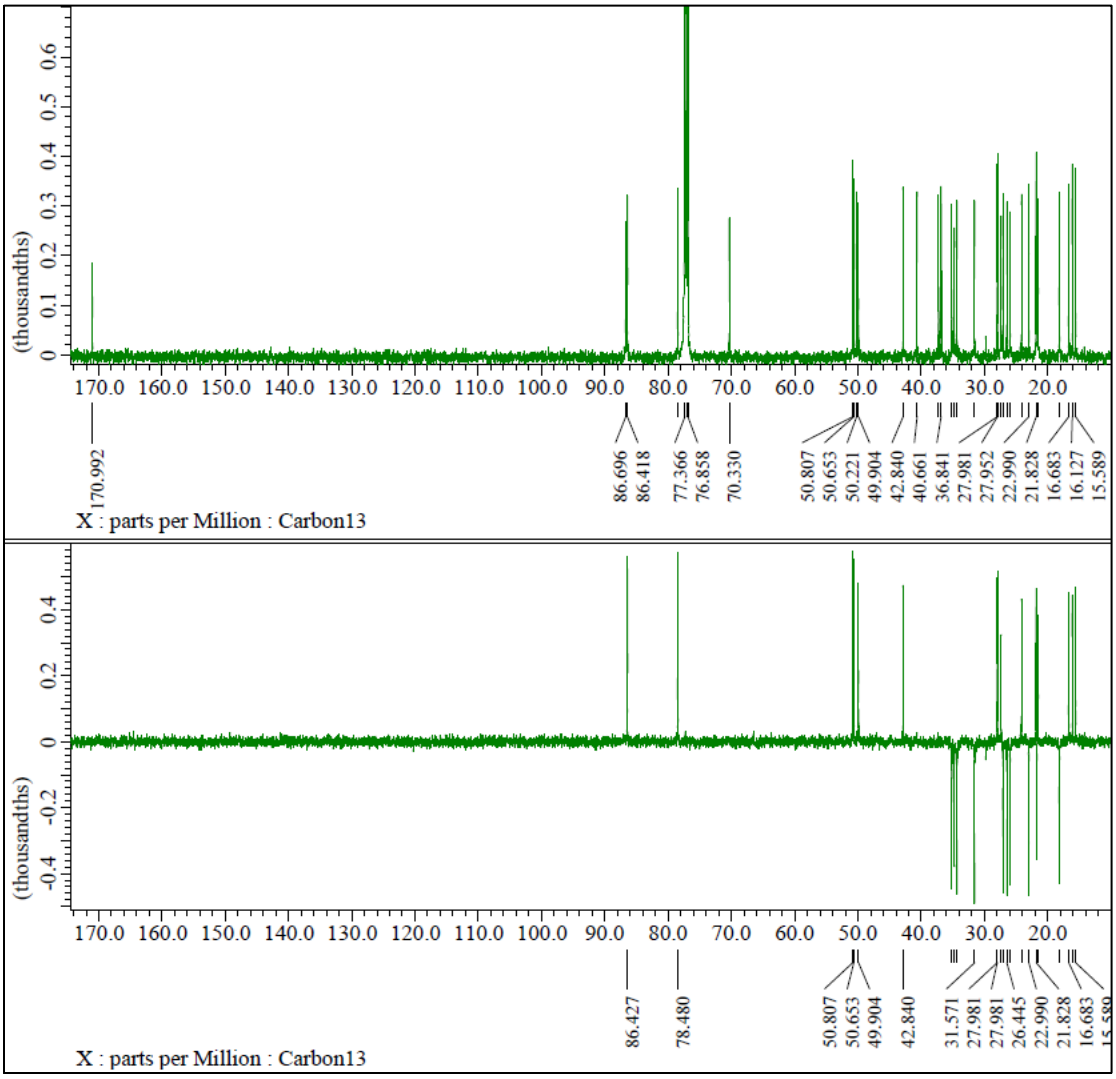

Figure S38. Results of cytotoxic activity of **1** against MCF-7 cell line.

	Media	Media+Cell	Cisplatin	DMSO 2,00%	Sample concentrations (μg/mL)							
					300.00	150.00	75.00	37.50	18.75	9.38	4.69	2.34
Absorbance 570nm	0.4499	0.7129	0.5763	0.6898	0.4461	0.4495	0.5270	0.6792	0.7836	0.7261	0.7223	0.7040
	0.4585	0.6953	0.5720	0.7189	0.4649	0.3713	0.4720	0.6561	0.6677	0.7268	0.6984	0.7247
Absorbance 600nm	0.5814	0.2328	0.4878	0.2131	0.5641	0.5759	0.4240	0.2393	0.2636	0.2287	0.2204	0.2159
	0.5950	0.2173	0.4902	0.2220	0.5952	0.4749	0.3840	0.2311	0.2073	0.2255	0.2135	0.2290
Absorbance difference	-0.1315	0.4801	0.0885	0.4767	-0.1180	-0.1264	0.1030	0.4399	0.5200	0.4974	0.5019	0.4881
	-0.1365	0.4780	0.0818	0.4969	-0.1303	-0.1036	0.0880	0.4250	0.4604	0.5013	0.4849	0.4957
% live cell		98.92	35.84	98.37	2.58	1.22	38.18	92.45	105.35	101.71	102.43	100.21
		98.58	34.76	101.63	0.60	4.90	35.76	90.05	95.75	102.34	99.69	101.43
Average % live cell		98.75	35.30	100.00	1.59	3.06	36.97	91.25	100.55	102.02	101.06	100.82
SEM		0.17	0.54	1.63	0.99	1.84	1.21	1.20	4.80	0.31	1.37	0.61
Normalize data		98.75	35.30	100.00	1.59	3.06	36.97	91.25	100.55	102.02	101.06	100.82

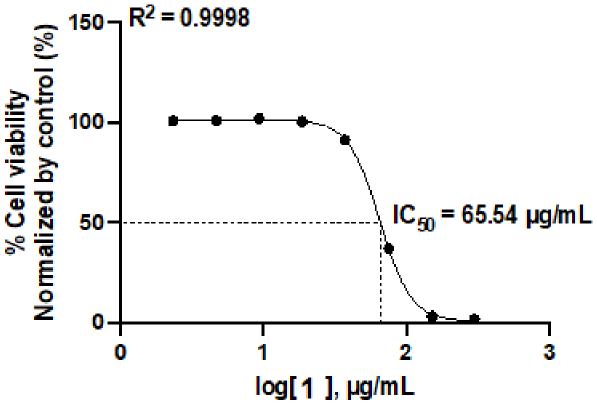

Media	Media +cell	Cisplatin	Solvent	Sample concentrations (μg/mL)							
				500,00	250,00	125,00	62,50	31,25	15,63	7,81	3,91

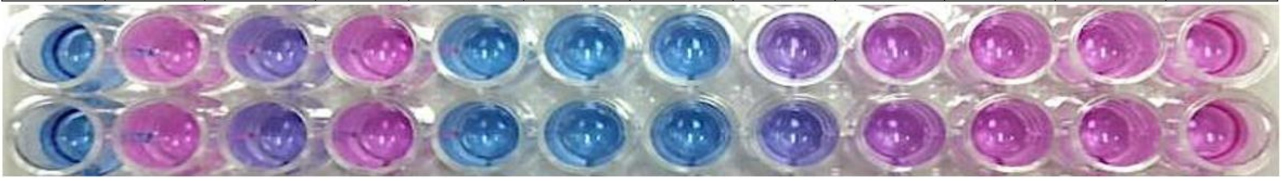

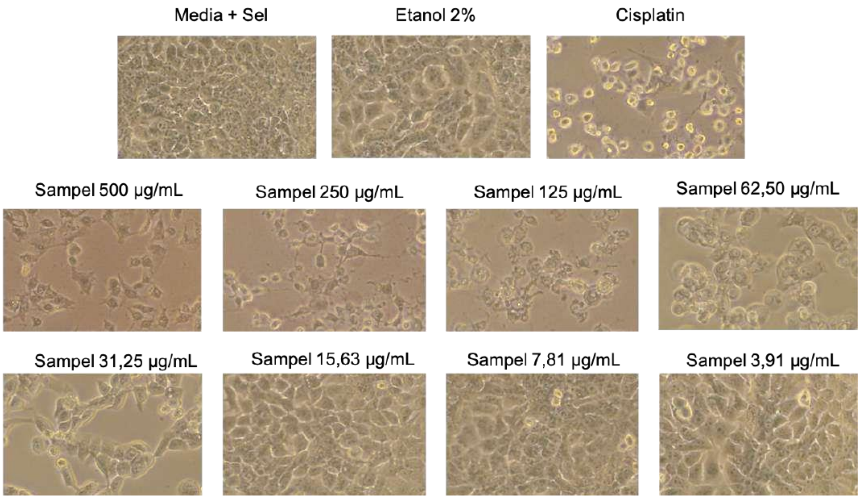

MCF-7 cell morphology at each concentration of compound **1**

Figure S39. Results of cytotoxic activity of **2** against MCF-7 cell line.

	Media	Media+Cell	Cisplatin	DMSO 2,00%	Sample concentrations (µg/mL)							
					500.00	250.00	125.00	62.50	31.25	15.63	7.81	3.91
Absorbance 570nm	0.4213	0.7029	0.5601	0.7319	0.4407	0.4270	0.4357	0.5889	0.6605	0.6873	0.6929	0.6916
	0.4246	0.6978	0.5420	0.7072	0.4430	0.4295	0.4410	0.5324	0.6616	0.6898	0.6905	0.6930
Absorbance 600nm	0.5432	0.2123	0.4377	0.2369	0.5633	0.5444	0.5402	0.4000	0.2738	0.2240	0.2215	0.2114
	0.5484	0.2123	0.4406	0.2653	0.5660	0.5473	0.5594	0.4546	0.3094	0.2385	0.2199	0.2113
Absorbance difference	-0.1219	0.4906	0.1224	0.4950	-0.1226	-0.1174	-0.1045	0.1889	0.3867	0.4633	0.4714	0.4802
	-0.1238	0.4855	0.1014	0.4419	-0.1230	-0.1178	-0.1184	0.0778	0.3522	0.4513	0.4706	0.4817
% live cell		103.75	41.48	104.49	0.04	0.92	3.10	52.72	86.17	99.13	100.50	101.99
		102.88	37.92	95.51	-0.03	0.85	0.75	33.93	80.34	97.10	100.36	102.24
Average % live cell		103.31	39.70	100.00	0.01	0.89	1.93	43.33	83.26	98.11	100.43	102.11
SEM		0.43	1.78	4.49	0.03	0.03	1.18	9.39	2.92	1.01	0.07	0.13
Normalize data		103.31	39.70	100.00	0.01	0.89	1.93	43.33	83.26	98.11	100.43	102.11

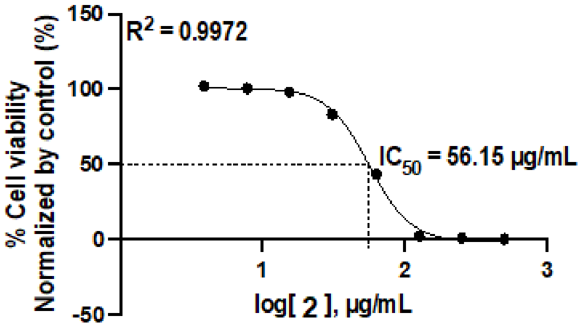

Media	Media +Cell	Cisplatin	Solvent	Sample concentrations (µg/mL)							
				300	150,00	75,00	37,50	18,75	9,38	4,69	2,34

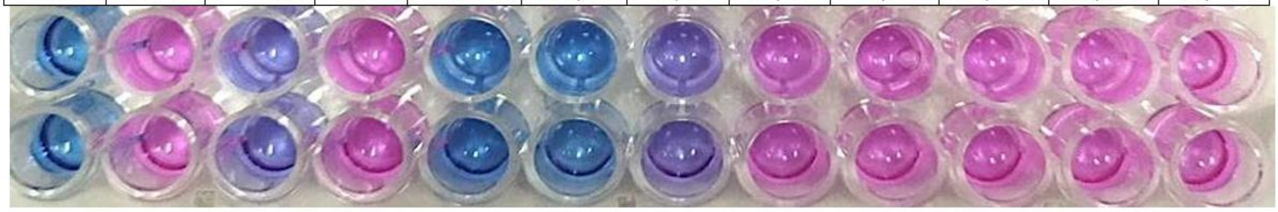

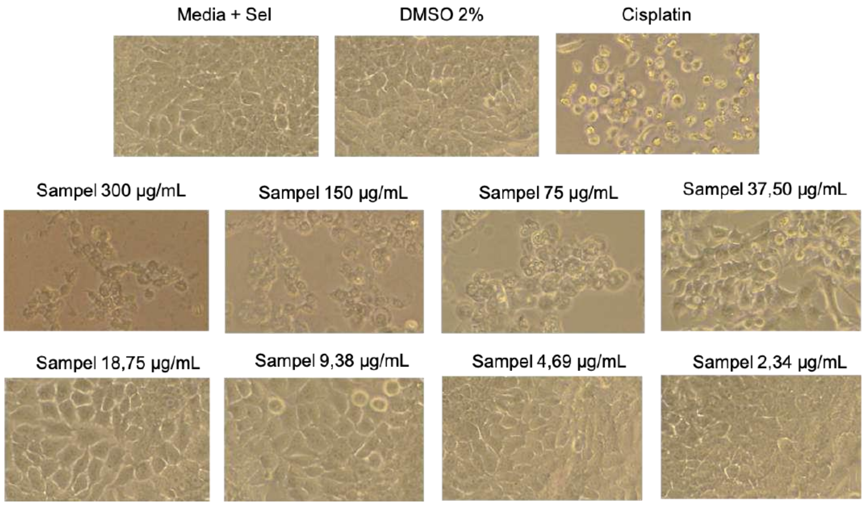

MCF-7 cell morphology at each concentration of compound **2**

Figure S40. Results of cytotoxic activity of **3** against MCF-7 cell line.

	Media	Media+Cell	Cisplatin	DMSO 2,00%	Sample concentrations (µg/mL)							
					500.00	250.00	125.00	62.50	31.25	15.63	7.81	3.91
Absorbance 570nm	0.4184	0.7361	0.5164	0.7271	0.5020	0.4571	0.6659	0.7166	0.7301	0.7249	0.6999	0.7463
	0.4279	0.7269	0.5190	0.7316	0.4924	0.4761	0.6329	0.7409	0.7095	0.7233	0.7160	0.7042
Absorbance 600nm	0.5337	0.1803	0.4928	0.1876	0.6193	0.5687	0.3574	0.2605	0.2086	0.1863	0.1748	0.1904
	0.5457	0.1778	0.4769	0.1834	0.6024	0.5620	0.3396	0.2262	0.1871	0.1830	0.1785	0.1746
Absorbance difference	-0.1153	0.5558	0.0236	0.5395	-0.1173	-0.1116	0.3085	0.4561	0.5215	0.5386	0.5251	0.5559
	-0.1178	0.5491	0.0421	0.5482	-0.1100	-0.0859	0.2933	0.5147	0.5224	0.5403	0.5375	0.5296
% live cell		101.81	21.22	99.34	-0.11	0.75	64.36	86.71	96.62	99.21	97.16	101.82
		100.79	24.02	100.66	0.99	4.64	62.06	95.59	96.75	99.46	99.04	97.84
Average % live cell		101.30	22.62	100.00	0.44	2.70	63.21	91.15	96.68	99.33	98.10	99.83
SEM		0.51	1.40	0.66	0.55	1.95	1.15	4.44	0.07	0.13	0.94	1.99
Normalize data		101.30	22.62	100.00	0.44	2.70	63.21	91.15	96.68	99.33	98.10	99.83

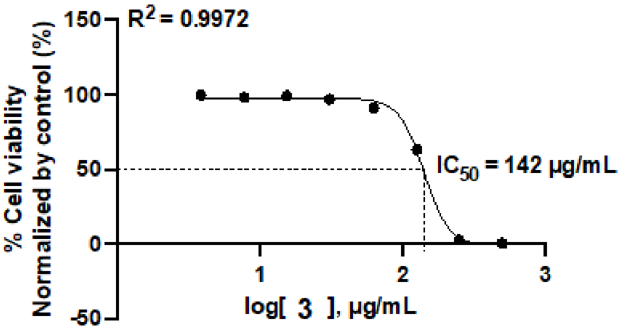

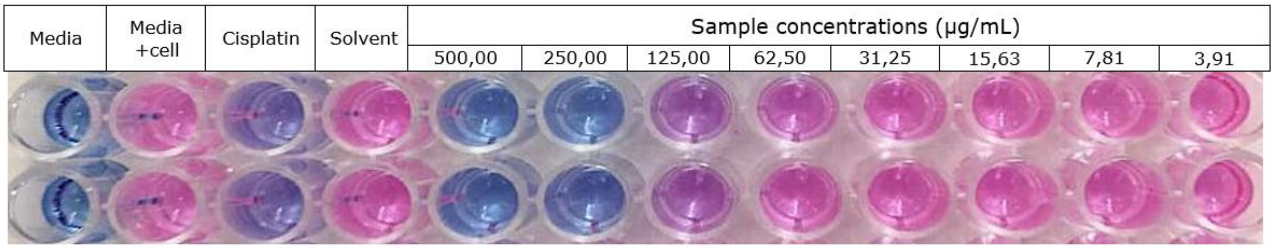

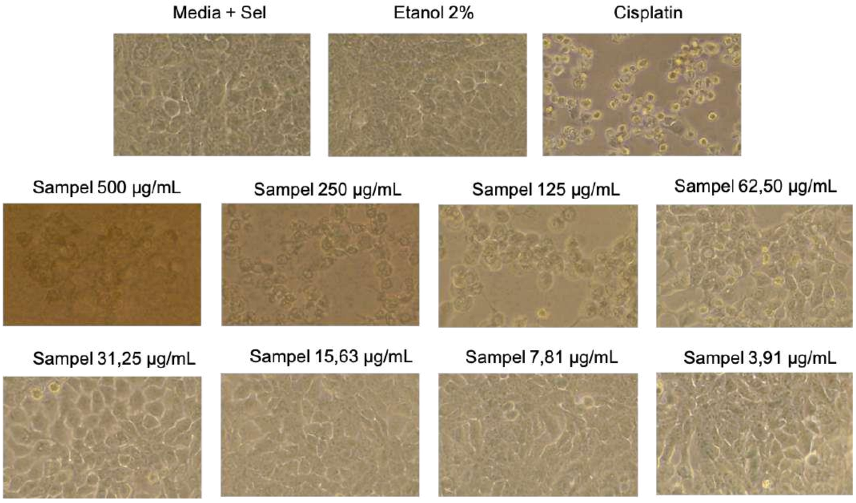

MCF-7 cell morphology at each concentration of compound **3**

Figure S41. Results of cytotoxic activity of **4** against MCF-7 cell line.

	Media	Media+Cell	Cisplatin	DMSO 2,00%	Sample concentrations (μg/mL)							
					500.00	250.00	125.00	62.50	31.25	15.63	7.81	3.91
Absorbance 570nm	0.4267	0.7354	0.546	0.7267	0.6523	0.7451	0.7266	0.7583	0.7172	0.7282	0.7315	0.7167
	0.4305	0.735	0.5434	0.7386	0.6757	0.7484	0.7262	0.7227	0.7275	0.7218	0.7063	0.728
Absorbance 600nm	0.5443	0.1818	0.4777	0.1825	0.381	0.2304	0.2009	0.1998	0.1857	0.1819	0.1819	0.1786
	0.5485	0.1815	0.4632	0.1828	0.3633	0.2174	0.1945	0.1841	0.1883	0.1798	0.1774	0.1821
Absorbance difference	-0.1176	0.5536	0.0683	0.5442	0.2713	0.5147	0.5257	0.5585	0.5315	0.5463	0.5496	0.5381
	-0.1180	0.5535	0.0802	0.5558	0.3124	0.5310	0.5317	0.5386	0.5392	0.5420	0.5289	0.5459
% live cell		100.54	27.87	99.13	58.27	94.71	96.36	101.27	97.23	99.45	99.94	98.22
		100.52	29.65	100.87	64.42	97.15	97.26	98.29	98.38	98.80	96.84	99.39
Average % live cell		100.53	28.76	100.00	61.34	95.93	96.81	99.78	97.81	99.12	98.39	98.80
SEM		0.01	0.89	0.87	3.08	1.22	0.45	1.49	0.58	0.32	1.55	0.58
Normalize data		100.53	28.76	100.00	61.34	95.93	96.81	99.78	97.81	99.12	98.39	98.80

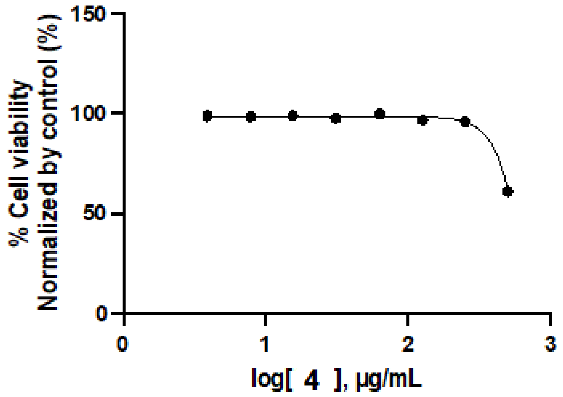

Media	Media +cell	Cisplatin	Solvent	Sample concentrations (μg/mL)							
				500,00	250,00	125,00	62,50	31,25	15,63	7,81	3,91

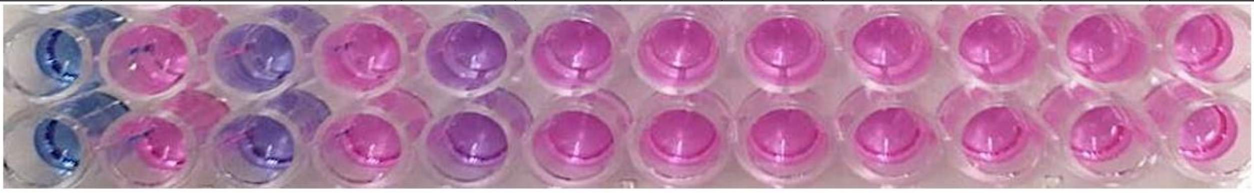

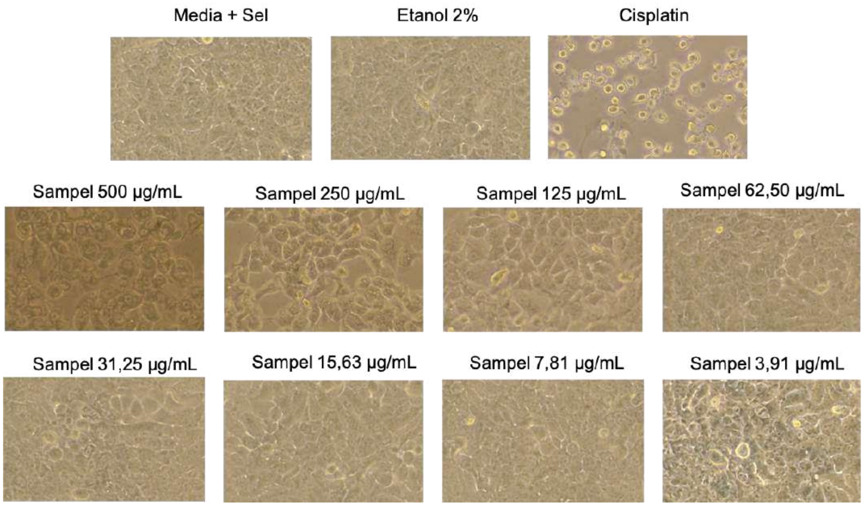

MCF-7 cell morphology at each concentration of compound **4**

Figure S42. Results of cytotoxic activity of **5** against MCF-7 cell line.

	Media	Media + cell	Cisplatin	Solvent	Sample concentrations (µg/mL)							
					2,34	4,69	9,38	18,75	37,50	75,00	150,00	300,00
Absorbance 570nm	0,4706	0,7728	0,5746	0,7568	0,7833	0,7678	0,7578	0,7323	0,7316	0,5663	0,4709	0,4640
	0,4842	0,7666	0,5718	0,7562	0,7754	0,7728	0,7657	0,7419	0,7088	0,5703	0,4761	0,5014
Absorbance 600nm	0,6023	0,2352	0,5244	0,2838	0,2467	0,2531	0,2642	0,2845	0,3246	0,5133	0,5965	0,5893
	0,6201	0,2342	0,5277	0,2782	0,2450	0,2531	0,2723	0,3026	0,3629	0,5189	0,6023	0,6325
Absorbance difference	-0,1317	0,5376	0,0502	0,4730	0,5366	0,5147	0,4936	0,4478	0,4070	0,0530	-0,1256	-0,1253
	-0,1359	0,5324	0,0441	0,4780	0,5304	0,5197	0,4934	0,4393	0,3459	0,0514	-0,1262	-0,1311
% live cell		110,19	30,20	99,59	110,03	106,43	102,97	95,45	88,76	30,66	1,35	1,40
		109,34	29,20	100,41	109,01	107,25	102,94	94,06	78,73	30,40	1,25	0,44
Average % live cell		109,77	29,70	100,00	109,52	106,84	102,95	94,76	83,74	30,53	1,30	0,92
SEM		0,43	0,50	0,41	0,51	0,41	0,02	0,70	5,01	0,13	0,05	0,48
Normalized data % live cell		109,77	29,70	100,00	109,52	106,84	102,95	94,76	83,74	30,53	1,30	0,92

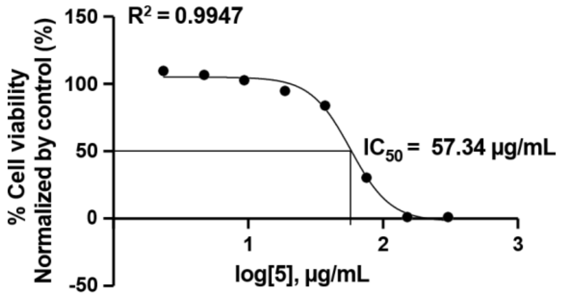

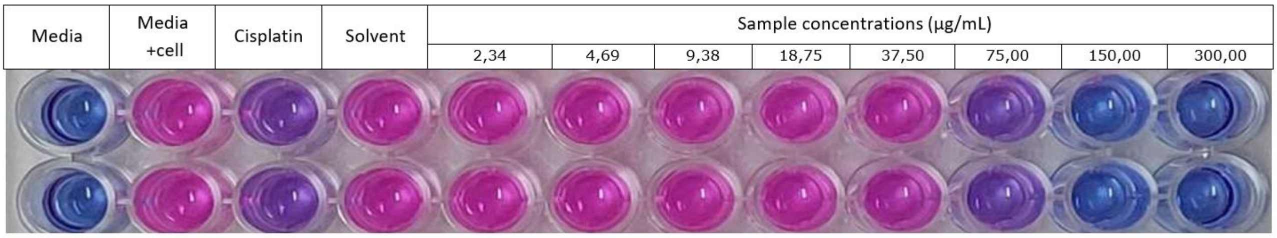

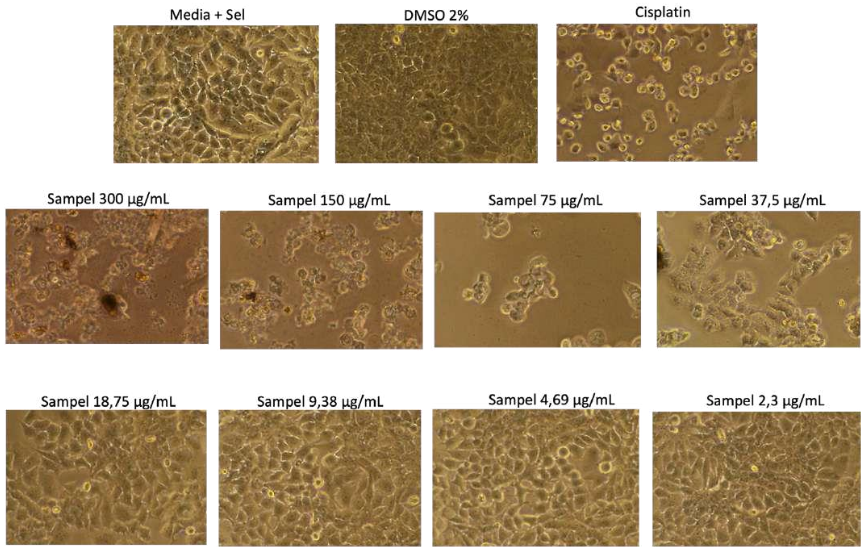

MCF-7 cell morphology at each concentration of compound **5**

Figure S43. Results of cytotoxic activity of **6** against MCF-7 cell line.

	Media	Media + cell	Cisplatin	Solvent	Sample concentrations (µg/mL)							
					2,34	4,69	9,38	18,75	37,50	75,00	150,00	300,00
Absorbance 570nm	0,4756	0,7769	0,5661	0,7535	0,7673	0,7584	0,7799	0,7670	0,7051	0,6611	0,6010	0,5074
	0,4961	0,7783	0,5699	0,7509	0,7678	0,7539	0,7754	0,7505	0,5997	0,6806	0,6265	0,4938
Absorbance 600nm	0,6079	0,2388	0,5239	0,2793	0,2381	0,2313	0,2517	0,2676	0,2935	0,4269	0,5192	0,6099
	0,6319	0,2364	0,5260	0,3143	0,2452	0,2485	0,2603	0,2644	0,2166	0,4299	0,5100	0,6108
Absorbance difference	-0,1323	0,5381	0,0422	0,4742	0,5292	0,5271	0,5282	0,4994	0,4116	0,2342	0,0818	-0,1025
	-0,1358	0,5419	0,0439	0,4366	0,5226	0,5054	0,5151	0,4861	0,3831	0,2507	0,1165	-0,1170
% live cell		114,03	29,90	103,19	112,52	112,16	112,35	107,46	92,57	62,47	36,62	5,35
		114,67	30,19	96,81	111,40	108,48	110,13	105,21	87,73	65,27	42,51	2,89
Average % live cell		114,35	30,04	100,00	111,96	110,32	111,24	106,34	90,15	63,87	39,56	4,12
SEM		0,32	0,14	3,19	0,56	1,84	1,11	1,13	2,42	1,40	2,94	1,23
Normalized data % live cell		114,35	30,04	100,00	111,96	110,32	111,24	106,34	90,15	63,87	39,56	4,12

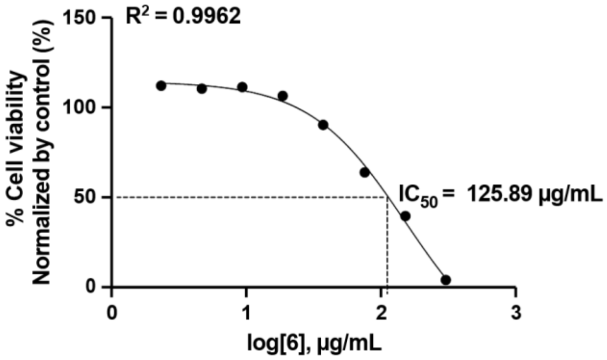

Media	Media +cell	Cisplatin	Solvent	Sample concentrations (µg/mL)							
				2,34	4,69	9,38	18,75	37,50	75,00	150,00	300,00

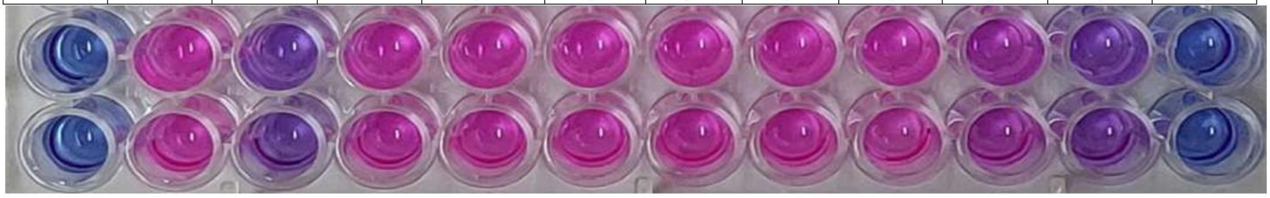

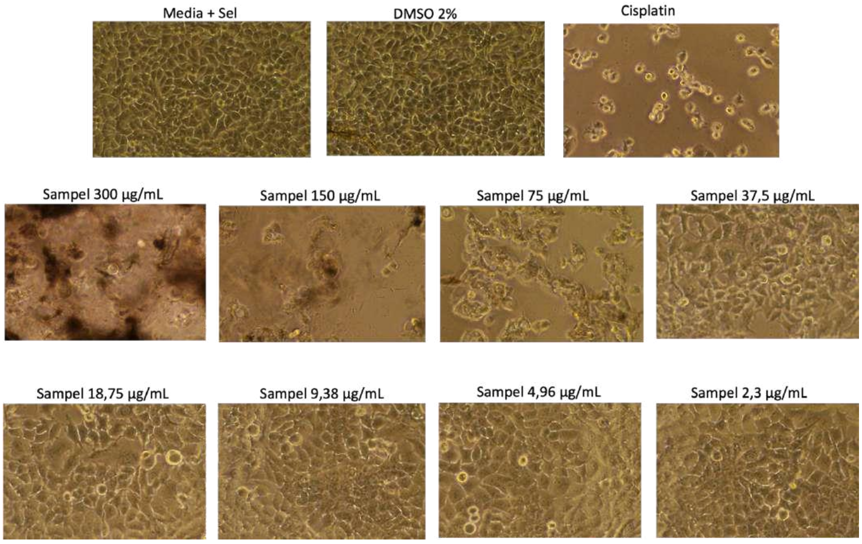

MCF-7 cell morphology at each concentration of compound **6**

Figure S44. Results of cytotoxic activity of **7** against MCF-7 cell line.

	Media	Media + cell	Cisplatin	Solvent	Sample concentrations (μg/mL)							
					2,34	4,69	9,38	18,75	37,50	75,00	150,00	300,00
Absorbance 570nm	0,4693	0,7481	0,5716	0,7565	0,7825	0,7779	0,7556	0,7436	0,7284	0,6887	0,5271	0,4850
	0,4886	0,7770	0,5703	0,7580	0,7592	0,7694	0,7567	0,7374	0,7153	0,7010	0,5306	0,4951
Absorbance 600nm	0,6010	0,2242	0,5216	0,2728	0,2356	0,2420	0,2364	0,2434	0,2978	0,3639	0,5485	0,6092
	0,6225	0,2337	0,5218	0,2929	0,2314	0,2465	0,2318	0,2723	0,3080	0,3872	0,5937	0,6355
Absorbance difference	-0,1317	0,5239	0,0500	0,4837	0,5469	0,5359	0,5192	0,5002	0,4306	0,3248	-0,0214	-0,1242
	-0,1339	0,5433	0,0485	0,4651	0,5278	0,5229	0,5249	0,4651	0,4073	0,3138	-0,0631	-0,1404
% live cell		108,15	30,11	101,53	111,94	110,13	107,38	104,25	92,79	75,36	18,35	1,42
		111,35	29,86	98,47	108,79	107,99	108,32	98,47	88,95	73,55	11,48	-1,25
Average % live cell		109,75	29,98	100,00	110,37	109,06	107,85	101,36	90,87	74,46	14,91	0,08
SEM		1,60	0,12	1,53	1,57	1,07	0,47	2,89	1,92	0,91	3,43	1,33
Normalized data % live cell		109,75	29,98	100,00	110,37	109,06	107,85	101,36	90,87	74,46	14,91	0,08

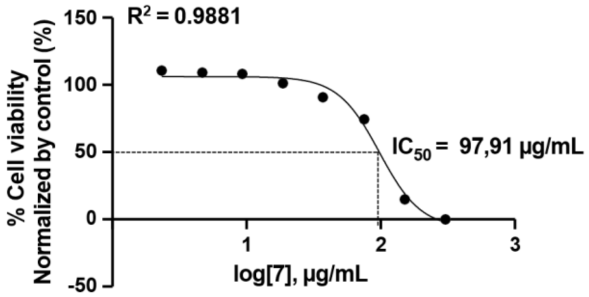

Media	Media +cell	Cisplatin	Solvent	Sample concentrations (μg/mL)							
				2,34	4,69	9,38	18,75	37,50	75,00	150,00	300,00

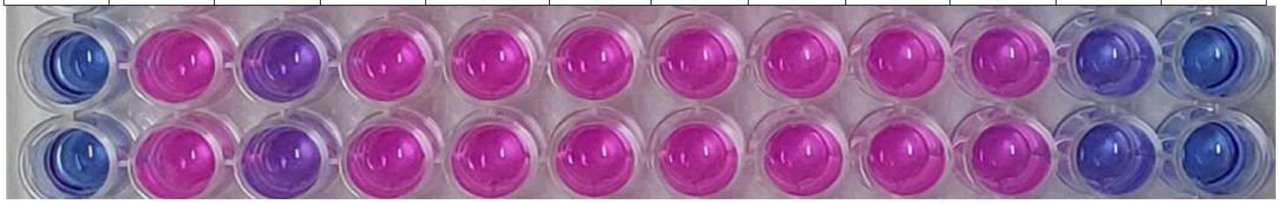

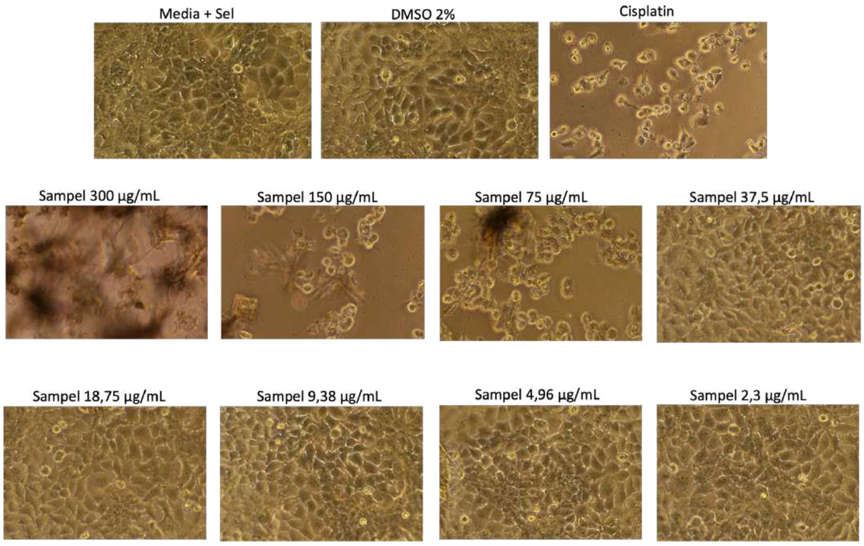

MCF-7 cell morphology at each concentration of compound **7**

Figure S45. Results of cytotoxic activity of **1** against B16-F10 cell line.

	Media	Media + cell	Cisplatin	Solvent	Sample concentrations (µg/mL)							
					2,34	4,69	9,38	18,75	37,50	75,00	150,00	300,00
Absorbance 570nm	0,4740	0,8303	0,5658	0,7732	0,8008	0,8332	0,8115	0,7873	0,7135	0,5146	0,4995	0,5000
	0,4868	0,7935	0,5606	0,7750	0,8084	0,8181	0,8023	0,7869	0,7186	0,5299	0,5090	0,4896
Absorbance 600nm	0,5998	0,2586	0,5273	0,2734	0,2661	0,2622	0,2513	0,2510	0,3629	0,5810	0,6237	0,6243
	0,6178	0,2801	0,5600	0,2767	0,2595	0,2602	0,2566	0,2472	0,3699	0,6018	0,6376	0,6129
Absorbance difference	-0,1258	0,5717	0,0385	0,4998	0,5347	0,5710	0,5602	0,5363	0,3506	-0,0664	-0,1242	-0,1243
	-0,1310	0,5134	0,0006	0,4983	0,5489	0,5579	0,5457	0,5397	0,3487	-0,0719	-0,1286	-0,1233
% live cell		111,58	26,60	100,12	105,68	111,47	109,75	105,94	76,34	9,88	0,67	0,65
		102,29	20,56	99,88	107,94	109,38	107,43	106,48	76,04	9,00	-0,03	0,81
Average % live cell		106,93	23,58	100,00	106,81	110,42	108,59	106,21	76,19	9,44	0,32	0,73
SEM		4,65	3,02	0,12	1,13	1,04	1,16	0,27	0,15	0,44	0,35	0,08
Normalized data % live cell		106,93	23,58	100,00	106,81	110,42	108,59	106,21	76,19	9,44	0,32	0,73

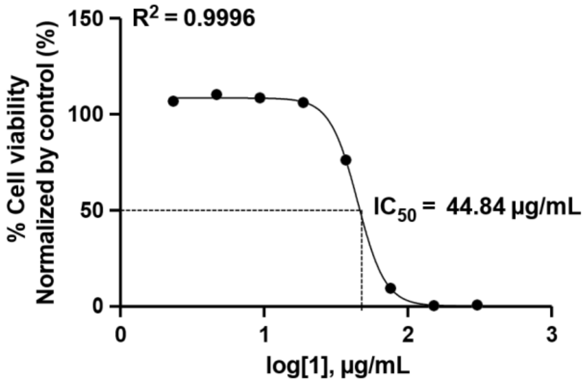

Media	Media +cell	Cisplatin	Solvent	Sample concentrations (µg/mL)							
				2,34	4,69	9,38	18,75	37,50	75,00	150,00	300,00

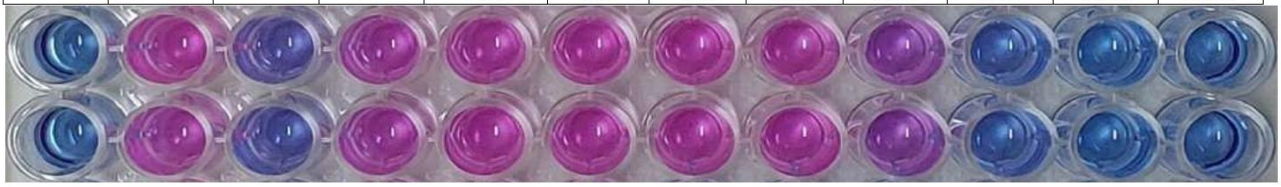

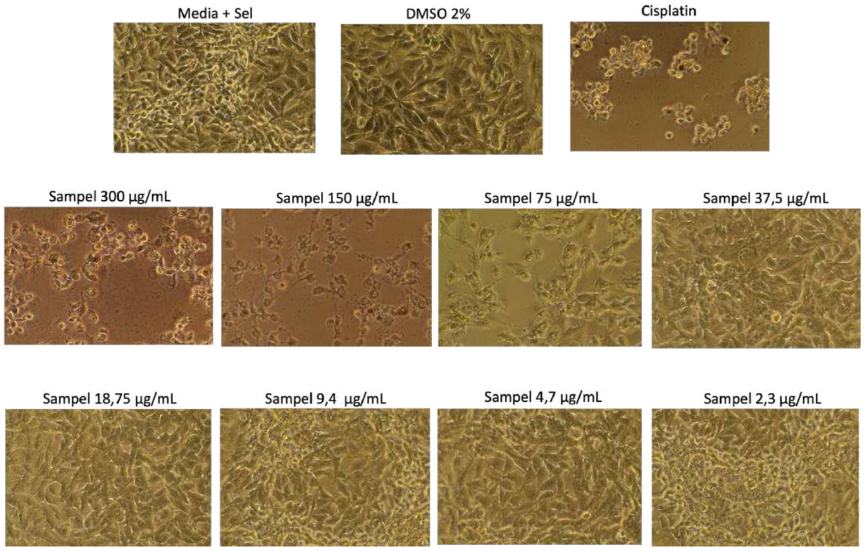

B16-F10 cell morphology at each concentration of compound **1**

Figure S46. Results of cytotoxic activity of **2** against B16-F10 cell line.

	Media	Media + cell	Cisplatin	Solvent	Sample concentrations (μg/mL)							
					2,34	4,69	9,38	18,75	37,50	75,00	150,00	300,00
Absorbance 570nm	0,3298	0,5262	0,3541	0,5074	0,5555	0,5501	0,5502	0,5308	0,5103	0,3734	0,3537	0,3510
	0,3429	0,5442	0,3610	0,5103	0,5432	0,5559	0,5944	0,5297	0,5093	0,4019	0,3661	0,3515
Absorbance 600nm	0,3880	0,2086	0,4036	0,2196	0,2112	0,2087	0,2053	0,1906	0,2217	0,3771	0,4089	0,4047
	0,4026	0,2030	0,4009	0,2184	0,2110	0,2072	0,2150	0,1918	0,2098	0,3695	0,4185	0,4017
Absorbance difference	-0,0582	0,3176	-0,0495	0,2878	0,3443	0,3414	0,3449	0,3402	0,2886	-0,0037	-0,0552	-0,0537
	-0,0597	0,3412	-0,0399	0,2919	0,3322	0,3487	0,3794	0,3379	0,2995	0,0324	-0,0524	-0,0502
% live cell		107,96	2,71	99,41	115,61	114,78	115,78	114,44	99,64	15,84	1,08	1,51
		114,72	5,46	100,59	112,14	116,87	125,67	113,78	102,77	26,19	1,88	2,51
Average % live cell		111,34	4,09	100,00	113,88	115,83	120,73	114,11	101,20	21,01	1,48	2,01
SEM		3,38	1,38	0,59	1,73	1,05	4,95	0,33	1,56	5,17	0,40	0,50
Normalized data % live cell		111,34	4,09	100,00	113,88	115,83	120,73	114,11	101,20	21,01	1,48	2,01

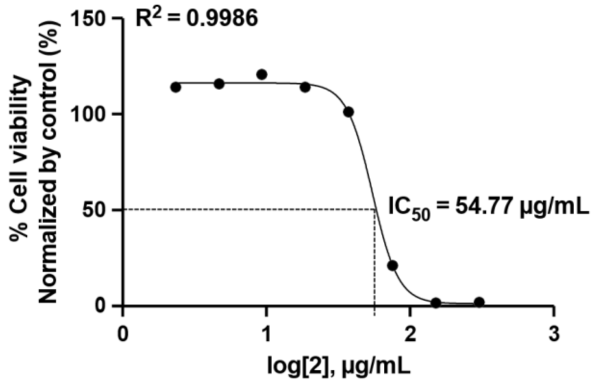

Media	Media +cell	Cisplatin	Solvent	Sample concentrations (μg/mL)							
				2,34	4,69	9,38	18,75	37,50	75,00	150,00	300,00

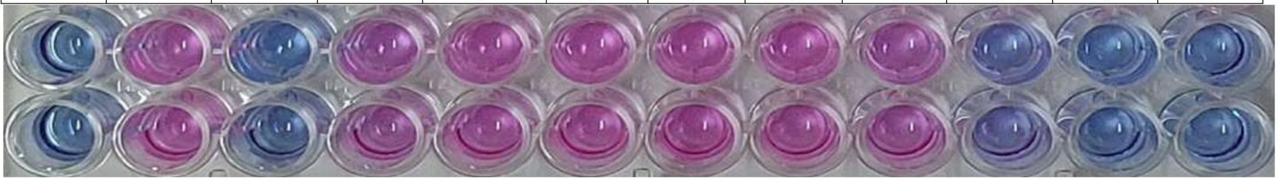

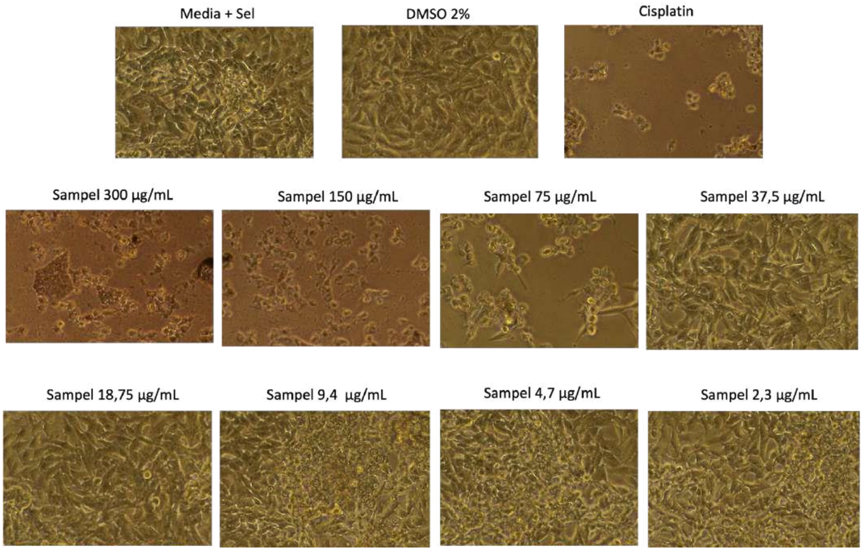

B16-F10 cell morphology at each concentration of compound **2**

Figure S47. Results of cytotoxic activity of **3** against B16-F10 cell line.

	Media	Media + cell	Cisplatin	Solvent	Sample concentrations (µg/mL)							
					2,34	4,69	9,38	18,75	37,50	75,00	150,00	300,00
Absorbance 570nm	0,4350	0,7556	0,6233	0,7508	0,7565	0,7121	0,7175	0,7637	0,7275	0,4970	0,5186	0,5010
	0,4505	0,7470	0,5993	0,7526	0,7005	0,7952	0,7523	0,7182	0,7569	0,4965	0,5574	0,4966
Absorbance 600nm	0,5490	0,2919	0,4025	0,2771	0,3405	0,3734	0,3831	0,3505	0,4008	0,6142	0,6433	0,6308
	0,5662	0,2662	0,4239	0,2642	0,3929	0,3201	0,3289	0,3634	0,3690	0,6165	0,6626	0,6316
Absorbance difference	-0,1140	0,4637	0,2208	0,4737	0,4160	0,3387	0,3344	0,4132	0,3267	-0,1172	-0,1247	-0,1298
	-0,1157	0,4808	0,1754	0,4884	0,3076	0,4751	0,4234	0,3548	0,3879	-0,1200	-0,1052	-0,1350
% live cell		98,54	57,17	100,25	90,42	77,25	76,52	89,94	75,21	-0,40	-1,68	-2,55
		101,46	49,44	102,75	71,96	100,49	91,68	79,99	85,63	-0,88	1,64	-3,43
Average % live cell		100,00	53,30	101,50	81,19	88,87	84,10	84,97	80,42	-0,64	-0,02	-2,99
SEM		1,46	3,87	1,25	9,23	11,62	7,58	4,97	5,21	0,24	1,66	0,44
Normalized data % live cell		98,52	52,52	100,00	79,99	87,56	82,86	83,71	79,23	-0,63	-0,02	-2,95

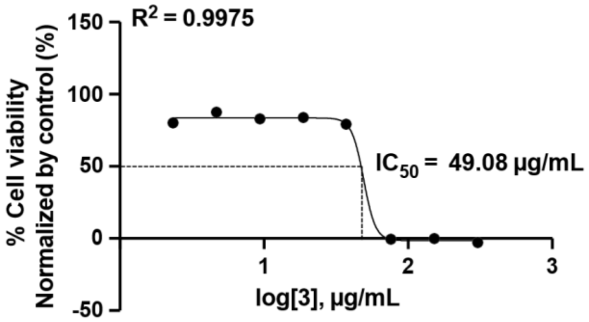

Media	Media +cell	Cisplatin	Solvent	Sample concentrations (µg/mL)							
				2,34	4,69	9,38	18,75	37,50	75,00	150,00	300,00

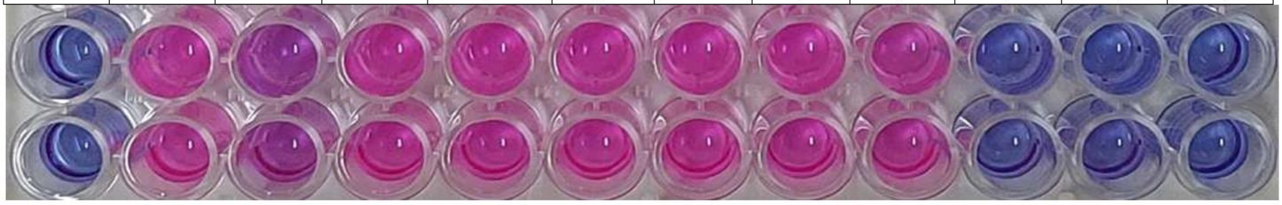

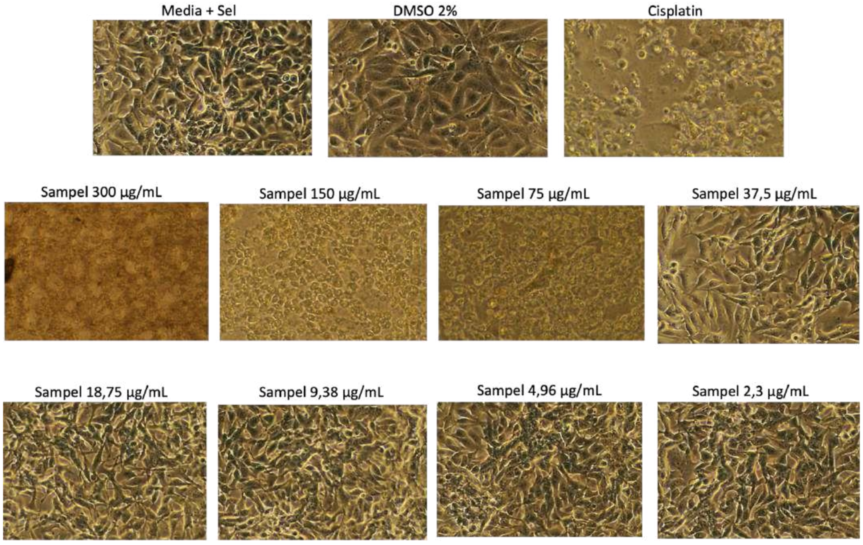

B16-F10 cell morphology at each concentration of compound **3**

Figure S48. Results of cytotoxic activity of **4** against B16-F10 cell line.

	Media	Media + cell	Cisplatin	Solvent	Sample concentrations (µg/mL)							
					2,34	4,69	9,38	18,75	37,50	75,00	150,00	300,00
Absorbance 570nm	0,4350	0,7556	0,6233	0,7508	0,7646	0,6342	0,6616	0,7139	0,7212	0,7322	0,7312	0,5145
	0,4505	0,7470	0,5993	0,7526	0,6985	0,6452	0,6456	0,7058	0,691	0,7509	0,7442	0,5188
Absorbance 600nm	0,5490	0,2919	0,4025	0,2771	0,3509	0,4202	0,419	0,3901	0,3664	0,362	0,3704	0,6063
	0,5662	0,2662	0,4239	0,2642	0,4006	0,4604	0,4365	0,3942	0,4241	0,3878	0,3356	0,5804
Absorbance difference	-0,1140	0,4637	0,2208	0,4737	0,4137	0,2140	0,2426	0,3238	0,3548	0,3702	0,3608	-0,0918
	-0,1157	0,4808	0,1754	0,4884	0,2979	0,1848	0,2091	0,3116	0,2669	0,3631	0,4086	-0,0616
% live cell		98,54	57,17	100,25	90,03	56,01	60,88	74,71	79,99	82,62	81,02	3,93
		101,46	49,44	102,75	70,30	51,04	55,18	72,64	65,02	81,41	89,16	9,07
Average % live cell		100,00	53,30	101,50	80,17	53,53	58,03	73,68	72,51	82,01	85,09	6,50
SEM		1,46	3,87	1,25	9,86	2,49	2,85	1,04	7,49	0,60	4,07	2,57
Normalized data % live cell		98,52	52,52	100,00	78,98	52,74	57,17	72,59	71,44	80,80	83,83	6,40

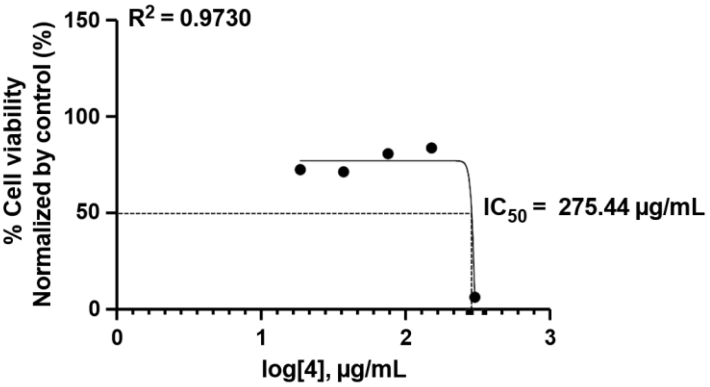

Media	Media +cell	Cisplatin	Solvent	Sample concentrations (µg/mL)							
				2,34	4,69	9,38	18,75	37,50	75,00	150,00	300,00

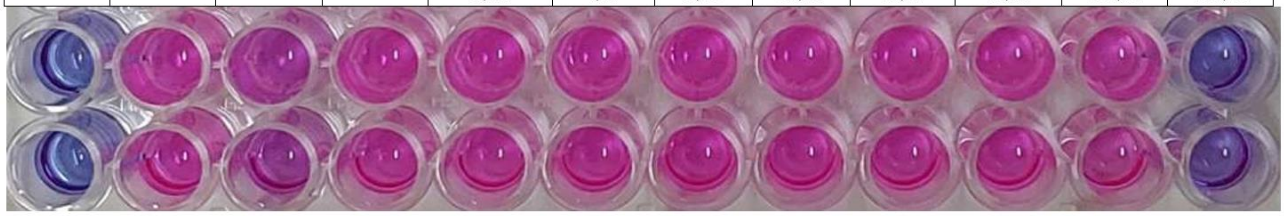

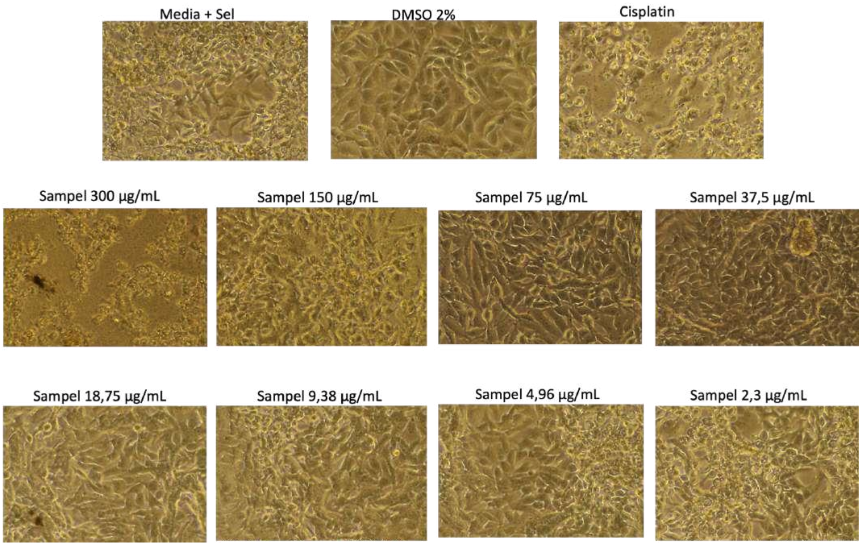

B16-F10 cell morphology at each concentration of compound **4**

Figure S49. Results of cytotoxic activity of **5** against B16-F10 cell line.

	Media	Media + cell	Cisplatin	Solvent	Sample concentrations (µg/mL)							
					2,34	4,69	9,38	18,75	37,50	75,00	150,00	300,00
Absorbance 570nm	0,4525	0,7797	0,4912	0,7199	0,7613	0,7580	0,7566	0,7478	0,6946	0,5090	0,4848	0,4863
	0,4987	0,7640	0,4970	0,7186	0,7608	0,7664	0,7653	0,7614	0,6989	0,5149	0,4963	0,4848
Absorbance 600nm	0,5775	0,2607	0,5922	0,3137	0,2741	0,2910	0,2879	0,2816	0,3693	0,6003	0,6215	0,6162
	0,6378	0,2726	0,5976	0,3261	0,2911	0,2927	0,2925	0,2987	0,3842	0,6199	0,6250	0,6166
Absorbance difference	-0,1250	0,5190	-0,1010	0,4062	0,4872	0,4670	0,4687	0,4662	0,3253	-0,0913	-0,1367	-0,1299
	-0,1391	0,4914	-0,1006	0,3925	0,4697	0,4737	0,4728	0,4627	0,3147	-0,1050	-0,1287	-0,1318
% live cell		122,52	5,84	101,29	116,53	112,73	113,05	112,58	86,07	7,67	-0,88	0,40
		117,32	5,92	98,71	113,24	113,99	113,82	111,92	84,07	5,09	0,63	0,05
Average % live cell		119,92	5,88	100,00	114,89	113,36	113,44	112,25	85,07	6,38	-0,12	0,23
SEM		2,60	0,04	1,29	1,65	0,63	0,39	0,33	1,00	1,29	0,75	0,18
Normalized data % live cell		119,92	5,88	100,00	114,89	113,36	113,44	112,25	85,07	6,38	-0,12	0,23

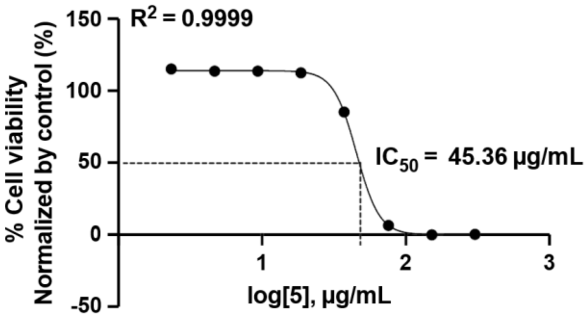

Media	Media +cell	Cisplatin	Solvent	Sample concentrations (µg/mL)							
				2,34	4,69	9,38	18,75	37,50	75,00	150,00	300,00

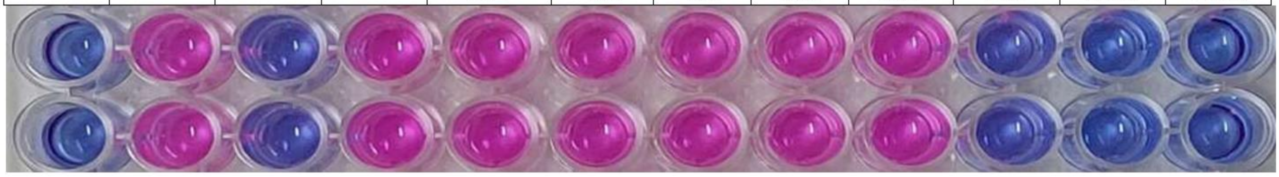

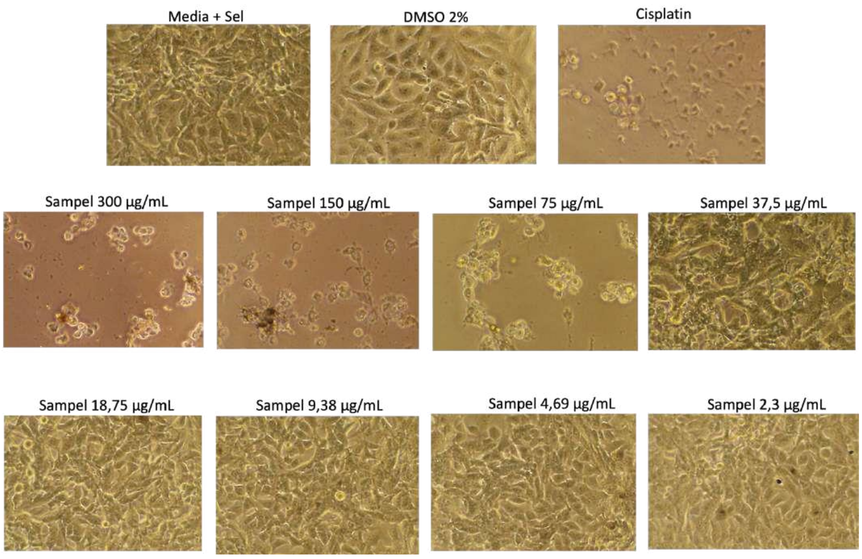

B16-F10 cell morphology at each concentration of compound **5**

Figure S50. Results of cytotoxic activity of **6** against B16-F10 cell line.

	Media	Media + cell	Cisplatin	Solvent	Sample concentrations (µg/mL)							
					2,34	4,69	9,38	18,75	37,50	75,00	150,00	300,00
Absorbance 570nm	0,4927	0,7869	0,4960	0,7199	0,7273	0,7219	0,7350	0,7306	0,6836	0,6445	0,5961	0,5731
	0,4921	0,7905	0,5134	0,7378	0,7850	0,7901	0,7923	0,7715	0,7871	0,7280	0,6339	0,4870
Absorbance 600nm	0,6282	0,2574	0,5898	0,3211	0,3149	0,3237	0,3074	0,3286	0,3757	0,4500	0,5443	0,6970
	0,6261	0,2592	0,6123	0,3165	0,2735	0,2776	0,2765	0,2697	0,3101	0,3754	0,4843	0,6138
Absorbance difference	-0,1355	0,5295	-0,0938	0,3988	0,4124	0,3982	0,4276	0,4020	0,3079	0,1945	0,0518	-0,1239
	-0,1340	0,5313	-0,0989	0,4213	0,5115	0,5125	0,5158	0,5018	0,4770	0,3526	0,1496	-0,1268
% live cell		121,93	7,52	97,94	100,43	97,82	103,22	98,52	81,25	60,44	34,24	1,99
		122,26	6,58	102,06	118,62	118,81	119,41	116,84	112,29	89,45	52,19	1,46
Average % live cell		122,09	7,05	100,00	109,53	108,31	111,32	107,68	96,77	74,94	43,22	1,73
SEM		0,17	0,47	2,06	9,10	10,49	8,09	9,16	15,52	14,51	8,98	0,27
Normalized data % live cell		122,09	7,05	100,00	109,53	108,31	111,32	107,68	96,77	74,94	43,22	1,73

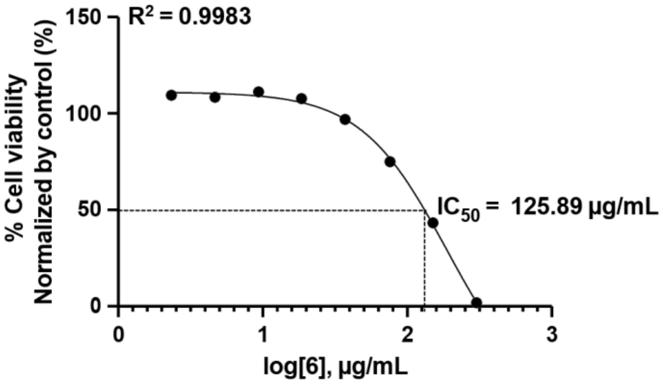

Media	Media +cell	Cisplatin	Solvent	Sample concentrations (µg/mL)							
				2,34	4,69	9,38	18,75	37,50	75,00	150,00	300,00

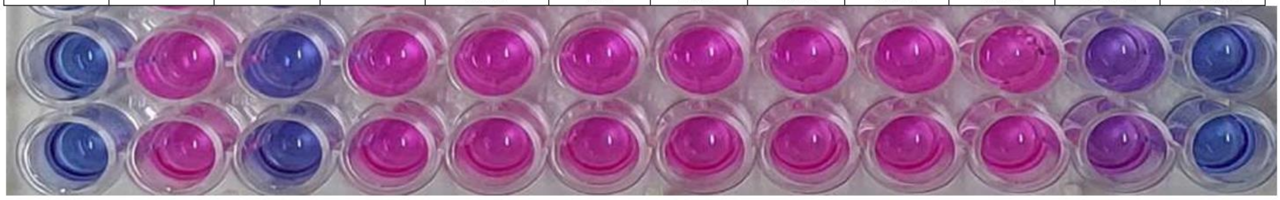

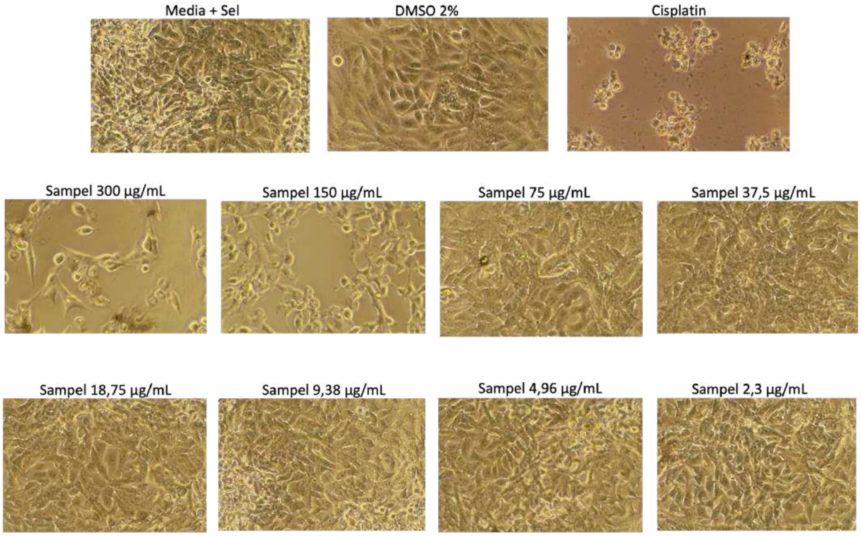

B16-F10 cell morphology at each concentration of compound **6**

Figure S51. Results of cytotoxic activity of **7** against B16-F10 cell line.

	Media	Media + cell	Cisplatin	Solvent	Sample concentrations (µg/mL)							
					2,34	4,69	9,38	18,75	37,50	75,00	150,00	300,00
Absorbance 570nm	0,4836	0,7798	0,4930	0,7107	0,7595	0,7377	0,7650	0,7577	0,7595	0,6684	0,5573	0,5005
	0,4968	0,7865	0,5016	0,7147	0,7555	0,7494	0,7822	0,7613	0,7579	0,5643	0,5584	0,5098
Absorbance 600nm	0,6183	0,2650	0,5895	0,3311	0,2952	0,2814	0,2873	0,2813	0,2763	0,4048	0,5319	0,6177
	0,6344	0,2664	0,6046	0,3280	0,2906	0,2926	0,2830	0,2713	0,2857	0,5586	0,5180	0,6308
Absorbance difference	-0,1347	0,5148	-0,0965	0,3796	0,4643	0,4563	0,4777	0,4764	0,4832	0,2636	0,0254	-0,1172
	-0,1376	0,5201	-0,1030	0,3867	0,4649	0,4568	0,4992	0,4900	0,4722	0,0057	0,0404	-0,1210
% live cell		125,35	7,64	99,32	115,63	114,09	118,21	117,96	119,27	76,98	31,11	3,65
		126,37	6,38	100,68	115,74	114,18	122,35	120,58	117,15	27,32	34,00	2,92
Average % live cell		125,86	7,01	100,00	115,68	114,13	120,28	119,27	118,21	52,15	32,55	3,28
SEM		0,51	0,63	0,68	0,06	0,05	2,07	1,31	1,06	24,83	1,44	0,37
Normalized data % live cell		125,86	7,01	100,00	115,68	114,13	120,28	119,27	118,21	52,15	32,55	3,28

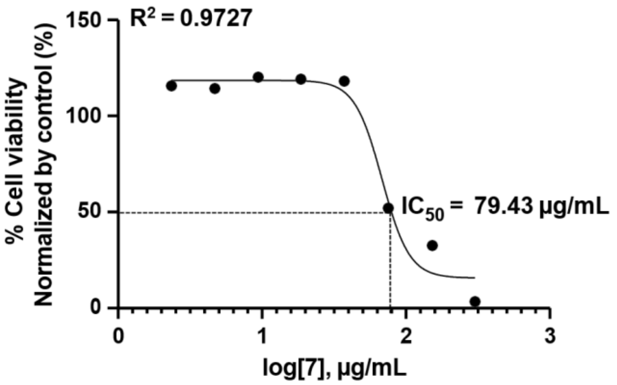

Media	Media +cell	Cisplatin	Solvent	Sample concentrations (µg/mL)							
				2,34	4,69	9,38	18,75	37,50	75,00	150,00	300,00

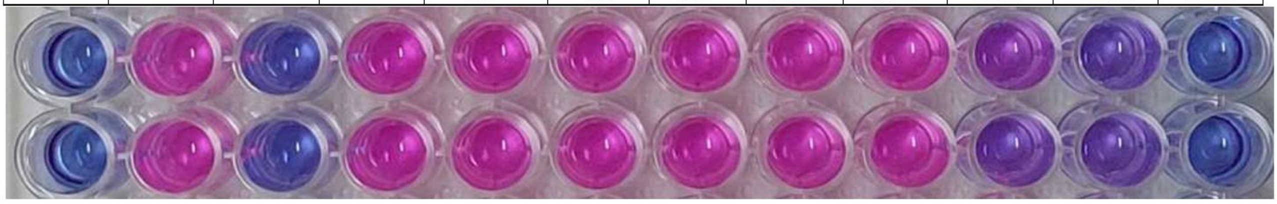

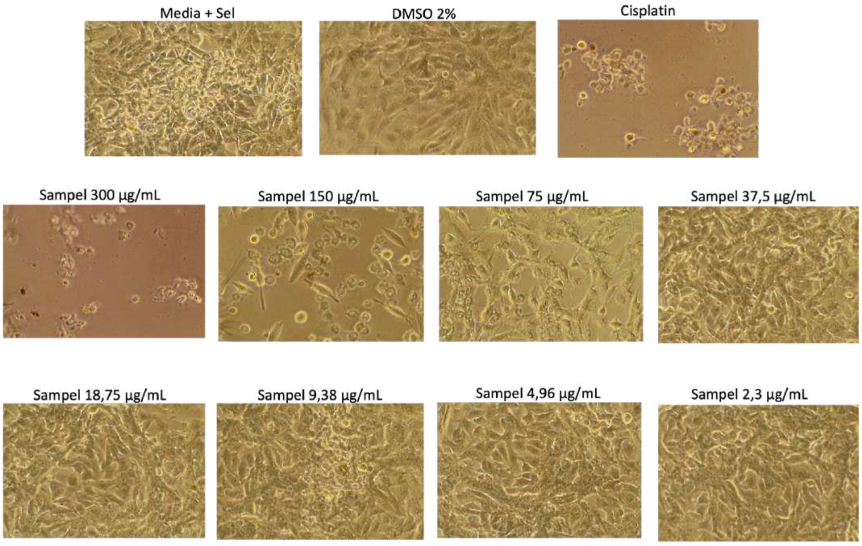

B16-F10 cell morphology at each concentration of compound **7**

Figure S52. Results of cytotoxic activity of **1** against CV-1 cell line.

	Media	Media + cell	Cisplatin	Solvent	Sample concentrations (µg/mL)							
					2,34	4,69	9,38	18,75	37,50	75,00	150,00	300,00
Absorbance 570nm	0,5356	0,7346	0,6104	0,7829	0,7258	0,7339	0,7347	0,7302	0,7241	0,6270	0,5508	0,4946
	0,5339	0,7388	0,6105	0,7838	0,7183	0,7412	0,7442	0,7478	0,7443	0,6362	0,5726	0,5070
Absorbance 600nm	0,5267	0,2567	0,4439	0,2193	0,2540	0,2521	0,2490	0,2560	0,2727	0,3454	0,4778	0,4896
	0,5317	0,2659	0,4321	0,2397	0,2553	0,2682	0,2621	0,2627	0,2914	0,3642	0,5062	0,5053
Absorbance difference	0,0089	0,4779	0,1665	0,5636	0,4718	0,4818	0,4857	0,4742	0,4514	0,2816	0,0730	0,0050
	0,0022	0,4729	0,1784	0,5441	0,4630	0,4730	0,4821	0,4851	0,4529	0,2720	0,0664	0,0017
% live cell		100,53	34,26	118,77	99,23	101,36	102,19	99,74	94,89	58,75	14,36	-0,12
		99,47	36,79	114,62	97,36	99,49	101,43	102,06	95,21	56,71	12,95	-0,82
Average % live cell		100,00	35,52	116,70	98,30	100,43	101,81	100,90	95,05	57,73	13,65	-0,47
SEM		0,53	1,27	2,08	0,94	0,94	0,38	1,16	0,16	1,02	0,70	0,35
Normalized data % live cell		85,69	30,44	100,00	84,23	86,06	87,24	86,47	81,45	49,47	11,70	-0,40

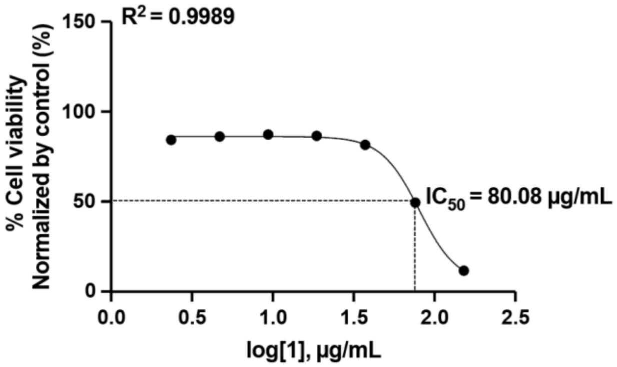

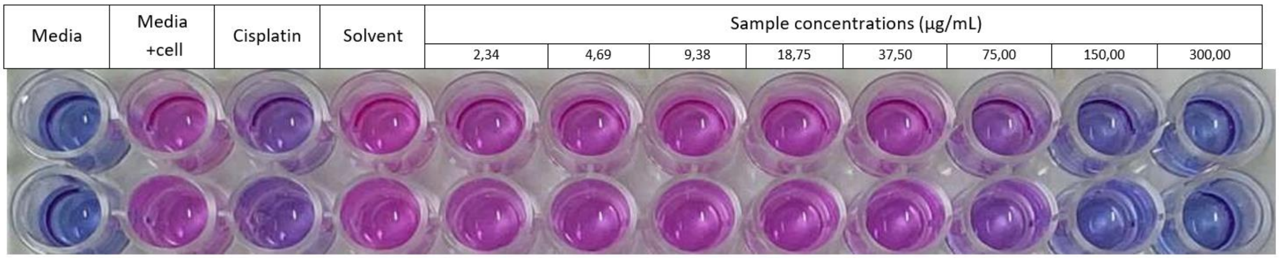

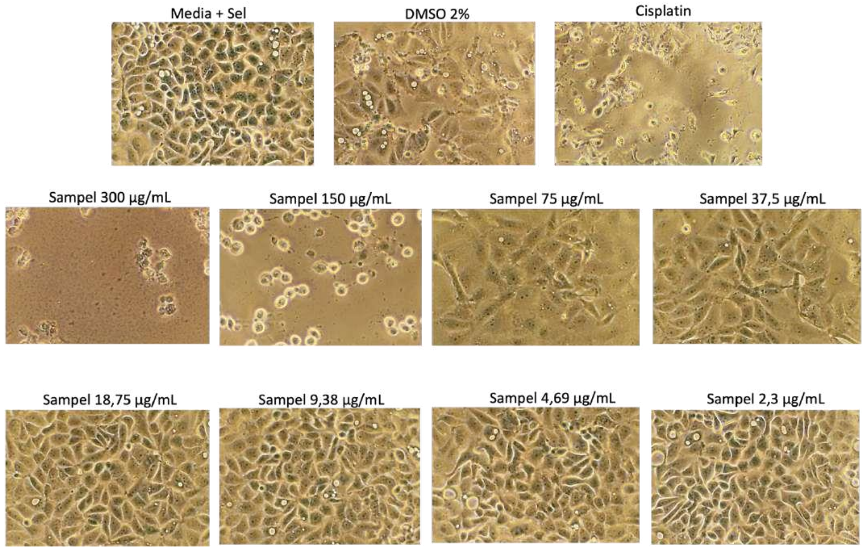

CV-1 cell morphology at each concentration of compound **1**

Figure S53. Results of cytotoxic activity of **2** against CV-1 cell line.

	Media	Media + cell	Cisplatin	Solvent	Sample concentrations (µg/mL)							
					2,34	4,69	9,38	18,75	37,50	75,00	150,00	300,00
Absorbance 570nm	0,4518	0,6048	0,5419	0,6064	0,6056	0,6122	0,6213	0,6346	0,6249	0,5790	0,5091	0,4896
	0,4546	0,6030	0,5455	0,5857	0,6074	0,6298	0,6354	0,6355	0,6201	0,5713	0,5182	0,4913
Absorbance 600nm	0,5738	0,4401	0,5407	0,4647	0,4472	0,4425	0,4408	0,4207	0,4383	0,4932	0,6266	0,6122
	0,5792	0,4398	0,5475	0,4841	0,4556	0,4636	0,4503	0,4315	0,4484	0,5141	0,6375	0,6175
Absorbance difference	-0,1220	0,1647	0,0012	0,1417	0,1584	0,1697	0,1805	0,2139	0,1866	0,0858	-0,1175	-0,1226
	-0,1246	0,1632	-0,0020	0,1016	0,1518	0,1662	0,1851	0,2040	0,1717	0,0572	-0,1193	-0,1262
% live cell		117,58	50,83	108,19	115,00	119,62	124,03	137,66	126,52	85,36	2,37	0,29
		116,96	49,52	91,81	112,31	118,19	125,90	133,62	120,43	73,69	1,63	-1,18
Average % live cell		117,27	50,17	100,00	113,66	118,90	124,96	135,64	123,47	79,53	2,00	-0,45
SEM		0,31	0,65	8,19	1,35	0,71	0,94	2,02	3,04	5,84	0,37	0,73
Normalized data % live cell		117,27	50,17	100,00	113,66	118,90	124,96	135,64	123,47	79,53	2,00	-0,45

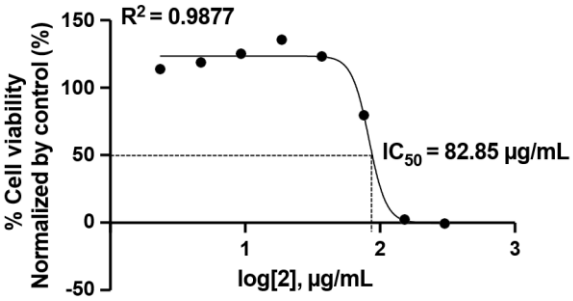

Media	Media +cell	Cisplatin	Solvent	Sample concentrations (µg/mL)							
				2,34	4,69	9,38	18,75	37,50	75,00	150,00	300,00

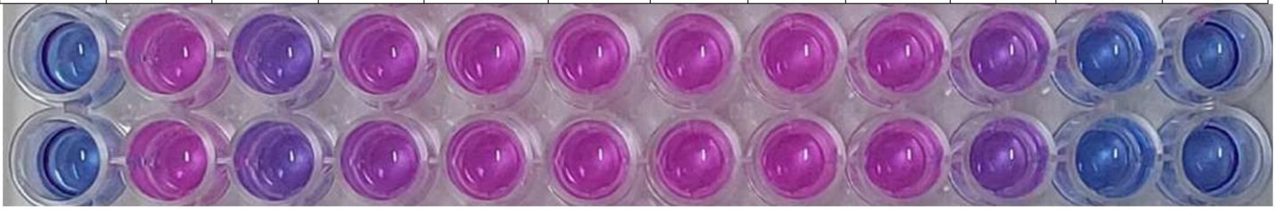

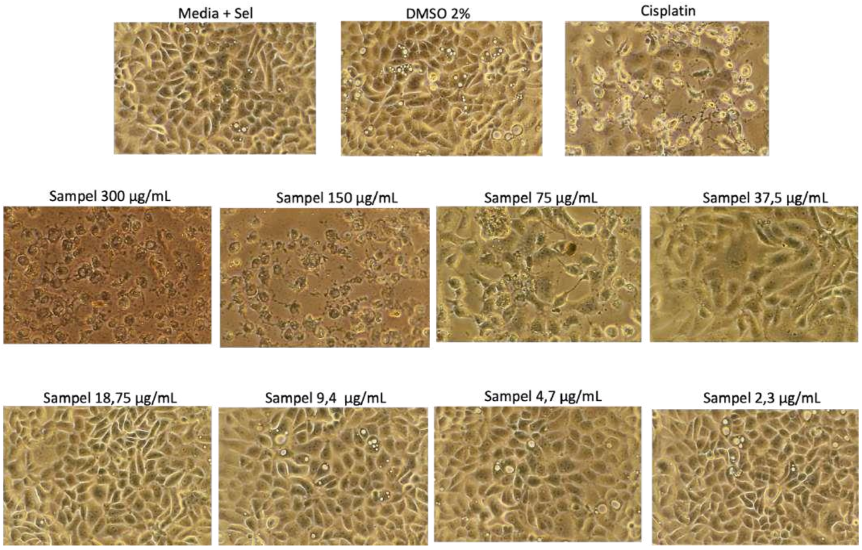

CV-1 cell morphology at each concentration of compound **2**

Figure S54. Results of cytotoxic activity of **3** against CV-1 cell line.

	Media	Media + cell	Cisplatin	Solvent	Sample concentrations (µg/mL)							
					2,34	4,69	9,38	18,75	37,50	75,00	150,00	300,00
Absorbance 570nm	0,4165	0,6597	0,5592	0,6652	0,6441	0,6231	0,6520	0,6504	0,6416	0,6214	0,6311	0,6229
	0,4796	0,6565	0,4082	0,6498	0,6503	0,6287	0,7295	0,6420	0,6444	0,6658	0,6517	0,6498
Absorbance 600nm	0,5388	0,3514	0,4926	0,3588	0,3832	0,3702	0,3580	0,3722	0,3713	0,3975	0,4297	0,5089
	0,6217	0,3466	0,3182	0,3662	0,3656	0,3496	0,4347	0,3904	0,3743	0,3769	0,4040	0,4508
Absorbance difference	-0,1223	0,3083	0,0666	0,3064	0,2609	0,2529	0,2940	0,2782	0,2703	0,2239	0,2014	0,1140
	-0,1421	0,3099	0,0900	0,2836	0,2847	0,2791	0,2948	0,2516	0,2701	0,2889	0,2477	0,1990
% live cell		103,11	46,54	102,67	92,02	90,15	99,77	96,07	94,22	83,36	78,09	57,63
		103,49	52,01	97,33	97,59	96,28	99,95	89,84	94,17	98,57	88,93	77,53
Average % live cell		103,30	49,27	100,00	94,80	93,21	99,86	92,95	94,19	90,96	83,51	67,58
SEM		0,19	2,74	2,67	2,79	3,07	0,09	3,11	0,02	7,61	5,42	9,95
Normalized data % live cell		103,30	49,27	100,00	94,80	93,21	99,86	92,95	94,19	90,96	83,51	67,58

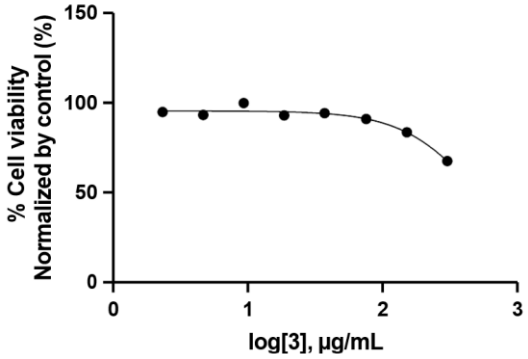

Media	Media +cell	Cisplatin	Solvent	Sample concentrations (µg/mL)							
				2,34	4,69	9,38	18,75	37,50	75,00	150,00	300,00

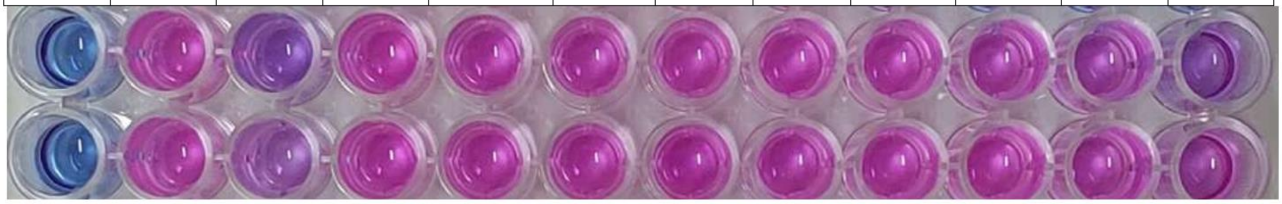

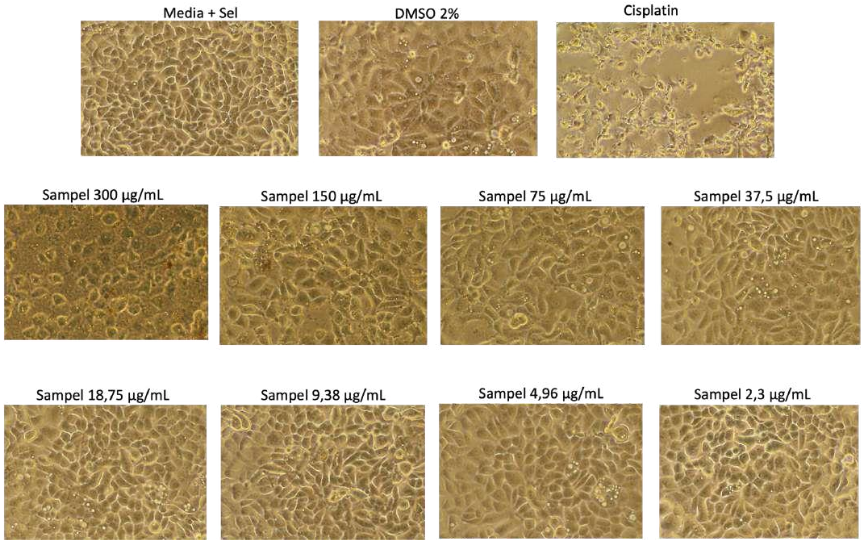

CV-1 cell morphology at each concentration of compound **3**

Figure S55. Results of cytotoxic activity of **4** against CV-1 cell line.

	Media	Media + cell	Cisplatin	Solvent	Sample concentrations (µg/mL)							
					2,34	4,69	9,38	18,75	37,50	75,00	150,00	300,00
Absorbance 570nm	0,4640	0,6657	0,5603	0,6378	0,6427	0,6352	0,6466	0,6474	0,6526	0,6323	0,5216	0,5649
	0,4727	0,6893	0,5760	0,6586	0,6534	0,6664	0,6475	0,6099	0,6818	0,6544	0,5656	0,4729
Absorbance 600nm	0,6025	0,3511	0,4973	0,3726	0,3847	0,3705	0,3526	0,3531	0,3805	0,3779	0,5391	0,7128
	0,6115	0,3249	0,5070	0,3652	0,3608	0,3480	0,3687	0,3207	0,3496	0,4067	0,5019	0,5969
Absorbance difference	-0,1385	0,3146	0,0630	0,2652	0,2580	0,2647	0,2940	0,2943	0,2721	0,2544	-0,0175	-0,1479
	-0,1388	0,3644	0,0690	0,2934	0,2926	0,3184	0,2788	0,2892	0,3322	0,2477	0,0637	-0,1240
% live cell		108,45	48,25	96,63	94,90	96,51	103,52	103,59	98,28	94,04	28,99	-2,21
		120,36	49,68	103,37	103,18	109,36	99,88	102,37	112,66	92,44	48,41	3,51
Average % live cell		114,40	48,97	100,00	99,04	102,93	101,70	102,98	105,47	93,24	38,70	0,65
SEM		5,96	0,72	3,37	4,14	6,42	1,82	0,61	7,19	0,80	9,71	2,86
Normalized data % live cell		114,40	48,97	100,00	99,04	102,93	101,70	102,98	105,47	93,24	38,70	0,65

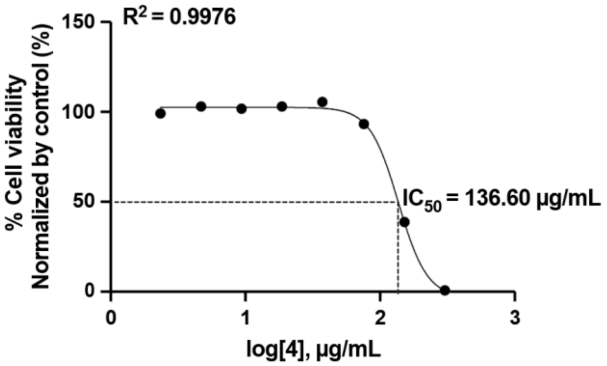

Media	Media +cell	Cisplatin	Solvent	Sample concentrations (µg/mL)							
				2,34	4,69	9,38	18,75	37,50	75,00	150,00	300,00

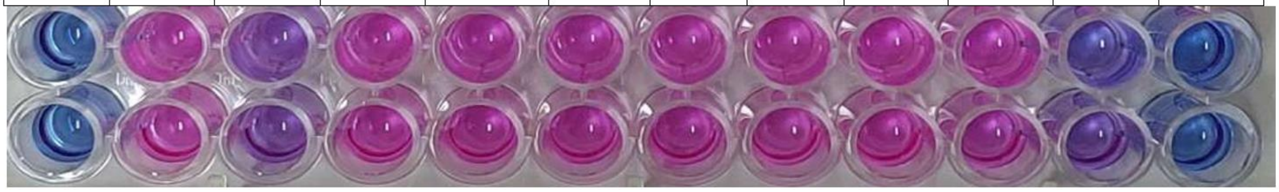

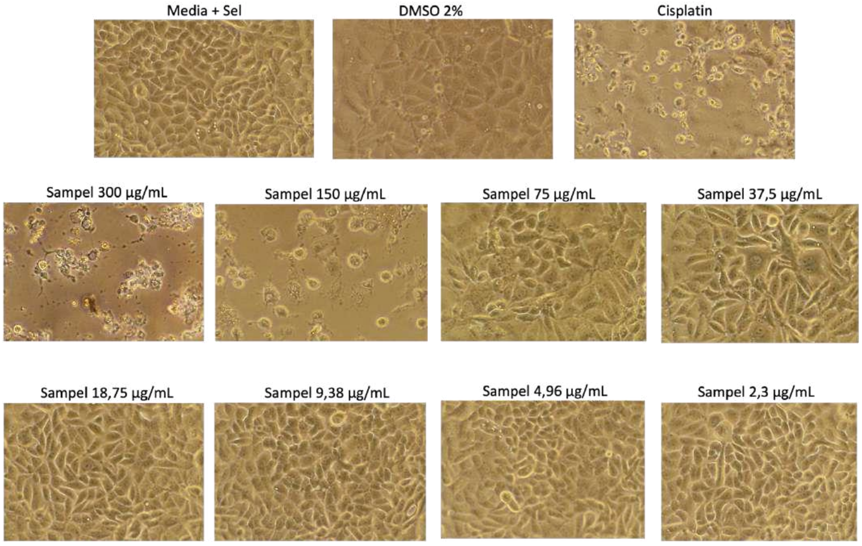

CV-1 cell morphology at each concentration of compound **4**

Figure S56. Results of cytotoxic activity of **5** against CV-1 cell line.

	Media	Media + cell	Cisplatin	Solvent	Sample concentrations (µg/mL)							
					2,34	4,69	9,38	18,75	37,50	75,00	150,00	300,00
Absorbance 570nm	0,4687	0,6433	0,5625	0,6487	0,6356	0,6404	0,6260	0,6342	0,6365	0,6495	0,6546	0,6657
	0,4708	0,6554	0,5644	0,6482	0,6192	0,6210	0,6440	0,6267	0,6308	0,6475	0,6577	0,6626
Absorbance 600nm	0,6093	0,3598	0,5112	0,3601	0,3625	0,3558	0,3648	0,3510	0,3416	0,3308	0,3360	0,3254
	0,6111	0,3561	0,5117	0,3670	0,3697	0,3756	0,3544	0,3410	0,3660	0,3619	0,3469	0,3298
Absorbance difference	-0,1406	0,2835	0,0513	0,2886	0,2731	0,2846	0,2612	0,2832	0,2949	0,3187	0,3186	0,3403
	-0,1403	0,2993	0,0527	0,2812	0,2495	0,2454	0,2896	0,2857	0,2648	0,2856	0,3108	0,3328
% live cell		99,67	45,08	100,87	97,23	99,93	94,43	99,60	102,35	107,95	107,92	113,02
		103,39	45,41	99,13	91,68	90,71	101,10	100,19	95,27	100,16	106,09	111,26
Average % live cell		101,53	45,25	100,00	94,45	95,32	97,77	99,89	98,81	104,06	107,01	112,14
SEM		1,86	0,16	0,87	2,77	4,61	3,34	0,29	3,54	3,89	0,92	0,88
Normalized data % live cell		101,53	45,25	100,00	94,45	95,32	97,77	99,89	98,81	104,06	107,01	112,14

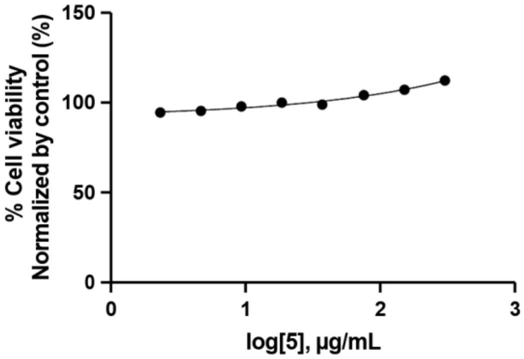

Media	Media +cell	Cisplatin	Solvent	Sample concentrations (µg/mL)							
				2,34	4,69	9,38	18,75	37,50	75,00	150,00	300,00

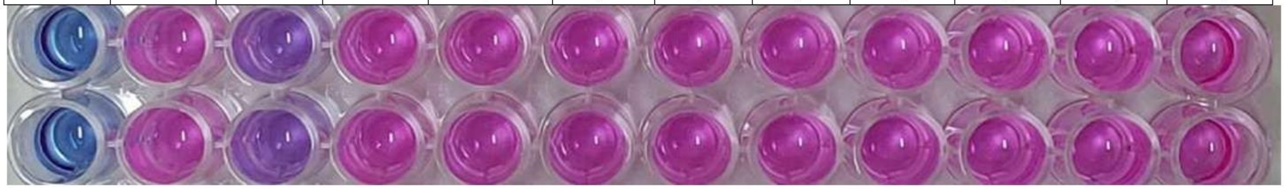

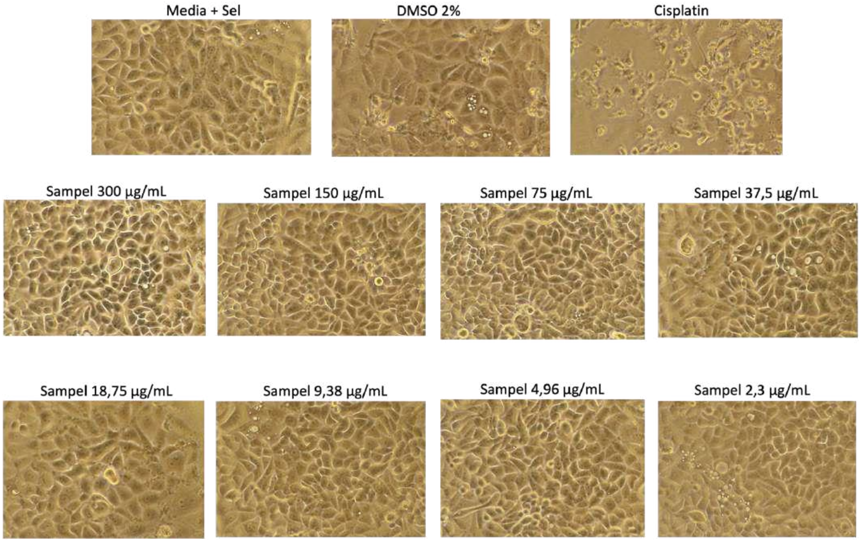

CV-1 cell morphology at each concentration of compound **5**

Figure S57. Results of cytotoxic activity of **6** against CV-1 cell line.

	Media	Media + cell	Cisplatin	Solvent	Sample concentrations (µg/mL)							
					2,34	4,69	9,38	18,75	37,50	75,00	150,00	300,00
Absorbance 570nm	0,5265	0,7176	0,6352	0,7629	0,7335	0,7533	0,7533	0,7493	0,7956	0,6623	0,6026	0,5243
	0,5327	0,7246	0,6209	0,7721	0,7248	0,7415	0,7439	0,7256	0,7917	0,7524	0,6552	0,5266
Absorbance 600nm	0,5257	0,2722	0,4256	0,2385	0,2696	0,2757	0,2699	0,2697	0,2211	0,3672	0,4578	0,5227
	0,5322	0,2699	0,4184	0,2511	0,2693	0,2728	0,2685	0,2560	0,2214	0,3031	0,4340	0,5217
Absorbance difference	0,0008	0,4454	0,2096	0,5244	0,4639	0,4776	0,4834	0,4796	0,5745	0,2951	0,1448	0,0016
	0,0005	0,4547	0,2025	0,5210	0,4555	0,4687	0,4754	0,4696	0,5703	0,4493	0,2212	0,0049
% live cell		98,97	46,50	116,54	103,08	106,13	107,42	106,58	127,69	65,52	32,08	0,21
		101,03	44,92	115,79	101,21	104,15	105,64	104,35	126,76	99,83	49,08	0,95
Average % live cell		100,00	45,71	116,17	102,15	105,14	106,53	105,46	127,23	82,68	40,58	0,58
SEM		1,03	0,79	0,38	0,93	0,99	0,89	1,11	0,47	17,16	8,50	0,37
Normalized data % live cell		86,08	39,34	100,00	87,93	90,51	91,71	90,79	109,52	71,17	34,93	0,50

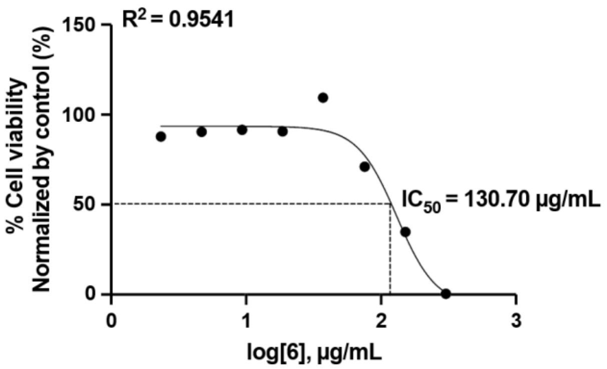

Media	Media +cell	Cisplatin	Solvent	Sample concentrations (µg/mL)							
				2,34	4,69	9,38	18,75	37,50	75,00	150,00	300,00

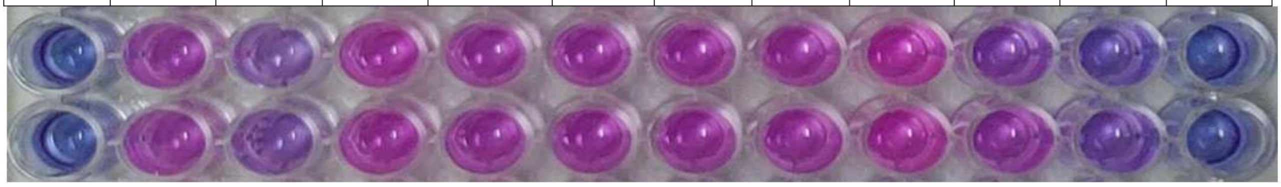

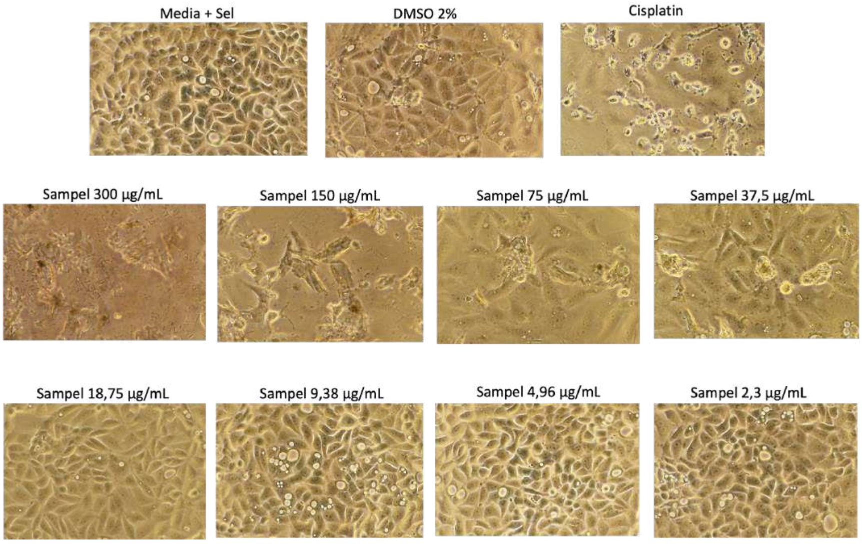

CV-1 cell morphology at each concentration of compound **6**

Figure S58. Results of cytotoxic activity of **7** against CV-1 cell line.

	Media	Media + cell	Cisplatin	Solvent	Sample concentrations (µg/mL)							
					2,34	4,69	9,38	18,75	37,50	75,00	150,00	300,00
Absorbance 570nm	0,5242	0,6329	0,5616	0,6183	0,6212	0,6140	0,6131	0,6115	0,6205	0,6185	0,6231	0,6225
	0,4539	0,6153	0,5522	0,6095	0,6111	0,6084	0,6150	0,6161	0,6215	0,6760	0,6400	0,6143
Absorbance 600nm	0,6704	0,4350	0,5451	0,4271	0,4531	0,4413	0,4347	0,4414	0,4373	0,4385	0,4509	0,4594
	0,5781	0,4504	0,5541	0,4498	0,4549	0,4511	0,4572	0,4523	0,4644	0,4833	0,4874	0,4562
Absorbance difference	-0,1462	0,1979	0,0165	0,1912	0,1681	0,1727	0,1784	0,1701	0,1832	0,1800	0,1722	0,1631
	-0,1242	0,1649	-0,0019	0,1597	0,1562	0,1573	0,1578	0,1638	0,1571	0,1927	0,1526	0,1581
% live cell		107,23	48,83	105,07	97,63	99,11	100,95	98,28	102,49	101,46	98,95	96,02
		96,60	42,91	94,93	93,80	94,16	94,32	96,25	94,09	105,55	92,64	94,41
Average % live cell		101,92	45,87	100,00	95,72	96,64	97,63	97,26	98,29	103,51	95,80	95,22
SEM		5,31	2,96	5,07	1,92	2,48	3,32	1,01	4,20	2,04	3,15	0,80
Normalized data % live cell		101,92	45,87	100,00	95,72	96,64	97,63	97,26	98,29	103,51	95,80	95,22

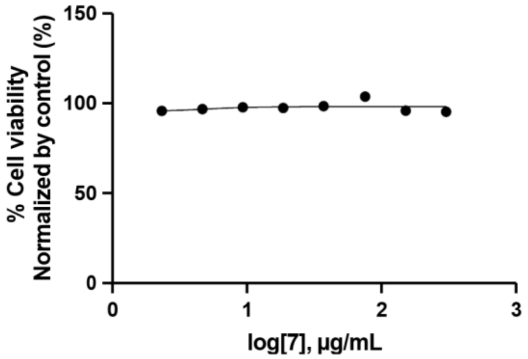

Media	Media +cell	Cisplatin	Solvent	Sample concentrations (µg/mL)							
				2,34	4,69	9,38	18,75	37,50	75,00	150,00	300,00

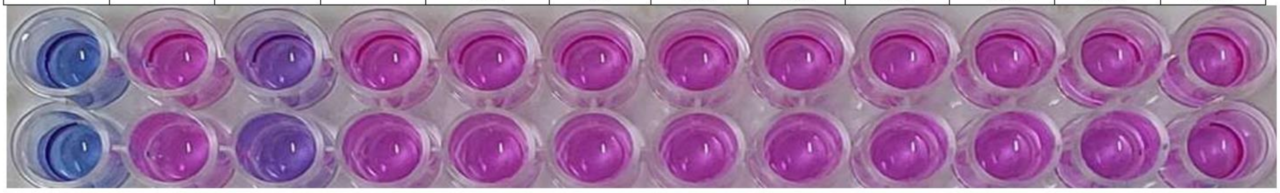

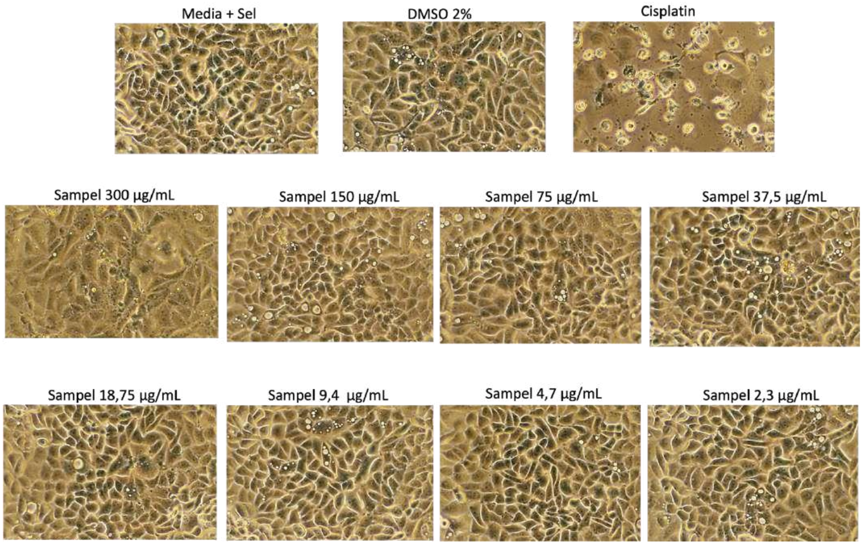

CV-1 cell morphology at each concentration of compound **7**

Figure S59. TLC profile of compounds **1**-**7**.

After sprayed with H_2SO_4 in EtOH followed by heating

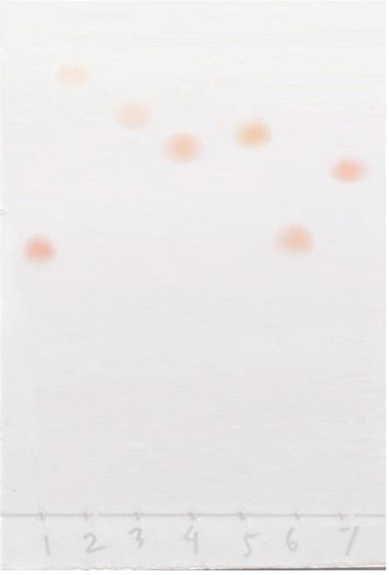

Under UV 365 nm

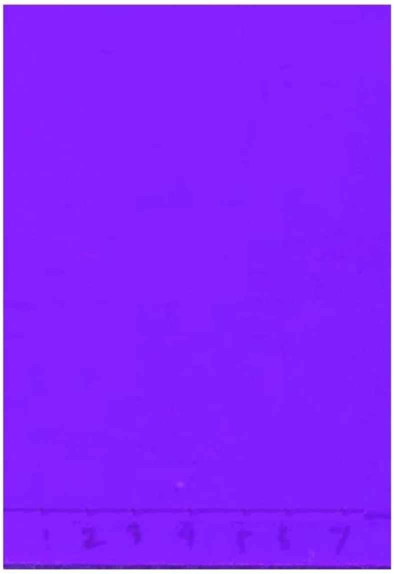

Under UV 254 nm

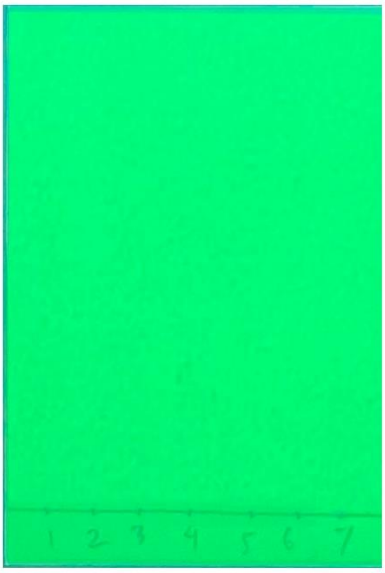

Supplement: Supplementary file 1 [file molecules-28-04946-s001.zip › molecules-2436588-supplementary.pdf]
